# Supplementary material for: Unveiling biogeographical patterns of the ichthyofauna in the Tuichi basin, a biodiversity hotspot in the Bolivian Amazon, using environmental DNA
Source: PLoS One. 2022 Jan 4;17(1):e0262357. doi: 10.1371/journal.pone.0262357 (PMC8726463; doi:10.1371/journal.pone.0262357)

Figure S1 : Rarefaction curves

Figure A & B : Rarefied taxa accumulation curves by read count per librairie (A) and per site (B). Vignet are labelled corresponding to their site location.

A

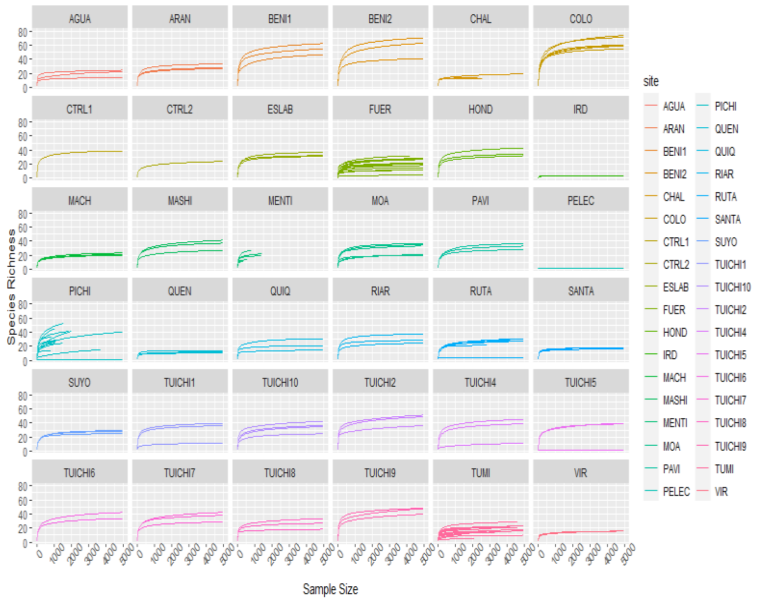

B

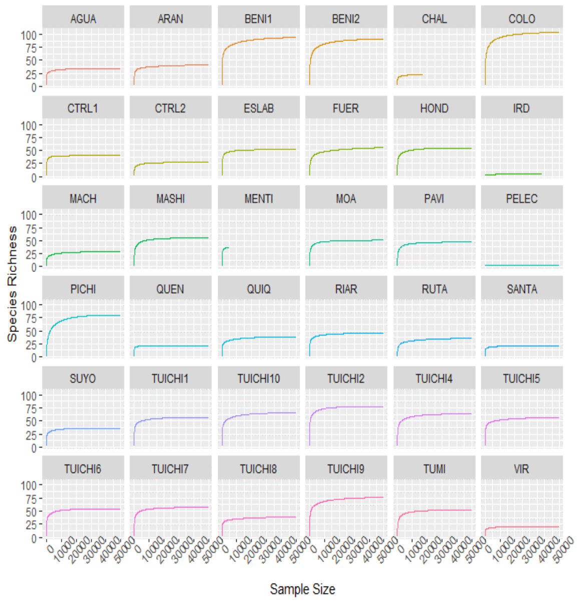

Supplement: S1 Fig — (PDF) [file pone.0262357.s001.pdf]
