# Supplementary material for: Unveiling biogeographical patterns of the ichthyofauna in the Tuichi basin, a biodiversity hotspot in the Bolivian Amazon, using environmental DNA
Source: PLoS One. 2022 Jan 4;17(1):e0262357. doi: 10.1371/journal.pone.0262357 (PMC8726463; doi:10.1371/journal.pone.0262357)

Figure S2 : Frequency spectrum of taxa

Frequency spectrum of taxa as a function of the number of sites

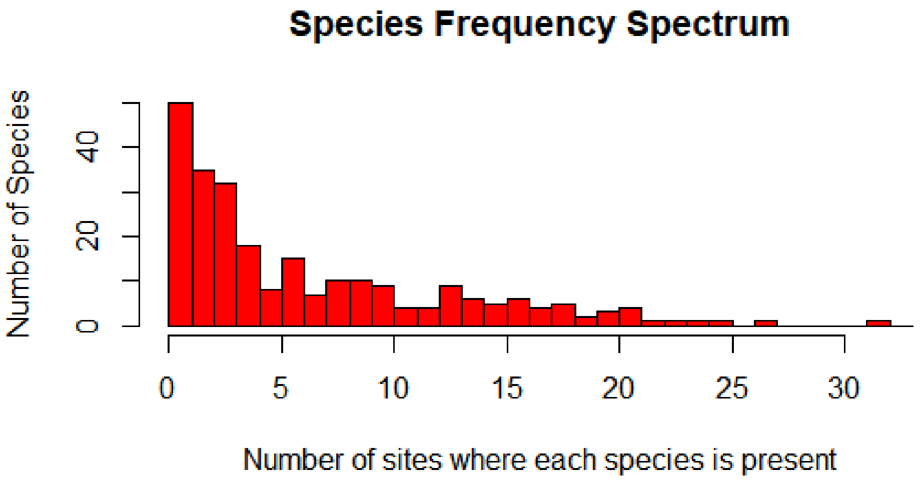

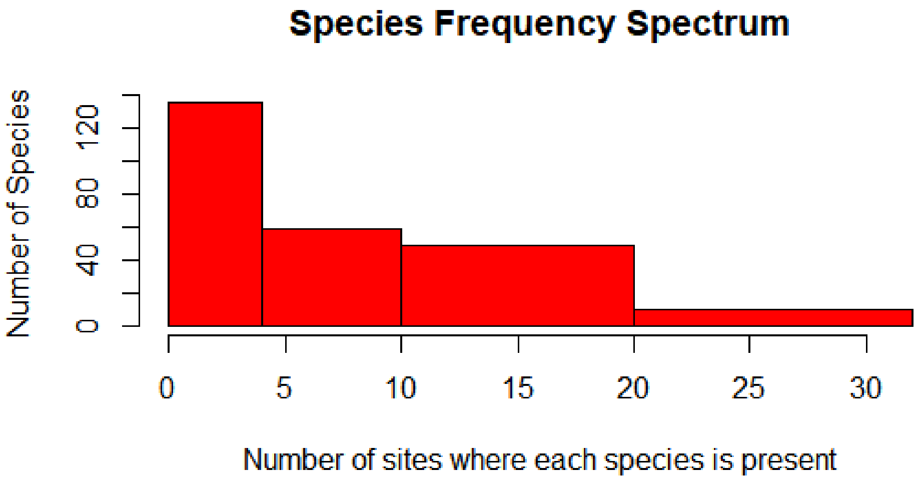

Supplement: S2 Fig — (PDF) [file pone.0262357.s002.pdf]
