# Supplementary figures and images for: Unveiling biogeographical patterns of the ichthyofauna in the Tuichi basin, a biodiversity hotspot in the Bolivian Amazon, using environmental DNA
Source: PLoS One. 2022 Jan 4;17(1):e0262357. doi: 10.1371/journal.pone.0262357 (PMC8726463; doi:10.1371/journal.pone.0262357)

Figure S3 : Taxonomic diversity at Order level per site

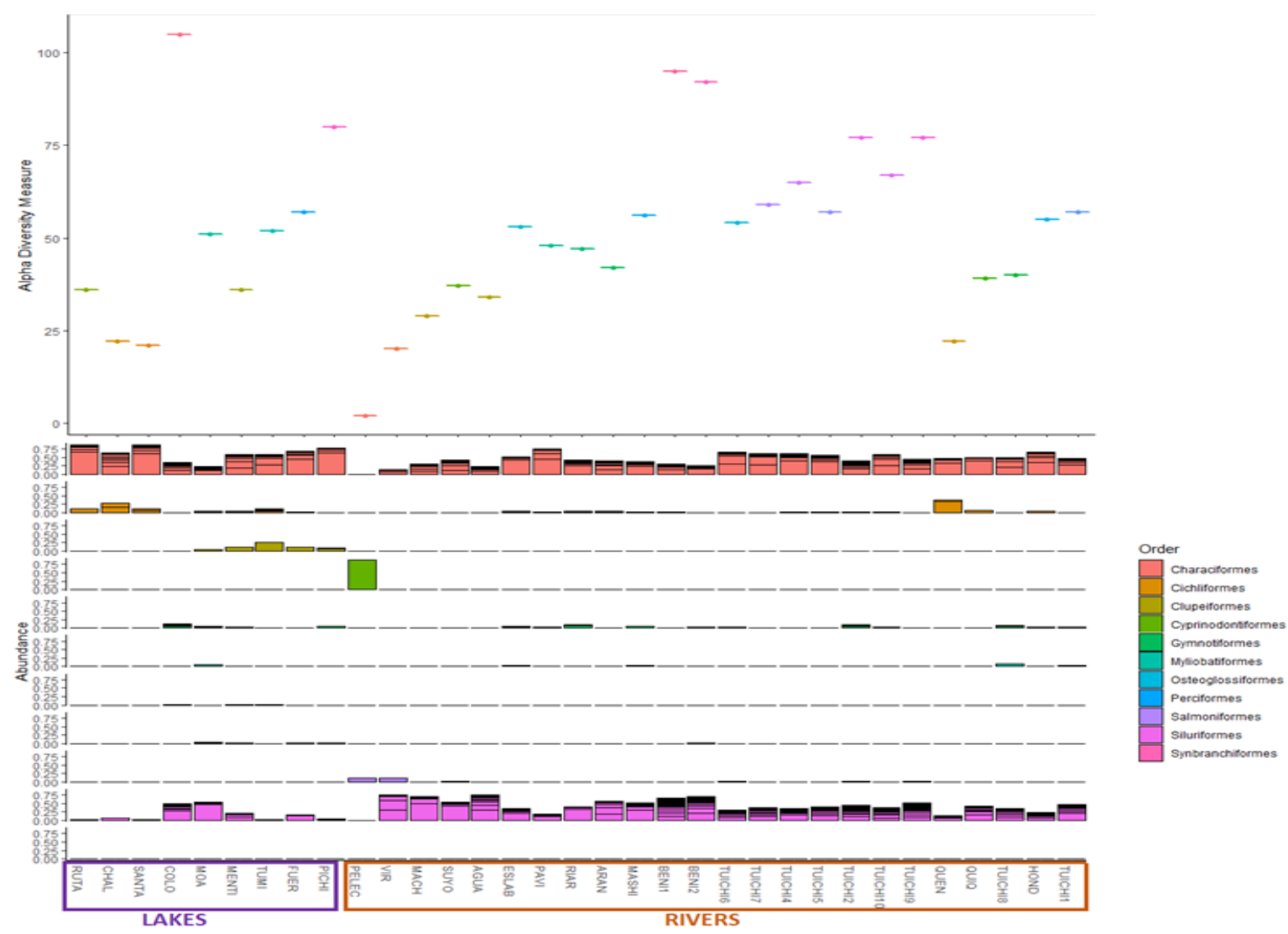

Supplement: S3 Fig — (PDF) [file pone.0262357.s003.pdf]

Figure S5: NMDS ordination including all sites. Orders are split into different facets

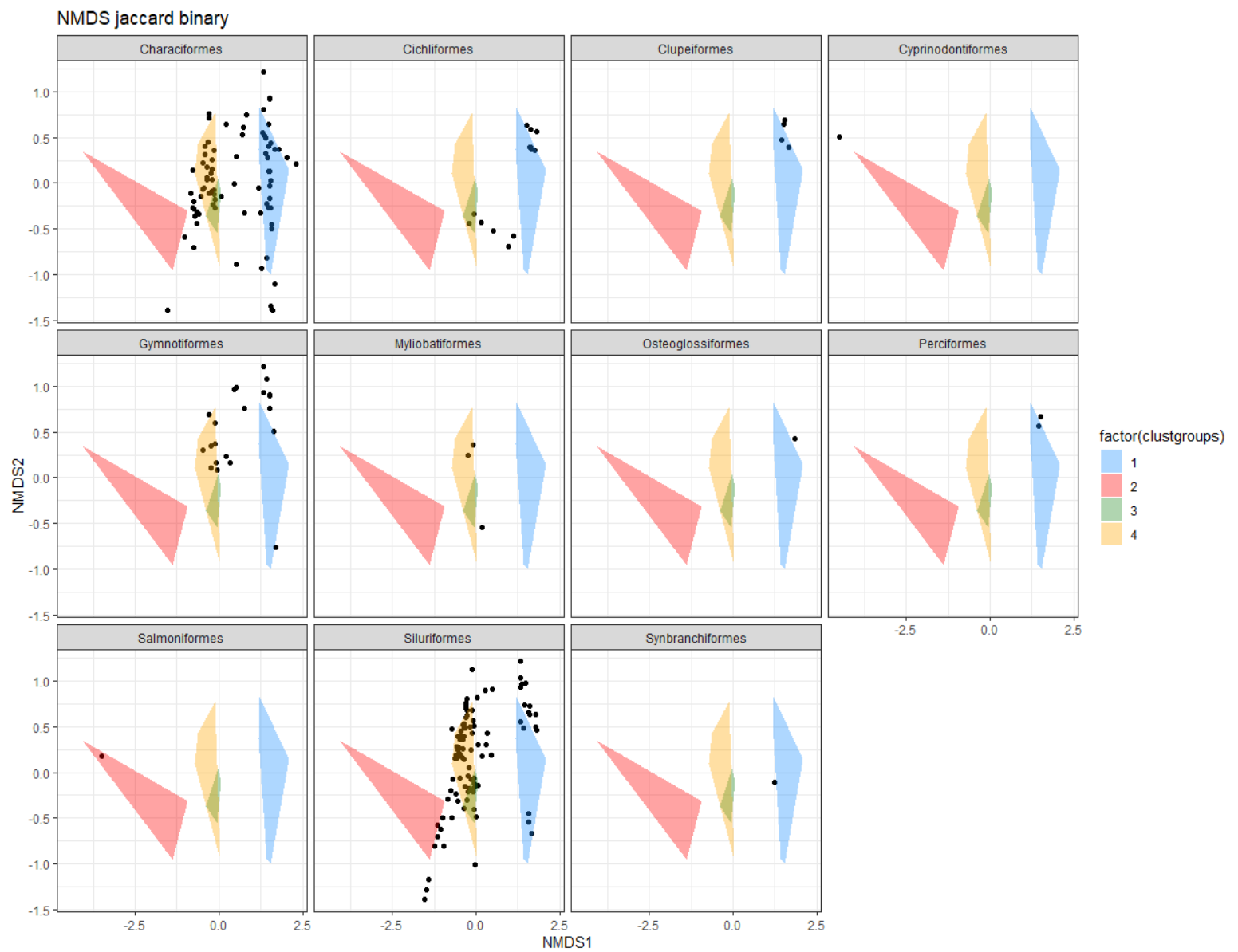

Supplement: S5 Fig — Orders are split into different facets. (PDF) [file pone.0262357.s005.pdf]

Figure S6: Species dendrogram (Binary Jaccard distance and Ward2 aggregation)

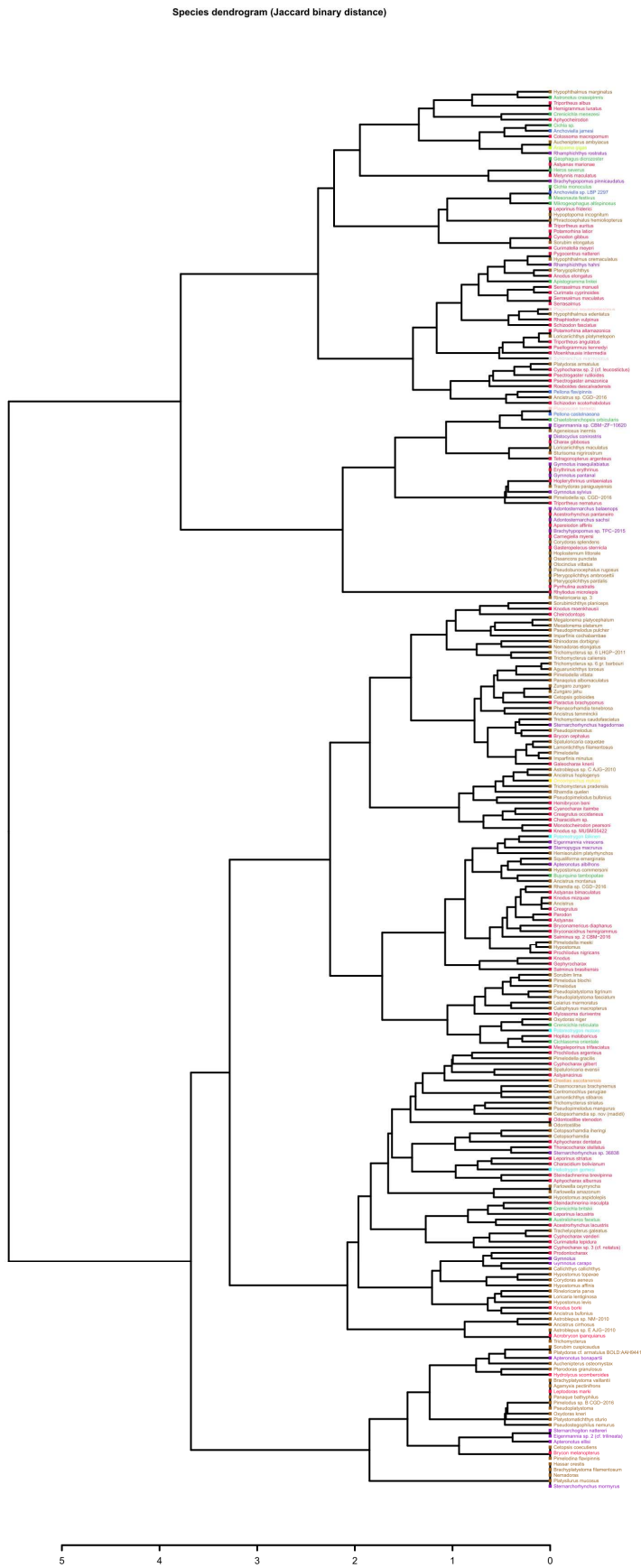

Supplement: S6 Fig — (PDF) [file pone.0262357.s006.pdf]
