# Supplementary material for: Unveiling biogeographical patterns of the ichthyofauna in the Tuichi basin, a biodiversity hotspot in the Bolivian Amazon, using environmental DNA
Source: PLoS One. 2022 Jan 4;17(1):e0262357. doi: 10.1371/journal.pone.0262357 (PMC8726463; doi:10.1371/journal.pone.0262357)

Figure S4 : NMDS ordination

NMDS ordination of sample sites based on taxon composition (Jaccard's binary distance). Ordination was performed on all sampled sites, including lakes and rivers. The ellipses are colour coded according to the 4 cluster groups identified by HAC with yellow, green and red for rank 1 or 2 rivers, rank 3 rivers and Alto-Tuichi, respectively and a blue convex hull for lakes. Note the position of the QUEN site very far from its cluster (see discussion in the article).

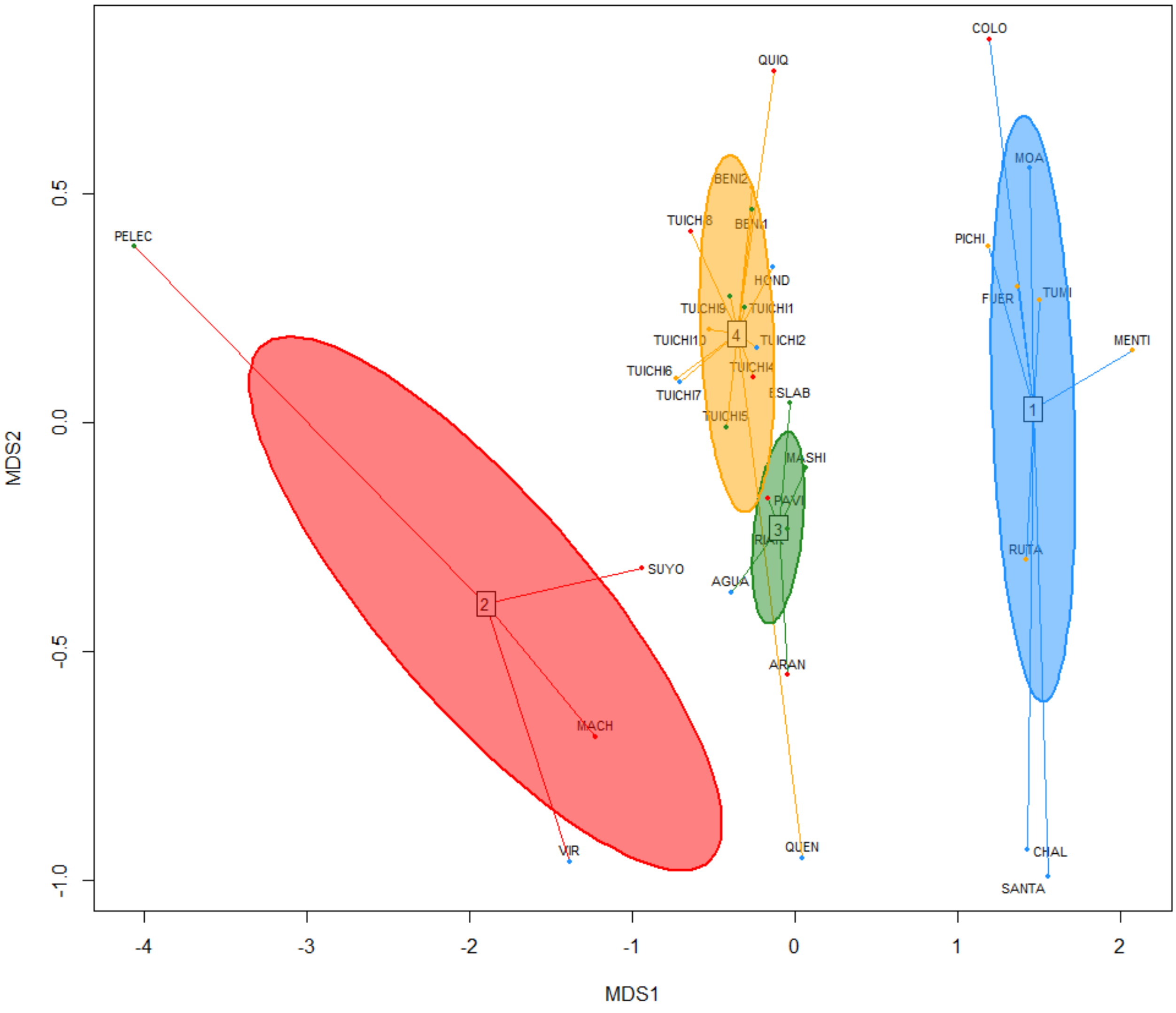

Supplement: S4 Fig — (PDF) [file pone.0262357.s004.pdf]
