# Supplementary material for: Unveiling biogeographical patterns of the ichthyofauna in the Tuichi basin, a biodiversity hotspot in the Bolivian Amazon, using environmental DNA
Source: PLoS One. 2022 Jan 4;17(1):e0262357. doi: 10.1371/journal.pone.0262357 (PMC8726463; doi:10.1371/journal.pone.0262357)
Supplement: S1 Text — (PDF) [file pone.0262357.s007.pdf]

Script_eDNA_for_publication

Mariac Cedric

14/04/2021

Contents

Construction of the COI fish reference database 5
Import data from BOLD 5
Import data from Genebank 6
Remove Bad taxa 6
Remove redondancy 6
Merge BOLD + NCBI 6
Additionnal sequences in DB 7
Remove references that do not have at least a sp. Name 7
Reference header formatting: simplification of reference names from Bold 7
Final file DB COI used : # Construction of the COI fish reference database 7

Import sequencing Data from Novogene & preformatting 7

Barcodes informations and clustering 7
FROGS Options 7
Barcodes, mk1, mk2 8
MK1 185 bp 8
MK2 285 bp 8
FROGS for mk1 8
FROGS for mk2 8
Length distribution of the clusters 8

Blastn OTU clusters 8
run blastn (2.8.1+) on DBCOIv9 (only fish) 9
run blastn (2.8.1+) on NCBI local database (all taxa) 9

MEGAN assignation parameters 9

Decontamination (substraction with microdecon package) **10**

Input file format for microdecon 10

Run Microdecon 10

RUN PHYLOSEQ **11**

Loading libraries 11

Import taxmat (Taxonomy) 12

Import OTU 12

Import metadata 12

CompilationN taxmat, OTU & metadata 13

Filtering OTUs 13

Count number of spec. genus over all sample including controls 14

Remove Controls 14

Count at different taxrank over all sample (excluding controls) 14

Merge samples at site level 15

Merge samples at Biogeographic entities level 15

Repair the merged values associated with each site after merge. 15

Transform to % 15

Count number of reads per lib 15

RAREFACTION CURVES per site 16

RAREFACTION CURVES at lib level 17

Count in how many site taxa are present 17

ALPHA diversity **18**

NMDS on Libraries 19

Plot richness per LIB 20

Plot richness per SITE 21

Barplot taxo diversity per site 22

Barplot taxo diversity per site (facet View) 23

Compare RIVER and LAKE 24

Venn Diagram 25

Retreived specific Order to Rivers or lakes 28

ANOVA on Shannon index versus Site or ECOTYE 29

MAXSSS **31**
Import DATA . . . 31
Convert data in ROCR format . . . 32
Analysis of performance on tpr and fpr then on specificity . . . 32
Identify the 'optimal' cutoff . . . 32
ROC curves . . . 32

Expected richness **33**
Extract OTU to dataframe . . . 33
Extract sample data to a dataframe . . . 33
Richness & expected richness on the desired hierarchical level . . . 34
Expected Richness over all Sites ; number of species per "pool" (eg site or ECOTYPE . . .) . . . 35
Observed Richness per site . . . 35
Accumulation model over all Sites . . . 35
BOXPLOT : richness Observed per site, coverage per site . . . 36
Richness at site level :"Observed", "Shannon","Simpson","ACE" . . . 37

Compute Mean percent ID per taxa **37**
Create a txt file with these 3 columns : new.txt . . . 38
Split Dataset in a table as : . . . 38

Correlation Altitude & richness **38**
Correlation and test . . . 39

Plot of Observed, extrapolated (Jack2) richness and percentage of covered richness **39**
At site level . . . 39
FIGURE 2 : At biogeographic entities level (include all sites) . . . 40
Test percentage of coverage between Biogeographic entities . . . 42
Test percentage of coverage between sites . . . 45
Return CI of coverage per site . . . 56
Extract data from physeq5 object : . . . 57
Built dendrogram without Boot . . . 58
Built dendro on sites (translate matrix) . . . 58
Built dendro on taxas (Original matrix) . . . 58
Built dendrogram with Boot . . . 59
Built dendro on sites (translate matrix) . . . 59
Built dendro on taxas (Original matrix) . . . 60
Inertia . . . 61
Standardize abundances . . . 61
FIGURE4 : HEATMAP . . . 62

NMDS ordination + environmental variables VEGAN **65**
Notes 65
Loading Libraries and function 66
NMDS ALL SITES (using PHYLOSEQ) 66
Import data 66
Dendrogram 66
Extract scrs 67
Define color vector 68
Extract richness for futur point sizing 68
Preliminary graph 68
Search for environmental variables that are significantly correlated with NMDS.: 69
FIGURE 6A : NMDS & environmental variables All sites 72
NMDS RIVERS sites (using PHYLOSEQ) 74
Import data 74
Run ordination on the generated distance 74
Extract scrs 75
Define color vector 75
Extract richness for futur point sizing 75
Preliminary graph 76
Search for environmental variables that are significantly correlated with NMDS 76
FIGURE 6B : NMDS & environmentales variables RIVERS sites 78
NMDS LAKES Sites (using PHYLOSEQ) 79
Import data 79
Run ordination on the generated distance 79
Extract scrs 80
Define color vector 80
Extract richness for futur point sizing 80
Preliminary graph 81
Plot Vegan + surf on hydrologigal network size 82
Search for environmental variables that are significantly correlated with NMDS 83
Significative var 84
FIGURE 6C : NMDS & environmentales variables LAKE 85
NOT USED : NMDS under PHYLOSEQ subset analysis on RIVERS of RANKIII only 86
Run ordination on the generated distance 86
Draw significative environmental variables = NONE are significatives 87
FIGURE 6X : NMDS & environmentales variables Rivers Rank3 87

Plot NMDS ordination Altitude iso lines **88**
Input data . . . 88
Extract richness for futur point sizing . . . 88
NMDS Ordination . . . 88
combine NMDS points and metadata . . . 89
Ordisurf . . . 89
Plotting . . . 90

Plot NMDS ordination, Order facet and species contributions **91**
Input data . . . 91
Plotting bi-plot in ordination space . . . 91
SFIGURE3 . . . 92
corelation between var . . . 93

Compare % of Characiformes / Siluriformes in Lakes and Rivers **93**
Data . . . 94
Import data and test . . . 94

ADONIS : test for cluster differences in ordination plot **94**
Input data (Phyloeq object) . . . 94
Export OTU and Metadata in data.frame . . . 94
Ordination Using phyloseq . . . 94
Plot ordination ellipses, spider, ordisurf . . . 95
test for differences in community among the clustergroups . . . 96
Adonis on OTU table directly . . . 96
Adonis on distance matrix . . . 97
Homogeneity of dispersion test (critical assumption here is that variance is egal among the clustgroups) 97
using anova . . . 97
Using permanova . . . 97

Annexe1: FROGS sample file script (slurm) **98**

Annexe2: BLASTn sample file script (slurm) **102**

Construction of the COI fish reference database

Import data from BOLD

```
res <- bold_seqspec(taxon = "Actinopterygii", marker = "COI-5P")
```

Import data from Genebank

```
((("Actinopterygii"[Organism] AND COI[gene]) OR ("Actinopterygii"[Organism] AND
                                                 COX[gene])
  OR("Actinopterygii"[Organism] AND "cytochrome oxidase subunit"[All Fields]) OR
  ("Actinopterygii"[Organism] AND "cytochrome c oxidase subunit"[All Fields]) OR
  ("Actinopterygii"[Organism] AND Cox[gene])) AND 300:2000[Sequence Length]) NOT
NADH NOT
"subunit2" NOT "subunit 2" NOT "subunitII" NOT "subunit II" NOT "subunit3" NOT
"subunit 3" NOT
"subunitIII" NOT "subunit III"
```

Remove Bad taxa

KU692455, KU692454 , KU692453 = Colossoma macropomum : probably bad ID = same sequence as piaractus
Colossoma macropomum (7,8,9)(IIAP) = Piaractus meso (supp Colossoma)
HQ689374.1 Brachyplatystoma sp. 'Surubim ? Brachy sp. = invalide name
GU060426. = Curimata inornata because it connects with several semaprochilodus
EU185608.1 ; EU185609.1 = Schizodon_intermedius : probably bad ID (tree) Bold identifies them as leporinus
FJ918909.1 = Colossoma sp. KU 3081 doubtful quality sequence (indels) secondary assignment = piaractus
Semaprochilodus insignis (IIAP) = Shizodon fasciatus
NC015813.1 Hydrolycus_scomberoides redundant with Hoplerythrinus_unitaeniatus Hopun01
Hoplerytrhrinus Uni numero 9
Semaprochilodus insignis (data from Carmen presumably Shizodon fasciatus)
gi|14324128|gb|AY034153.1|tax|59899| Clarias batrachus gi|311718554|gb|HP587557.1|tax|7998| TSA: Ictalurus punctatus Contig08262.
remove 94 tempo sequence sent by IIAP.
Seriolella porosa NC_025665 (coi bold) = not Seriolella but possible piaractus meso
Apareiodon affinis | LARI249-13 because is undoubtedly Ageneiosus inermis

Remove redondancy

Remove in the NCBI sequence list the sequences already available under Bold
Extract the genebank codes from the Bold file, make a list = ID.txt
Export NCBI-COI-16032018.fasta in tab format (NCBI-COI-16032018.fasta.tab)

```
grep -vF -f ID.txt NCBI.fasta.tab> clean.NCBI.fasta.tab
convert clean.NCBI-COI-16032018.fasta.tab en fasta -> clean.NCBI-COI-16032018.fasta
```

Merge BOLD + NCBI

Clean.NCBI-COI-16032018.fasta + Bold-extraction-COI-15032018.fasta

```
cat Bold-extraction-COI-15032018.fasta clean.NCBI-COI-16032018.fasta >
```

Additionnal sequences in DB

Diplomystes nahuelbutaensis (AP012011) : COI extract from full mitogenome
Add 271 sequences sent by IIAP filed under GNB but not public.

Remove references that do not have at least a sp. Name

open in excel DB-COI-16032018.fasta // convert tab and sort to remove unassigned species
deletion of 40825 unspecified sequences at the species level

Reference header formatting: simplification of reference names from Bold

Format as follow = >species_name | bin_uri markercode | genbank_accession
">Abalistes stellatus | DSFSF624-09 | COI-5P | JF492756"

Final file DB COI used : # Construction of the COI fish reference database

DB - COI - 02012020 - v9.fasta

Import sequencing Data from Novogene & preformatting

Wget https://s3.eu-west-1.amazonaws.com/novogene-europe/HW/project/X201SC19111401-Z01-F001_oFRbyM.tar

```
## 1/ extract tar
tar -xvf  X201SC19111401-Z01-F001_oFRbyM.tar
## 2/ move all gz in a new directory
find ../raw_data/ -iname "*.fq.gz" -type f -print0 | xargs -0 -I "{}" mv {} ./data/
## 6/ count
for i in *.gz ; do echo $i >>  count.txt; echo $(zcat $i | wc -l) / 4 | bc >> count.txt ;
done
## 7/ change file name
for f in *; do mv "$f" "${f/_2.fq.gz/_R2.fq.gz}"; done
for f in *; do mv "$f" "${f/_1.fq.gz/_R1.fq.gz}"; done
## 8/ create a tar.gz with all R1 and R2 files
go to the directory containing the files
tar zcvf 200lib.tar.gz a*
```

Barcodes informations and clustering

FROGS Options

min-abundance of 3 (min number of sequences) min-sample-presence 1 (min number of cluster) script lauch = /home/mariac/frogs/run_frogs_pipeline-18032020.sh (in Annexe1)

Barcodes, mk1, mk2

MK1 185 bp

F1- 5- TCHACHAAYCAYAAAGAYATYGGYACYCT
R1- 5'- ACYATRAARAARATYATYACRAADGC
Rev compl for frogs = GCHTTYGTRATRATYTTYTTYATRGT

MK2 285 bp

F1- 5'- TCHACHAAYCAYAAAGAYATYGGYACYCT
R2- 5'- CARAARCTYATRTTRTTYATTCGNGG
Rev compl for frogs = CCNCGAATRAAYAAYATRAGYTTYTG

FROGS for mk1

```
qsub -q bioinfo2.q@node20 -N frogsCL -b yes -V -cwd -pe ompi 8 '/home/mariac/frogs/
run_frogs_pipeline2.sh 120 280 TCHACHAAYCAYAARGAYATYGG  GCHTTYGTRATRATYTTYTTYATRGT
150 150 180 OUTPUT-MK1-min3-200lib 200lib.tar.gz'
```

FROGS for mk2

```
qsub -q bioinfo2.q@node20 -N frogsCL -b yes -V -cwd -pe ompi 8 '/home/mariac/frogs/
run_frogs_pipeline2.sh 200 300 TCHACHAAYCAYAARGAYATYGG CCNCGAATRAAYAAYATRAGYTTYTG
150 150 294 OUTPUT-MK2-min3-200lib 200lib.tar.gz'
```

Length distribution of the clusters

```r
setwd("C:/Users/mariac/Desktop/")
library("seqinr", lib.loc = "C:/Program Files/R/R-3.6.2/library")
mk1_200lib <- read.fasta(file = "04-filters-MK1-200lib.fasta")
length(mk1_200lib)  #how many fasta sequences
count(n1, 1)  #count number of nucleotides
n1 <- mk1_200lib[[2]]
GC(n1)  #GC content
lengths <- table(getLength(mk1_200lib))
par(mfrow = c(1, 1))
barplot(lengths, xlab = "mk1_200lib lengths", ylab = "Frequency")
```

Blastn OTU clusters

Blastn over DB-COI-02012020-v9.fasta with options -dust no -soft_masking false because there are in COI some species with a poly(T) that cannot be assigned (drop of % ID) script lauch with slurm (supermem or highmem required) = sample file in Annexe2 ### Built database :

```
makeblastdb -in DB-COI-02012020-v9.fasta -out DB-COI-02012020-v9.fasta -dbtype nucl
```

run blastn (2.8.1+) on DBCOIv9 (only fish)

```
for 04-filters-MK1-200lib-min3.fasta & 04-filters-MK2-200lib-min3.fasta
blastn -db /home/mariac/EDNA/DB-COI-02012020-v9.fasta -query 04-filters-MKx-200lib-min3.fasta
-out blast-ncbi-MKx-200lib-min3-DBCOIv9-dust.out -dust no -soft_masking false
-perc_identity 95 -num_alignments  25 -num_descriptions 4000 -num_threads 8
```

run blastn (2.8.1+) on NCBI local database (all taxa)

```
for 04-filters-MK1-200lib-min3.fasta & 04-filters-MK2-200lib-min3.fasta
blastn -db /data/projects/banks/nt_v4/nt -query 04-filters-MKx-200lib-min3.fasta
-out blast-ncbi-MKx-200lib-min3.out -dust no -soft_masking false -perc_identity 95
-num_alignments  25 -num_descriptions 4000 -num_threads 16
```

MEGAN assignation parameters

version 6.18.6

```
load taxonomyFile='D:\@EDNA-032020\ncbi-032020.tre' mapFile='D:\@EDNA-032020\ncbi-032020.map'
```

Import blastn output (MK1 and MK2 simultaneously) in megan details on options and files used in annexe (tre file, map file, synonym file) once the file is loaded (ctrl a) select all node and export to csv on excel combine this csv file and the 07-biom2tsv.tsv generated by frogs (abundance) using recherchev function then the abundance table could be reimported under MEGAN and results viewed at the sample level In excel maximum number of reads per taxa and per library over all negative control have been compute. This maximum value define the threshold value under a taxa must be ignored.(this value was 9 reads).

```
BLASTN on DBCOI:
Loading ncbi-032020.map: 1,788,632
Loading ncbi-032020.tre: 1,788,644

disable taxa=
Ictalurus punctatus, Schizodon intermedius, Pseudoplatystoma metaense,
Pseudoplatystoma magdaleniatum,Pseudoplatystoma punctifer,
Pseudoplatystoma reticulatum, Pseudoplatystoma corruscans,
Piaractus mesopotamicus, Serrasalmus cariba;
Total disabled taxa: 9

 preference/Accession Parsing/ : unselect First Word is accession & Set Accession Tags
 File : import BLASTN
 Taxonomy : Load Accession mapping file : ncbi-032020.map
 select Use accession
 synonyme file = ncbi-032020.synonyme.txt
 Use ID parsing : tax| taxonomy| >gi| TAXON_ID=
```

```
Blastn sur NCBI
min Score = 150
Max expected 0.01
Min % identity = 97
Top percent = 1
Min support Percent = 0
Min support = 1
Min complextity = 0
LCA Algorithm weighted
% to cover = 80.0
Read assignment Mode = readCount

Blastn sur DB COI (04062020)
min Score = 150
Max expected 0.01
Min % identity = 97
Top percent = 1 # The second score must be within 1% of the first in order to be retained.
  #(if Top is set to 1 and best score =100, if second score was 0.99 then is not retained)

Min support Percent = 0
Min support = 1
Min complextity = 0
LCA naive
% to cover = 50   # ok checked on 26062020
Read assignment Mode = readCount
```

Decontamination (substraction with microdecon package)

##Loading library

```
remotes::install_github("donaldtmcknight/microDecon")
library("microDecon", lib.loc = "~/R/win-library/3.6")
```

Input file format for microdecon

	Blank1	Blank2	Blank3	Pop1_Sample1	Pop1_Sample2	Pop2_Sample3	Taxa
OTU1	0	0	0	60	64	40	P_Actinobacteria
OTU2	200	220	180	660	520	480	P_Proteobacteria
OTU3	1000	800	1300	1440	1000	700	P_Proteobacteria
OTU4	50	30	70	70	48	35	P_Bacteroidetes
OTU5	0	0	0	2400	1900	2100	K_Bacteria
OTU6	25	10	30	30	20	15	K_Bacteria

Run Microdecon

Input file =2-Microdecon 13042020.xlsx sheet:“to microdecon”

```
otumat = read.table("clipboard", sep = "\t", header = T)  # table of OTU abundance
```

(must include not assigned and no hits) This file is very large ; an error could appear (Error during wrapup: cannot open the connection) meanning that the copy from excel is not ended.
Run microdecon

```
dim(otumat)
head(otumat)
result <- decon(otumat, numb.blanks = 3, runs = 2,
    taxa = T, numb.ind = c(3, 3, 3, 3, 4, 6, 1, 1,
        3, 16, 3, 2, 3, 3, 6, 6, 3, 3, 15, 3, 3, 3,
        6, 3, 3, 3, 4, 3, 3, 3, 3, 3, 3, 3, 3, 16,
        3), thresh = 1, prop.thresh = 5e-05, regression = 0,
    low.threshold = 40, up.threshold = 400)
```

prop.thresh = 0.000x if number of reads at a site for a given taxa are < 0.0x% set to zero (only OTU in blanks)

```
result$OTUs.removed
result$decon.table
result$reads.removed
write.csv(result$decon.table, file = "C:/Users/mariac/Desktop/to phyloseq 13042020.csv")
```

RUN PHYLOSEQ

setwd("C:/Users/mariac/Desktop")
setwd("C:/Users/mariac/Documents/Documents/ACEDRIC/Programmes/PIMELO-et-ECOBIO/E-DNA manipes/EDNA-200lib-032020/Article/Supplementary Data")

Loading libraries

```
library(devtools)
library(rlang)
library("BiocManager")
library("devtools")
library(maditr)
library("vctrs")
# library(tidyverse)
library(VennDiagram)
library("reshape2")
library(utf8)
library(phyloseq)
library(ggplot2)
library(RColorBrewer)
library(vegan)
library(dplyr)
library(psych)
library(grid)
library("ape")
```

```r
library(tidyquant)
library(microbiome)
library(gapminder)
library("lubridate", lib.loc = "~/R/win-library/3.6")
library("readr")
library(dendextend)
library(randomcoloR)
library("vegan", lib.loc = "~/R/win-library/3.6")
library("gplots", lib.loc = "~/R/win-library/3.6")
library("RcmdrMisc", lib.loc = "~/R/win-library/3.6")
library(pvclust, lib.loc = "C:/Program Files/R/R-4.0.3/library")
library(openxlsx, lib.loc = "C:/Program Files/R/R-4.0.3/library")
library(graphics)
set.seed(62)  # for reproduciblity
library(cowplot, lib.loc = "C:/Program Files/R/R-4.0.3/library")
```

Import taxmat (Taxonomy)

From excel, adding a variable with the Lowest available taxa (voir formule ds le fichier excel) setwd("C:/Users/mariac/Documents/Documents/ACEDRIC/Programmes/PIMELO-et-ECOBIO/E-DNA/manipes/EDNA-200lib-032020//Article/Supplementary Data/")

```r
setwd("C:/Users/mariac/Documents/Documents/ACEDRIC/Programmes/PIMELO-et-ECOBIO/E-DNA manipes/EDNA-200li
taxmat <- read.xlsx("SUPP DATA Tables and SFigures.xlsx",
    sheet = "taxmat", rowNames = T, colNames = TRUE)
taxmat2 <- as.matrix(taxmat)
TAX = tax_table(taxmat2)
ntaxa(TAX)  # number of taxas
```

```
## [1] 368
```

Import OTU

input sheet :STable5 abundance

```r
setwd("C:/Users/mariac/Documents/Documents/ACEDRIC/Programmes/PIMELO-et-ECOBIO/E-DNA manipes/EDNA-200li
otumat = read.xlsx("SUPP DATA Tables and SFigures.xlsx",
    sheet = "STable5 abundance", rowNames = T, colNames = TRUE,
    cols = c(1:159), rows = c(3:274))
otumat <- as.matrix(otumat)
OTU = otu_table(otumat, taxa_are_rows = TRUE)
```

Import metadata

```r
setwd("C:/Users/mariac/Documents/Documents/ACEDRIC/Programmes/PIMELO-et-ECOBIO/E-DNA manipes/EDNA-200li
metadata = read.xlsx("SUPP DATA Tables and SFigures.xlsx",
    sheet = "metadata", rowNames = F, colNames = TRUE)
metadata <- metadata %>% mutate(date = as.Date(date_of_collect,
    origin = "1899-12-30"))
```

```
metadata$date2 <- format(as.Date(metadata$date, format = "%Y/%m/%d"),
    "%d/%m/%Y")
row.names(metadata) <- metadata$SampleID
```

Format metadata as phyloseq object

```
metadata3 = sample_data(metadata)
```

check if sample names of Abondance and metadata are identical

```
sample_names(OTU) %in% sample_names(metadata3)
```

```
##   [1] TRUE TRUE TRUE TRUE TRUE TRUE TRUE TRUE TRUE TRUE TRUE TRUE TRUE TRUE TRUE
##  [16] TRUE TRUE TRUE TRUE TRUE TRUE TRUE TRUE TRUE TRUE TRUE TRUE TRUE TRUE TRUE
##  [31] TRUE TRUE TRUE TRUE TRUE TRUE TRUE TRUE TRUE TRUE TRUE TRUE TRUE TRUE TRUE
##  [46] TRUE TRUE TRUE TRUE TRUE TRUE TRUE TRUE TRUE TRUE TRUE TRUE TRUE TRUE TRUE
##  [61] TRUE TRUE TRUE TRUE TRUE TRUE TRUE TRUE TRUE TRUE TRUE TRUE TRUE TRUE TRUE
##  [76] TRUE TRUE TRUE TRUE TRUE TRUE TRUE TRUE TRUE TRUE TRUE TRUE TRUE TRUE TRUE
##  [91] TRUE TRUE TRUE TRUE TRUE TRUE TRUE TRUE TRUE TRUE TRUE TRUE TRUE TRUE TRUE
## [106] TRUE TRUE TRUE TRUE TRUE TRUE TRUE TRUE TRUE TRUE TRUE TRUE TRUE TRUE TRUE
## [121] TRUE TRUE TRUE TRUE TRUE TRUE TRUE TRUE TRUE TRUE TRUE TRUE TRUE TRUE TRUE
## [136] TRUE TRUE TRUE TRUE TRUE TRUE TRUE TRUE TRUE TRUE TRUE TRUE TRUE TRUE TRUE
## [151] TRUE TRUE TRUE TRUE TRUE TRUE TRUE TRUE
```

CompilationN taxmat, OTU & metadata

Merge in an phyloseq object OTU + TAXONOMY + metadata

```
physeq1 = phyloseq(OTU, TAX, metadata3)
physeq3 = physeq1
```

Filtering OTUs

Function : Applied frequency threshold (MAXSSS)

```
filterfun1 = function(x) {
    x[(x/sum(x)) < (1e-04)] <- 0  # set to 0 if prop <0.000x value from MAXSSS
    return(x)
}
```

Filtering phyloseq object : min abundance set to 0.01 %

```
GPfiltered <- transform_sample_counts(physeq3, fun = filterfun1)
# keep taxa only if > 0 read (over all sample)
physeq4_all_lib <- prune_taxa(taxa_sums(GPfiltered) >
    0, GPfiltered)
# Remove sample of low quality (too few reads)
physeq4 = subset_samples(physeq4_all_lib, SampleID !=
    "TUICHI3___c1e1" & SampleID != "CHAL___c1e4" &
```

```
    SampleID != "TUICHI3___c1e2" & SampleID != "TUICHI3___c1e3" &
    SampleID != "TUICHI6___c1e1")
# keep taxa only if > 0 read (over all sample)
physeq4 <- prune_taxa(taxa_sums(physeq4) > 0, physeq4)
```

Exclude some bad taxa in phyloseq object

```
badTaxa = c("Zoarces gillii", "Sebastiscus marmoratus",
    "Epinephelus marginatus", "Marcusenius monteiri")
# Epinephelus marginatus = Pimelodella gracilis ?
goodTaxa <- setdiff(taxa_names(physeq4), badTaxa)
physeq4 <- prune_taxa(goodTaxa, physeq4)
physeq4 <- prune_taxa(taxa_sums(physeq4) > 0, physeq4)
```

Count number of spec. genus over all sample including controls

```
x <- c("Order", "Family", "Genus", "Species", "lowest")
list <- c()
for (i in x) {
    list[i] <- dim(table(tax_table(physeq4)[, i]))
}
data.frame(t(sapply(list, c)), row.names = "physeq4")
```

Remove Controls

```
physeq4_rem_controls = subset_samples(physeq4, site !=
    "IRD" & site != "CTRL1" & site != "CTRL2" & site !=
    "NEG")
# keep taxa only if > 0 read (over all sample)
physeq4_rem_controls <- prune_taxa(taxa_sums(physeq4_rem_controls) >
    0, physeq4_rem_controls)
```

Count at different taxrank over all sample (excluding controls)

```
x <- c("Order", "Family", "Genus", "Species", "lowest")
list <- c()
for (i in x) {
    list[i] <- dim(table(tax_table(physeq4_rem_controls)[,
        i]))
}
data.frame(t(sapply(list, c)), row.names = "physeq4_rem_controls")
```

```
##                       Order Family Genus Species lowest
## physeq4_rem_controls    11     39   148     230    252
```

Merge samples at site level

```
physeq5wc = merge_samples(physeq4, "site")
physeq5 = merge_samples(physeq4_rem_controls, "site")
```

Merge samples at Biogeographic entities level

```
RANK = merge_samples(physeq4, "RANK")
```

Repair the merged values associated with each site after merge.

```
setwd("C:/Users/mariac/Documents/Documents/ACEDRIC/Programmes/PIMELO-et-ECOBIO/E-DNA manipes/EDNA-2001il
metadatasitelevel = read.xlsx("SUPP DATA Tables and SFigures.xlsx",
    sheet = "STable3 metad. site level", rowNames = F,
    colNames = TRUE, cols = c(1:29), rows = c(2:35))
row.names(metadatasitelevel) <- metadatasitelevel$Site.Name
metadatasitelevel2 = sample_data(metadatasitelevel)
sample_names(physeq5@otu_table) %in% sample_names(metadatasitelevel2)
```

```
##  [1] TRUE TRUE TRUE TRUE TRUE TRUE TRUE TRUE TRUE TRUE TRUE TRUE TRUE TRUE TRUE
## [16] TRUE TRUE TRUE TRUE TRUE TRUE TRUE TRUE TRUE TRUE TRUE TRUE TRUE TRUE TRUE
## [31] TRUE TRUE TRUE
```

```
physeq5 = phyloseq(physeq5@otu_table, TAX, metadatasitelevel2)
# head(physeq5@sam_data)
```

Transform to %

Transform to proportion

```
physeq5p = transform_sample_counts(physeq5, function(x) x/sum(x))
```

Count number of reads per lib

```
par(mar = c(7, 6, 4.1, 2.1))
barplot(log(sample_sums(physeq5)), main = " Read Counts",
    xlab = "", border = "blue", col = "green", las = 2,
    cex.names = 0.8)
```

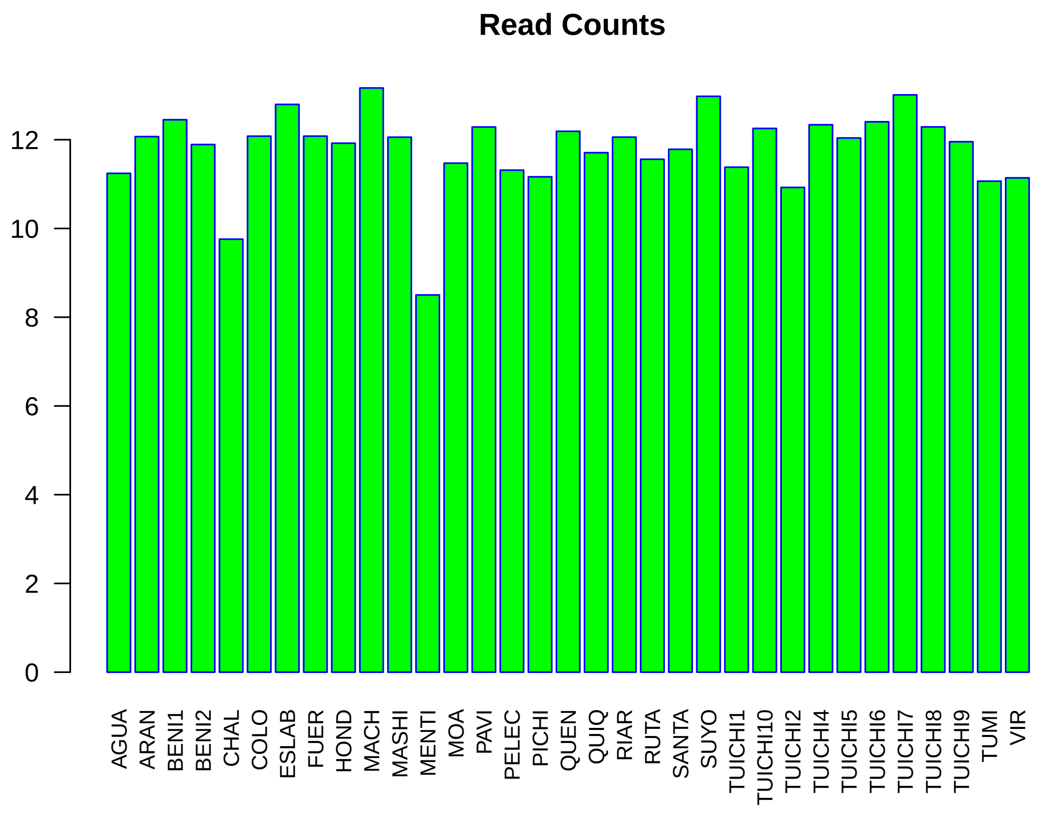

RAREFACTION CURVES per site

physeq4_all_lib

```
# at site level : following lib must be excluded
# because of low number of reads -> ggrare can't
# run
x = subset_samples(physeq4, SampleID != "CHAL___c1e4" &
    SampleID != "TUICHI3___c1e2" & SampleID != "TUICHI3___c1e3" &
    SampleID != "TUICHI3___c1e1" & SampleID != "TUICHI6___c1e1")

y = merge_samples(x, "site")
sample_data(y)$site <- factor(sample_names(y))
sample_data(y)$site <- factor(sample_data(y)$site)


source("https://raw.githubusercontent.com/mahendra-mariadassou/phyloseq-extended/master/load-extra-func

p <- ggrare(x, step = 50, color = "site", plot = T,
    parallel = T, se = F, # label=)

p <- p + facet_wrap(~site) + xlim(0, 50000) + theme(axis.text.x = element_text(angle = 45)) +
    # geom_vline(xintercept = min(sample_sums(x)),color
```

```
# = 'gray60')+
theme(legend.position = "none")

plot(p)
```

RAREFACTION CURVES at lib level

```
# at site level
x = subset_samples(physeq5)

source("https://raw.githubusercontent.com/mahendra-mariadassou/phyloseq-extended/master/load-extra-func

p <- ggrare(x, step = 50, color = "Site.Name", plot = T,
    parallel = T, se = F, # label=)

p <- p + facet_wrap(~Site.Name) + xlim(0, 10000) +
    theme(axis.text.x = element_text(angle = 45))
# geom_vline(xintercept = min(sample_sums(x)),color
# = 'gray60')+
theme(legend.position = "none")

plot(p)
```

Count in how many site taxa are present

```
asv_df <- (otu_table(physeq5))
# View(colSums(asv_df != 0))
SFS <- colSums(asv_df != 0)
write.csv(SFS, "C:/Users/mariac/Documents/Documents/ACEDRIC/Programmes/PIMELO-et-ECOBIO/E-DNA manipes/E
par(mfrow = c(2, 1))
hist(SFS, breaks = c(0:33), col = "red", freq = T,
    main = "Species Frequency Spectrum", ylab = "Number of Species",
    xlab = "Number of sites where each species is present")

hist(SFS, breaks = c(0, 4, 10, 20, 32), col = "red",
    freq = T, main = "Species Frequency Spectrum",
    ylab = "Number of Species", xlab = "Number of sites where each species is present")
```

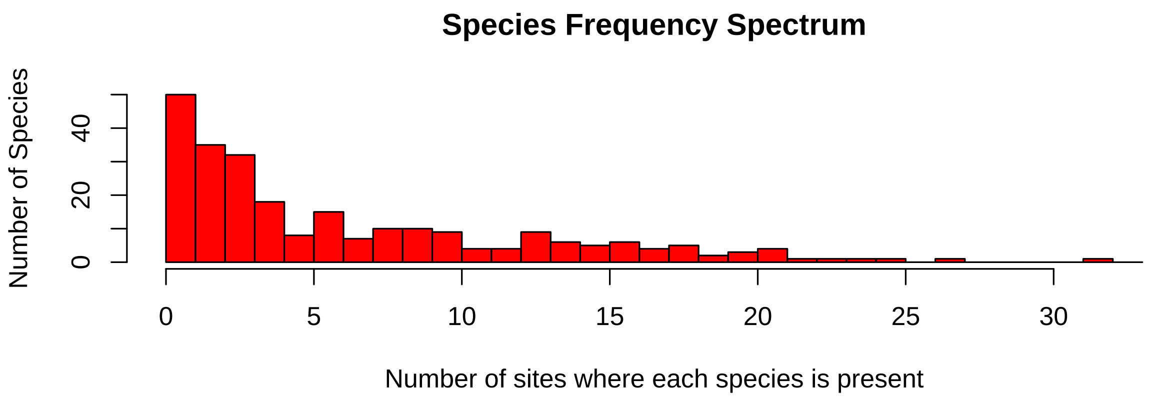

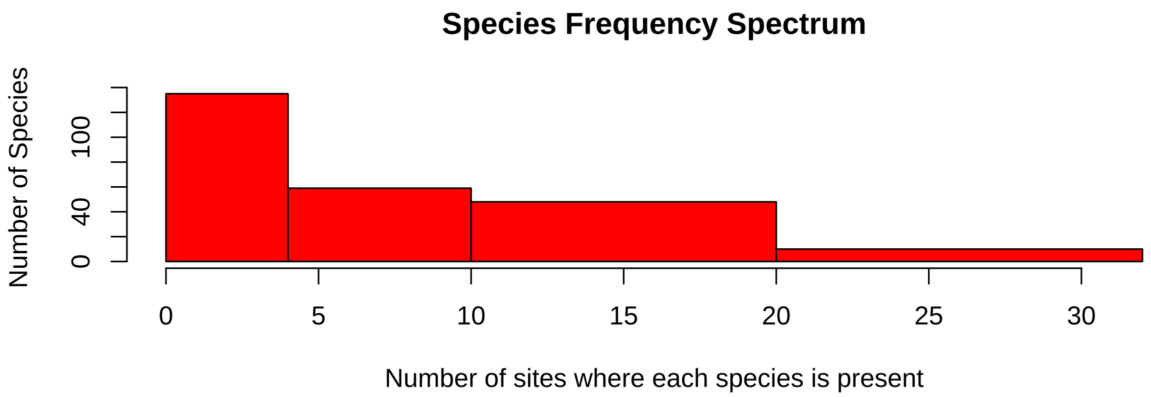


```
library(pastecs)
stat.desc(SFS)
```

```
##       nbr.val     nbr.null      nbr.na          min          max        range
##   252.0000000    0.0000000   0.0000000    1.0000000   32.0000000   31.0000000
##           sum       median        mean      SE.mean CI.mean.0.95          var
## 1684.0000000    4.0000000   6.6825397    0.3934703    0.7749241   39.0143553
##       std.dev     coef.var
##     6.2461472    0.9346966
```

ALPHA diversity

```
library(randomcoloR)
n <- 33
palette <- distinctColorPalette(n)
# pie(rep(1,n), col=sample(palette, n))
```

NMDS on Libraries

```r
set.seed(62)
par(mfrow = c(1, 1))
sampledf <- data.frame(sample_data(physeq4_rem_controls))
sampledf$site2 <- as.factor(sampledf$site)

ord <- ordinate(physeq4_rem_controls, "NMDS", distance = "jaccard",
    binary = TRUE, k = 2, maxit = 1500, trymax = 10000,
    wascores = TRUE, verbose = FALSE)

p0 = plot_ordination(physeq4_rem_controls, ord, type = "samples",
    color = "site", title = "NMDS at Library level ",
    shape = "site")
p0 + scale_shape_manual(values = rep(1:9, 4))
```

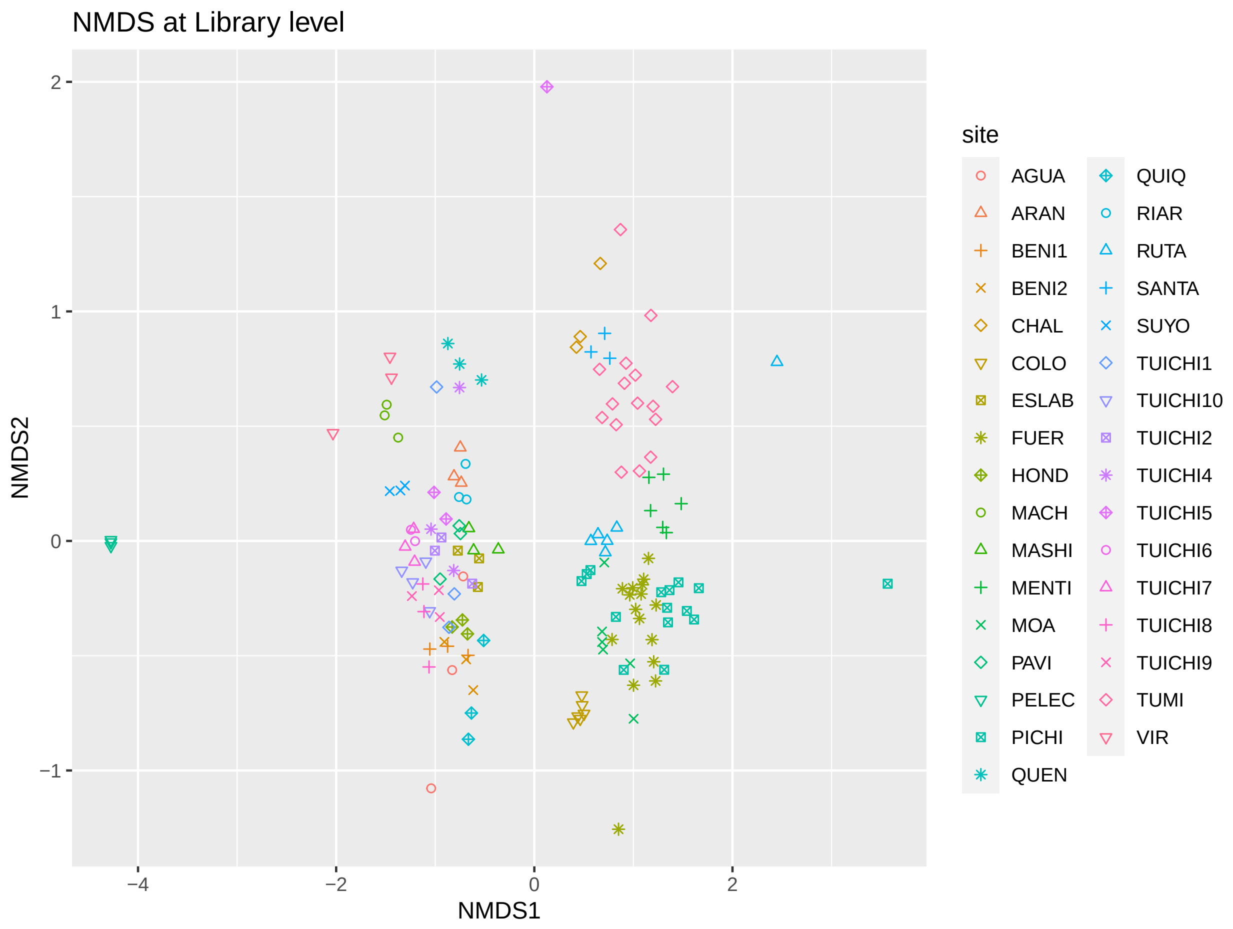


```r
theme(panel.background = element_rect(fill = "white",
    colour = "grey50"))


with(sampledf, plot(ord$points, pch = 16, type = "n",
    col = palette[site2]))
```

```r
with(sampledf, ordispider(ord, groups = sampledf$site2,
    label = T, col = palette[site2], cex = 0.5, lwd = 3))

with(sampledf, points(ord, display = "sites", pch = 16,
    col = palette[site2], bg = palette[site2]))
```

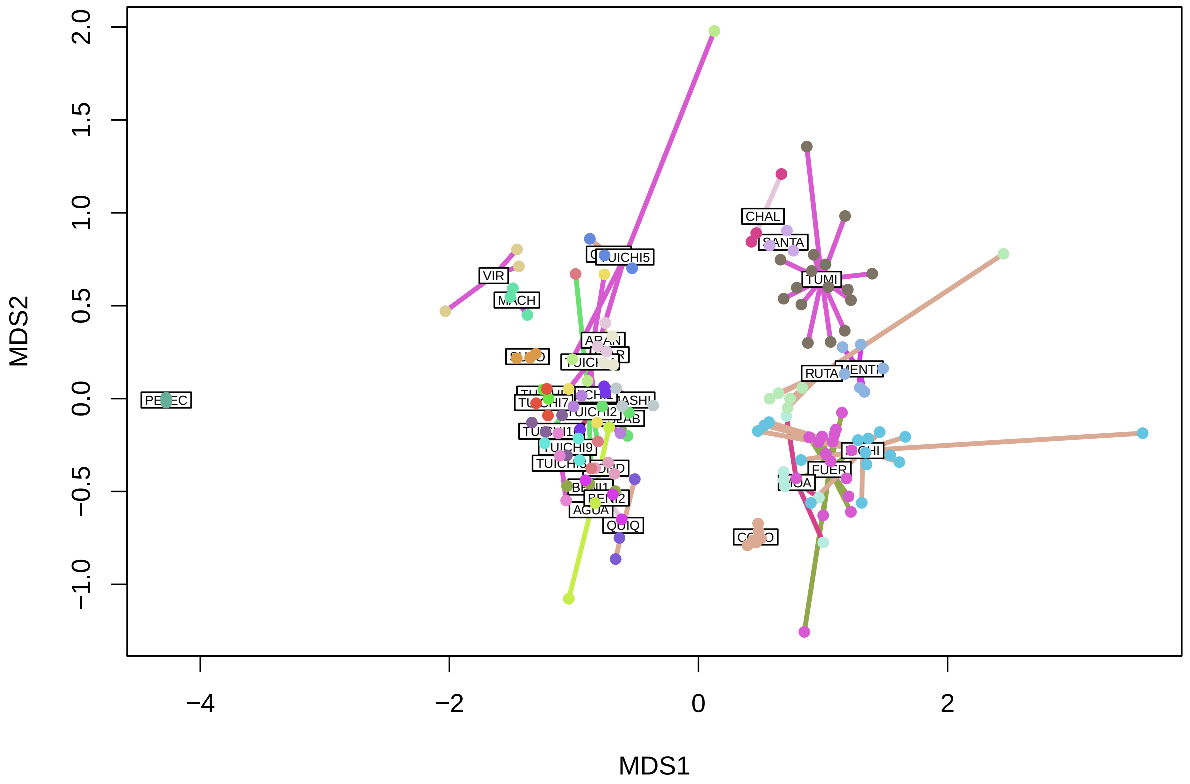

Plot richness per LIB

fill="Order"

```r
pr <- plot_richness(physeq4, x = "site", color = "site",
    measures = c("Observed", "Shannon", "Simpson"),
    sortby = "Observed") + geom_boxplot(alpha = 0.1) +
    theme(legend.position = "none") + # theme(axis.title.x=element_blank(),
# axis.text.x=element_blank(),
# axis.ticks.x=element_blank())+
labs(x = "Library * Site") + theme(plot.margin = unit(c(0,
    0, 0, 0), "cm"), panel.background = element_rect(fill = "white",
    colour = "grey50"))
pr + theme(axis.text = element_text(size = 6), axis.title = element_text(size = 10,
    face = "bold"))
```

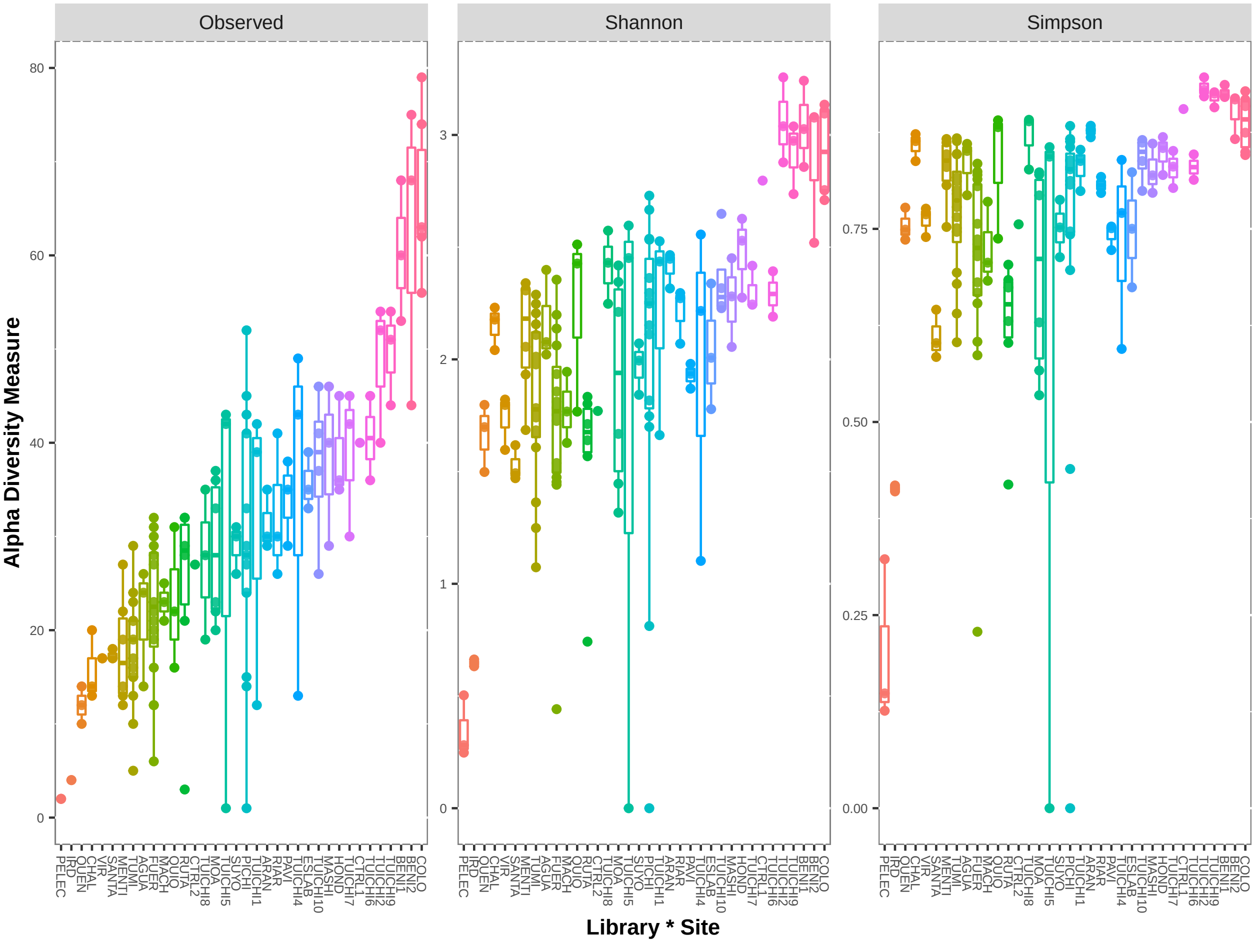

Plot richness per SITE

(merge data lib per site = physeq5)

```
pr2 <- plot_richness(physeq5, x = "Site.Name", color = "Site.Name",
    measures = c("Observed", "Shannon", "Simpson"),
    sortby = "Observed") + geom_boxplot(alpha = 0.1) +
    theme(legend.position = "none") + labs(x = "SITES") +
    theme(plot.margin = unit(c(0, 0, 0, 0), "cm"),
        panel.background = element_rect(fill = "white",
            colour = "grey50"))

# pr$data #Extract data
pr3 <- pr2 + theme(panel.grid.major = element_blank(),
    panel.grid.minor = element_blank(), panel.background = element_blank(),
    axis.line = element_line(colour = "black")) + scale_x_discrete(limits = c("RUTA",
    "CHAL", "SANTA", "COLO", "MOA", "MENTI", "TUMI",
    "FUER", "PICHI", "PELEC", "VIR", "MACH", "SUYO",
    "AGUA", "ESLAB", "PAVI", "RIAR", "ARAN", "MASHI",
    "BENI1", "BENI2", "TUICHI6", "TUICHI7", "TUICHI4",
    "TUICHI5", "TUICHI2", "TUICHI10", "TUICHI9", "QUEN",
    "QUIQ", "TUICHI8", "HOND", "TUICHI1"))
plot_grid(pr2, pr3, ncol = 1, align = "hv", scale = 1)
```

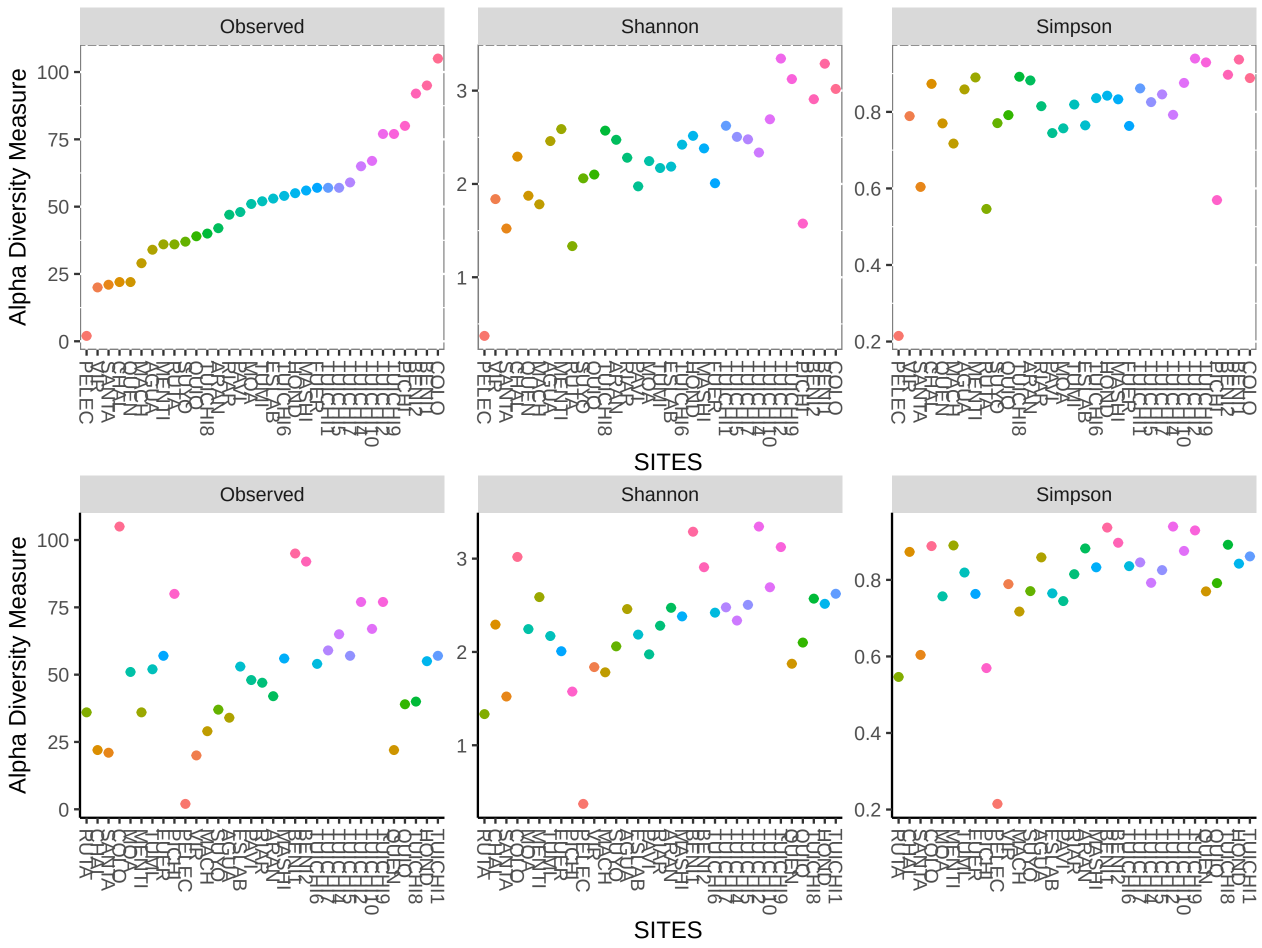

Barplot taxo diversity per site

Comparison of Family assemblage with sites. We must use merge samples that are from the same site = physeq5p for proportion Could be at Order, Genus and lowest level (following is at Order only)

```r
g1 <- plot_bar(physeq5p, x = "Site.Name", fill = "Order") +
    theme(legend.key.size = unit(1, "cm"), axis.text.x = element_text(size = 10,
        vjust = 0), legend.text = element_text(size = 10)) +
    theme(panel.grid.major = element_blank(), panel.grid.minor = element_blank(),
        panel.background = element_blank(), axis.line = element_line(colour = "black"))
theme(legend.position = "none")
```

```
## List of 1
##  $ legend.position: chr "none"
##  - attr(*, "class")= chr [1:2] "theme" "gg"
##  - attr(*, "complete")= logi FALSE
##  - attr(*, "validate")= logi TRUE
```

```
g1
```

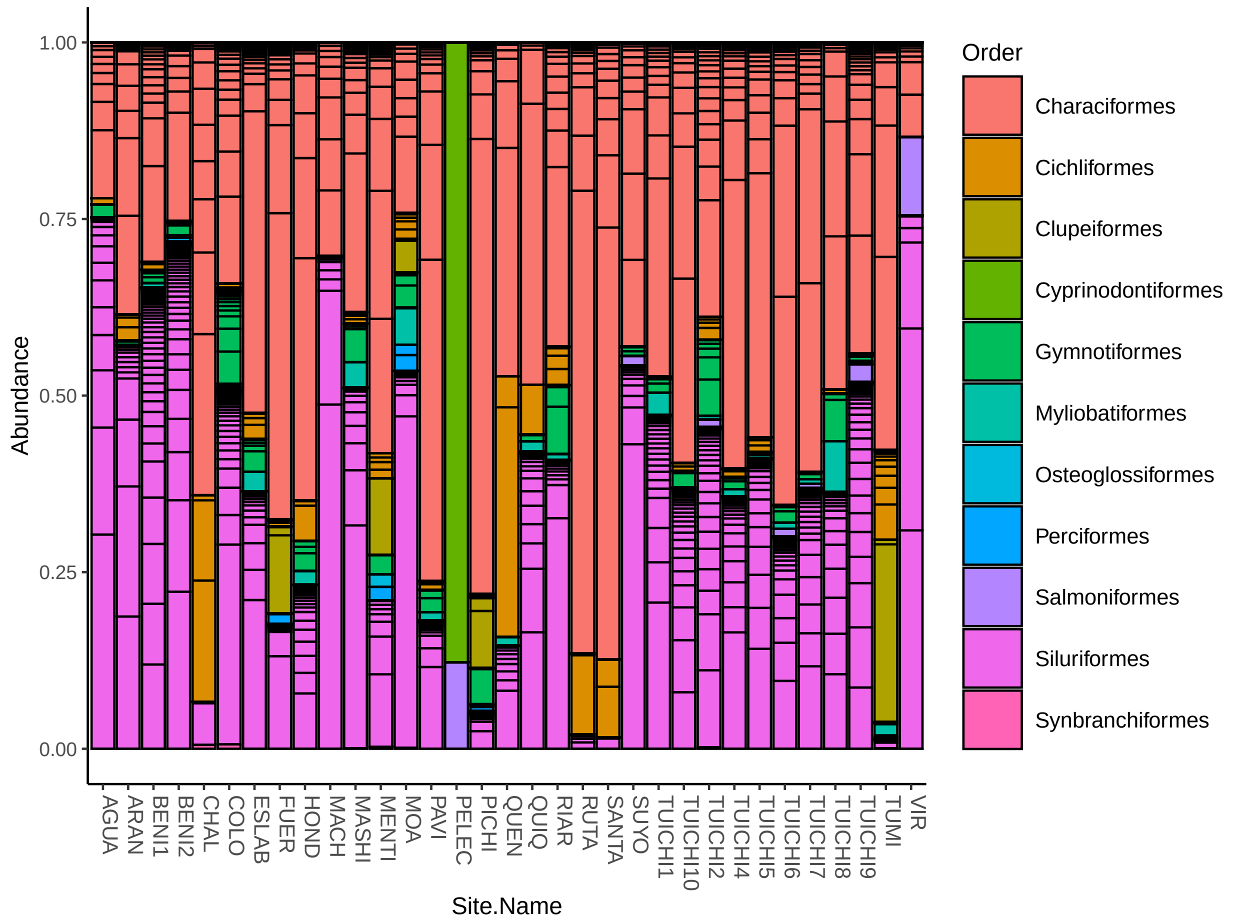

Barplot taxo diversity per site (facet View)

```
pb <- plot_bar(physeq5p, x = "Site.Name", fill = "Order",
    facet_grid = "Order~.") + theme(strip.text.x = element_text(size = 3,
    color = "red", face = "bold.italic"))
pb2 <- pb + scale_x_discrete(limits = c("RUTA", "CHAL",
    "SANTA", "COLO", "MOA", "MENTI", "TUMI", "FUER",
    "PICHI", "PELEC", "VIR", "MACH", "SUYO", "AGUA",
    "ESLAB", "PAVI", "RIAR", "ARAN", "MASHI", "BENI1",
    "BENI2", "TUICHI6", "TUICHI7", "TUICHI4", "TUICHI5",
    "TUICHI2", "TUICHI10", "TUICHI9", "QUEN", "QUIQ",
    "TUICHI8", "HOND", "TUICHI1")) + theme(panel.grid.major = element_blank(),
    panel.grid.minor = element_blank(), panel.background = element_blank(),
    axis.line = element_line(colour = "black")) + theme(legend.position = "none")
# plot_grid(pr3,pb2, ncol=1, align='hv',scale=1)
pb2 + theme(strip.text.x = element_text(size = 3, color = "red",
    face = "bold.italic"))
```

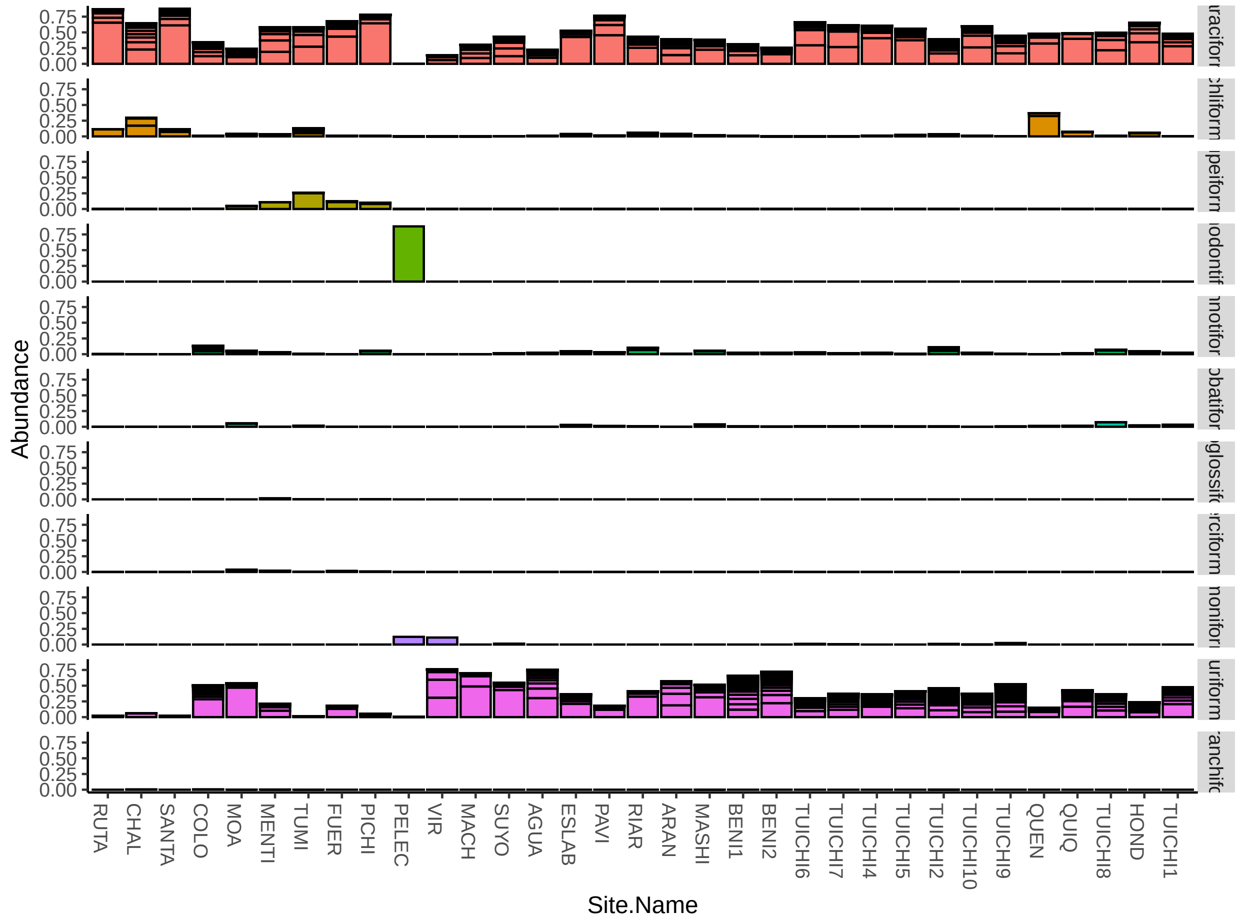

Compare RIVER and LAKE

EXCLUDED : controls and Alien : CTRL, IRD and TUICHI3

```
phyloseqRiver <- subset_samples(physeq5, ECOTYPE ==
    "RIVER")
phyloseqRiver <- prune_taxa(taxa_sums(phyloseqRiver) >
    0, phyloseqRiver)
phyloseqLake <- subset_samples(physeq5, ECOTYPE ==
    "LAKE")
phyloseqLake <- prune_taxa(taxa_sums(phyloseqLake) >
    0, phyloseqLake)

x <- c("Order", "Family", "Genus", "Species", "lowest")

list <- c()
for (i in x) {
    list[i] <- dim(table(tax_table(phyloseqRiver)[,
        i]))
}
data.frame(t(sapply(list, c)), row.names = "RIVER")
```

```
##       Order Family Genus Species lowest
```

```
## RIVER     9     31   103     152    172
```

```r
for (i in x) {
    list[i] <- dim(table(tax_table(phyloseqLake)[,
        i]))
}
data.frame(t(sapply(list, c)), row.names = "LAKE")
```

```
##      Order Family Genus Species lowest
## LAKE     9     33   105     143    153
```

```r
for (i in x) {
    list[i] <- dim(table(tax_table(physeq5)[, i]))
}
data.frame(t(sapply(list, c)), row.names = "ALL")
```

```
##     Order Family Genus Species lowest
## ALL    11     39   148     230    252
```

Venn Diagram

```r
library("openxlsx")
library(ggvenn)

data <- read.xlsx("C:/Users/mariac/Documents/Documents/ACEDRIC/Programmes/PIMELO-et-ECOBIO/E-DNA manipe
    sheet = "VennR", rowNames = F, colNames = TRUE,
    na.strings = "NA")

Lakes <- data[!is.na(data$Lakes), "Species"]
Lakes_Tuichi <- data[!is.na(data$Lakes_Tuichi), "Species"]
Lakes_Beni <- data[!is.na(data$Lakes_Beni), "Species"]

Rivers_Alto_Tuichi <- data[!is.na(data$Rivers_Alto_Tuichi),
    "Species"]
Rivers_Rank_3 <- data[!is.na(data$Rivers_Rank_3), "Species"]
Rivers_Rank_1_and_2 <- data[!is.na(data$Rivers_Rank_1_and_2),
    "Species"]

# plot with all sites
input <- list(Lakes = Lakes, Rivers_Alto_Tuichi = Rivers_Alto_Tuichi,
    Rivers_Rank_3 = Rivers_Rank_3, Rivers_Rank_1_and_2 = Rivers_Rank_1_and_2)
ggvenn(input, fill_color = c("#0073C2FF", "#CD534CFF",
    "#339900", "#EFC000FF"), stroke_size = 1, set_name_size = 3.5)
```

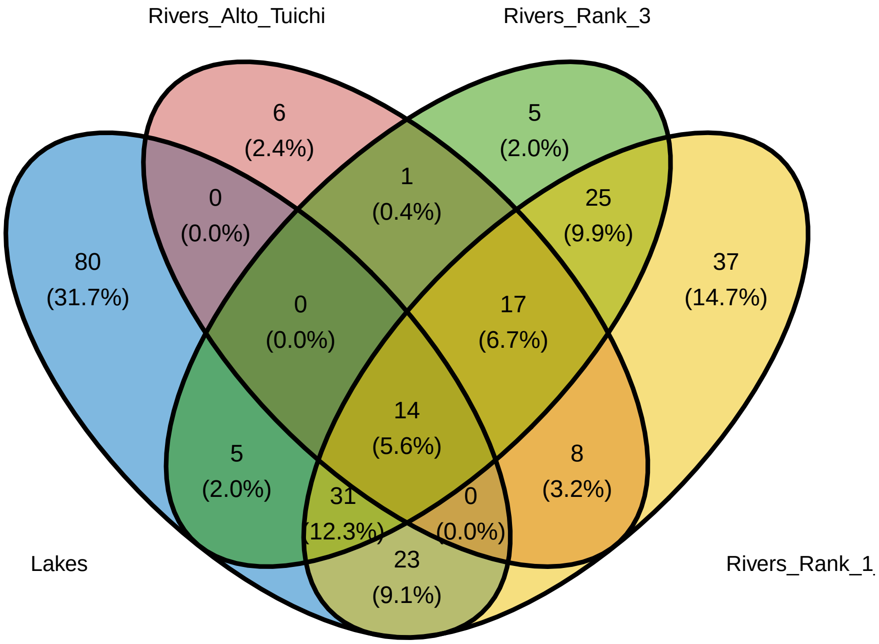


```r
# plot avec lacs Tuichi seulement
input <- list(Lakes_Tuichi = Lakes_Tuichi, Rivers_Alto_Tuichi = Rivers_Alto_Tuichi,
    Rivers_Rank_3 = Rivers_Rank_3, Rivers_Rank_1_and_2 = Rivers_Rank_1_and_2)
# pdf(file = 'out.pdf', width = 9, height = 9,
# pointsize = 10)
toto <- ggvenn(input, fill_color = c("#0073C2FF", "#CD534CFF",
    "#339900", "#EFC000FF"), stroke_size = 1, set_name_size = 3.5)
toto
```

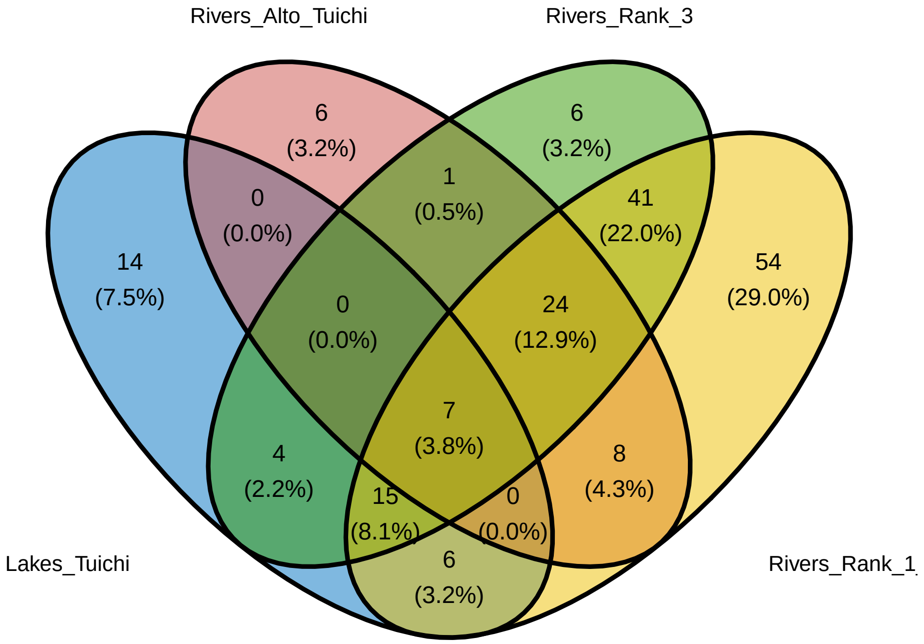


```
# dev.off()

# Lakes tuichi / lakes Beni
input <- list(Lakes_Tuichi = Lakes_Tuichi, Lakes_Beni = Lakes_Beni)
ggvenn(input, fill_color = c("#0073C2FF", "#61A5A7"),
    stroke_size = 1, set_name_size = 3.5)
```

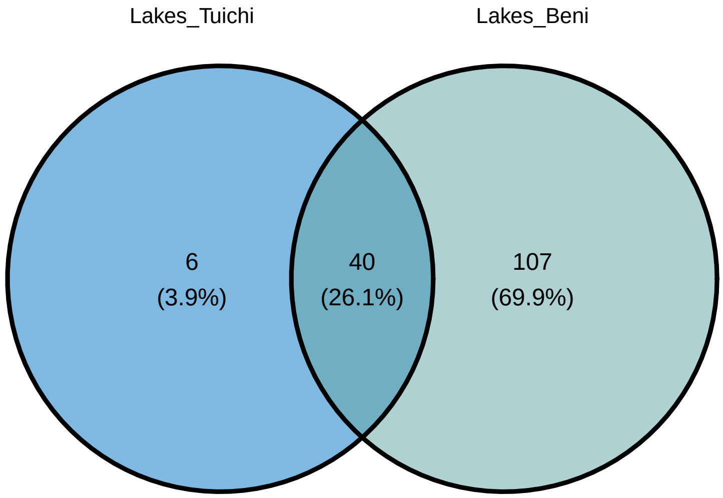

Retreived specific Order to Rivers or lakes

```
LAKE = subset_samples(physeq4_rem_controls, RANK !=
    "1" & RANK != "2" & RANK != "3")
RIVER = subset_samples(physeq4_rem_controls, RANK !=
    "L")

LAKE2 <- prune_taxa(taxa_sums(LAKE) > 0, LAKE)
RIVER2 <- prune_taxa(taxa_sums(RIVER) > 0, RIVER)

# sample_names(RIVER2)

a <- table(tax_table(physeq4_rem_controls)[, "Order"])
b <- table(tax_table(LAKE2)[, "Order"])
c <- table(tax_table(RIVER2)[, "Order"])
d <- left_join(as.data.frame(a), as.data.frame(b),
    by = "Var1")
e <- left_join(as.data.frame(d), as.data.frame(c),
    by = "Var1")
names(e)[1] <- "Order"
names(e)[2] <- "ALL"
names(e)[3] <- "Lakes"
```

```r
names(e)[4] <- "Rivers"
e
```

```
##                  Order ALL Lakes Rivers
## 1       Characiformes  87    61     56
## 2        Cichliformes  15    13      6
## 3        Clupeiformes   4     4     NA
## 4  Cyprinodontiformes   1    NA      1
## 5       Gymnotiformes  23    20     13
## 6     Myliobatiformes   3     2      3
## 7   Osteoglossiformes   1     1     NA
## 8         Perciformes   2     2      1
## 9       Salmoniformes   1    NA      1
## 10       Siluriformes 114    49     90
## 11   Synbranchiformes   1     1      1
```

ANOVA on Shannon index versus Site or ECOTYE

```r
alpha.diversity <- estimate_richness(physeq5, measures = c("Observed",
    "Chao1", "Shannon", "Simpson", "ACE"))
alpha.diversity
```

```
##           Observed Chao1 se.chao1 ACE    se.ACE   Shannon   Simpson
## AGUA            34    34        0  34 1.6538725 2.4597146 0.8587765
## ARAN            42    42        0  42 2.2677868 2.4731918 0.8821393
## BENI1           95    95        0  95 2.1764288 3.2886087 0.9366403
## BENI2           92    92        0  92 2.8494851 2.9082118 0.8969530
## CHAL            22    22        0  22 2.0889319 2.2929680 0.8731425
## COLO           105   105        0 105 3.0079260 3.0182452 0.8883411
## ESLAB           53    53        0  53 0.9905211 2.1858096 0.7647802
## FUER            57    57        0  57 2.8715177 2.0077740 0.7633275
## HOND            55    55        0  55 1.3882625 2.5157905 0.8423302
## MACH            29    29        0 NaN       NaN 1.7817033 0.7172509
## MASHI           56    56        0  56 1.9272482 2.3812430 0.8328384
## MENTI           36    36        0  36 2.5980762 2.5879566 0.8899589
## MOA             51    51        0  51 1.9199673 2.2450570 0.7570260
## PAVI            48    48        0  48 1.3844373 1.9742757 0.7446635
## PELEC            2     2        0 NaN       NaN 0.3715692 0.2147518
## PICHI           80    80        0  80 4.1892720 1.5754794 0.5695735
## QUEN            22    22        0  22 0.9770084 1.8735246 0.7699695
## QUIQ            39    39        0  39 2.0878157 2.1006758 0.7915598
## RIAR            47    47        0  47 2.1137845 2.2810368 0.8149185
## RUTA            36    36        0  36 2.5980762 1.3338601 0.5463995
## SANTA           21    21        0  21 0.9759001 1.5223219 0.6039812
## SUYO            37    37        0  37 0.9863939 2.0601930 0.7706451
## TUICHI1         57    57        0  57 2.1357443 2.6235041 0.8613847
## TUICHI10        67    67        0  67 2.3372391 2.6931373 0.8756776
## TUICHI2         77    77        0  77 2.6774673 3.3438138 0.9392959
## TUICHI4         65    65        0  65 1.9374845 2.3365724 0.7922929
## TUICHI5         57    57        0  57 1.6858545 2.5046154 0.8255428
## TUICHI6         54    54        0 NaN       NaN 2.4210798 0.8359079
```

```
## TUICHI7        59    59        0  59 1.6874411 2.4781771 0.8457133
## TUICHI8        40    40        0  40 2.0916501 2.5713381 0.8917864
## TUICHI9        77    77        0  77 2.8192290 3.1240847 0.9292512
## TUMI           52    52        0  52 2.4612380 2.1707771 0.8191763
## VIR            20    20        0  20 0.9746794 1.8378191 0.7889427
```

```
data <- cbind(physeq5@sam_data, alpha.diversity)
summary(aov(Shannon ~ ECOTYPE, data))  # NS
```

```
##             Df Sum Sq Mean Sq F value Pr(>F)
## ECOTYPE      1  0.492  0.4917    1.45  0.238
## Residuals   31 10.513  0.3391
```

```
summary(aov(Simpson ~ ECOTYPE, data))  # NS
```

```
##             Df Sum Sq Mean Sq F value Pr(>F)
## ECOTYPE      1 0.0265 0.02654   1.355  0.253
## Residuals   31 0.6071 0.01958
```

```
summary(aov(Observed ~ ECOTYPE, data))  # NS
```

```
##             Df Sum Sq Mean Sq F value Pr(>F)
## ECOTYPE      1      0     0.1       0   0.99
## Residuals   31  17139   552.9
```

```
summary(aov(Observed ~ ClusterGroups, data))  # NS
```

```
##               Df Sum Sq Mean Sq F value Pr(>F)
## ClusterGroups  1   1261  1261.2   2.462  0.127
## Residuals     31  15878   512.2
```

```
summary(aov(Observed ~ ALTITUDE, data))  # S
```

```
##             Df Sum Sq Mean Sq F value  Pr(>F)
## ALTITUDE     1   3786    3786   8.789 0.00578 **
## Residuals   31  13353     431
## ---
## Signif. codes:  0 '***' 0.001 '**' 0.01 '*' 0.05 '.' 0.1 ' ' 1
```

```
boxplot(data$Observed ~ data$River.Rank)
```

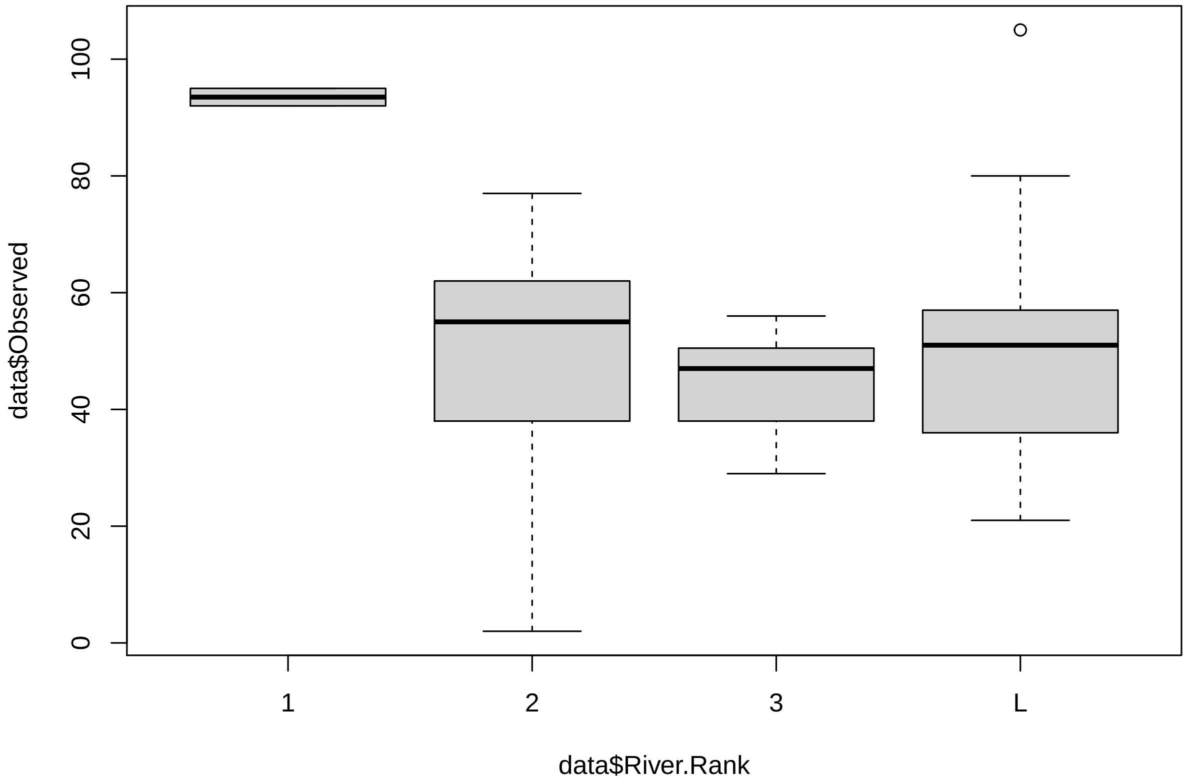

MAXSSS

```r
# Loading libraries
library(ROCR)
library("openxlsx")
```

Import DATA

Input file with sanger identification (must be 0 or 1) and NGS results (frequencies) raw line > 0 Sanger and NGS data in two column one for NGS one for sanger input file : SUPP DATA Tables and SFigures.xlsx sheet : "STable6 MAXSSS" only column L:M, row 2:195

```r
setwd("C:/Users/mariac/Documents/Documents/ACEDRIC/Programmes/PIMELO-et-ECOBIO/E-DNA manipes/EDNA-2001il
Binary <- read.xlsx("SUPP DATA Tables and SFigures.xlsx",
    sheet = "STable6 MAXSSS", rowNames = F, colNames = TRUE,
    rows = c(2:195), cols = c(4:5))
colnames(Binary)[1] = "NGS"
colnames(Binary)[2] = "sanger"
# str(Binary)
```

Convert data in ROCR format

```
pred <- prediction(as.numeric(Binary$NGS), as.numeric(Binary$sanger))
```

Analysis of performance on tpr and fpr then on specificity

```
perf1 <- performance(pred, "tpr", "fpr")
cutoffs1 <- data.frame(cut = perf1@alpha.values[[1]],
    fpr = perf1@x.values[[1]], tpr = perf1@y.values[[1]])
perf2 <- performance(pred, "sens", "spec")
cutoffs2 <- data.frame(cut = perf2@alpha.values[[1]],
    specificity = perf2@x.values[[1]], sensitivity = perf2@y.values[[1]],
    SSS = (perf2@x.values[[1]] + perf2@y.values[[1]]))
all <- merge(cutoffs1, cutoffs2)
```

Identify the 'optimal' cutoff

(MAXSSS)that yields the highest sensitivity and specificity: positif if test >= to these threshold value

```
CP <- perf2@alpha.values[[1]][which.max(perf2@x.values[[1]] +
    perf2@y.values[[1]])]
CP
```

```
## [1] 0.04017268
```

ROC curves

```
roc <- performance(pred, "tpr", "fpr")
plot(roc, colorize = T)
abline(a = 0, b = 1)
```

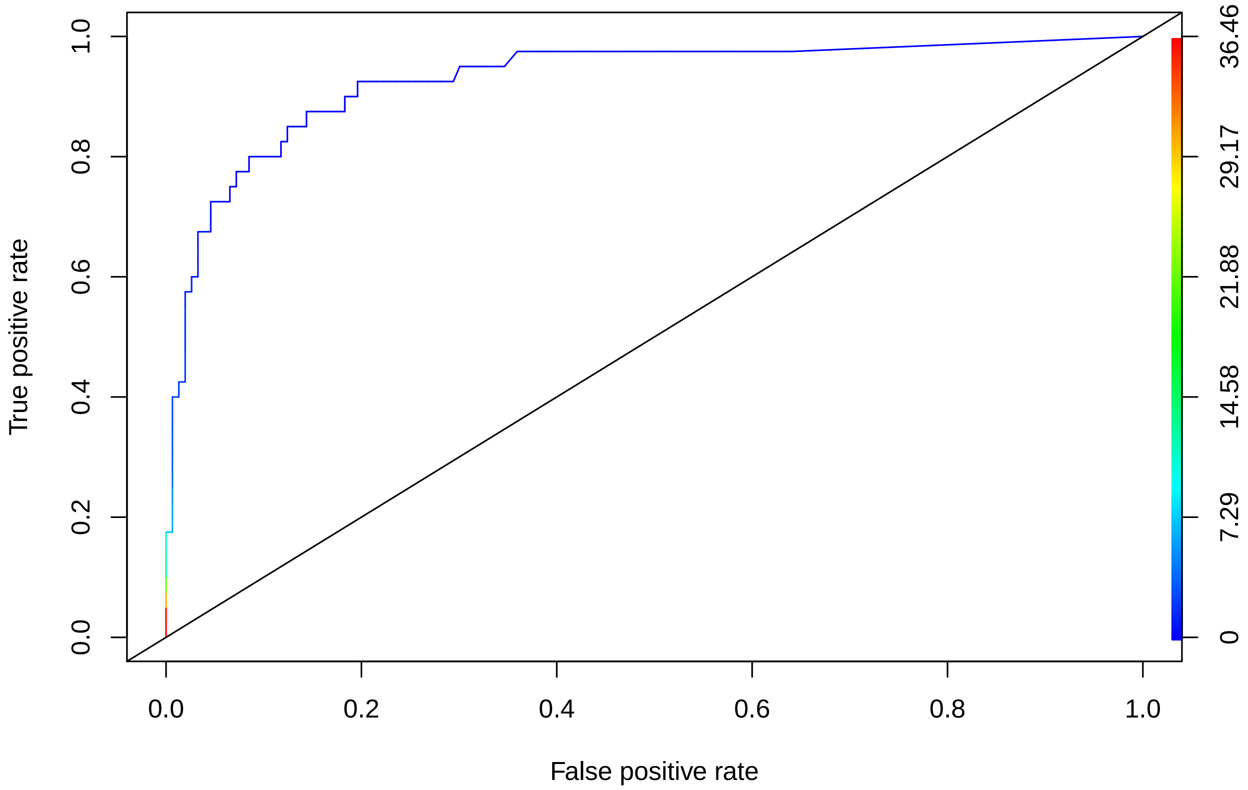

Expected richness

```
# remotes::install_github('microbiome/microbiome')
library("microbiome")
library(vegan)
```

Extract OTU to dataframe

```
temp4 <- t(as.data.frame(physeq4_rem_controls@otu_table))
```

Extract sample data to a dataframe

```
meta3 <- meta(physeq4_rem_controls)
```

Richness & expected richness on the desired hierarchical level

specpool (pool) permet de merger les samples d un meme site en utilisant rowname de meta2 et temp2

```
res <- with(meta3, specpool(temp4, pool = site, smallsample = TRUE))
# res
library(gridExtra)
library(magick)

png(filename = "output.png", width = 1000, height = 800,
    bg = "white")
grid.table(ceiling(res))
dev.off()
```

```
## pdf
##   2
```

```
img <- magick::image_read("output.png")
plot(img)
```

	Species	chao	chao.se	jack1	jack1.se	jack2	boot	boot.se	n
AGUA	34	39	4	43	7	45	39	5	3
ARAN	42	52	8	51	7	54	47	4	3
BENI1	95	111	7	120	18	127	108	11	3
BENI2	92	108	8	114	19	122	103	11	3
CHAL	22	26	4	27	5	29	25	3	3
COLO	105	121	9	124	9	132	114	6	6
ESLAB	53	59	4	65	9	67	59	6	3
FUER	57	62	4	66	4	66	62	3	16
HOND	55	66	7	68	10	73	62	6	3
MACH	29	31	2	33	3	33	31	2	3
MASHI	56	67	7	70	11	74	63	6	3
MENTI	36	39	3	42	5	43	40	4	6
MOA	51	56	4	59	5	61	55	4	6
PAVI	48	55	5	58	8	61	53	5	3
PELEC	2	2	0	2	0	2	2	0	3
PICHI	80	82	2	86	3	79	85	4	15
QUEN	22	28	5	30	6	32	26	4	3
QUIQ	39	46	5	51	9	54	45	6	3
RIAR	47	55	5	58	10	61	53	6	3
RUTA	36	36	0	36	0	33	37	2	6
SANTA	21	23	3	24	2	25	23	2	3
SUYO	37	40	3	43	4	44	40	3	3
TUICHI1	57	72	8	77	16	83	67	10	3
TUICHI10	67	86	10	87	12	95	77	7	4
TUICHI2	77	88	6	97	15	102	87	9	3
TUICHI4	65	83	9	88	19	96	76	12	3
TUICHI5	57	68	6	77	17	82	67	12	3
TUICHI6	54	61	4	68	14	68	61	8	2
TUICHI7	59	70	6	74	11	79	67	7	3
TUICHI8	40	45	4	49	9	51	45	6	3
TUICHI9	77	100	11	99	16	108	88	8	3
TUMI	52	54	3	58	3	56	56	3	16
VIR	20	21	1	22	1	22	21	1	3

Expected Richness over all Sites ; number of species per "pool" (eg site or ECOTYPE ...)

```
with(meta3, specpool(temp4, smallsample = TRUE))
```

```
##      Species     chao   chao.se    jack1 jack1.se    jack2     boot  boot.se   n
## All      252 264.0188 6.745757 273.8523  5.79872 275.9588 263.5849 4.445683 149
```

Observed Richness per site

```
res2 <- with(meta3, specnumber(temp4, site, MARGIN = 1))
# res2
```

Accumulation model over all Sites

```
pool <- with(meta3, poolaccum(temp4))
# summary(pool) #display = 'chao'
plot(pool)
```

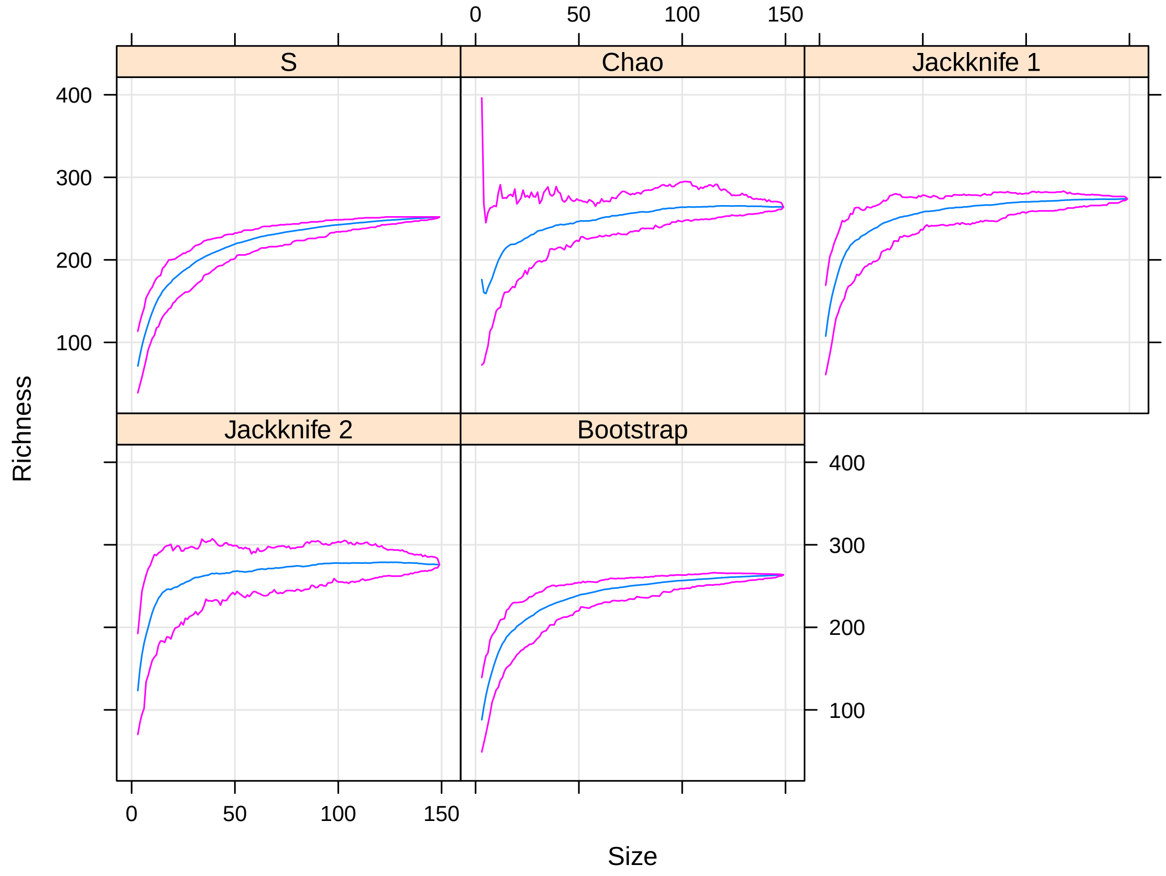

```
dev.off()
```

```
## null device
##           1
```

BOXPLOT : richness Observed per site, coverage per site

```
temp4 <- t(as.data.frame(physeq4_rem_controls@otu_table))
meta4 <- meta(physeq4_rem_controls)
meta4$site2 <- as.factor(meta4$site)
pool1 <- with(meta4, specpool(temp4, site2))
# pool1
split.screen(c(2, 1))
```

```
## [1] 1 2
```

```
split.screen(c(1, 2), 2)
```

```
## [1] 3 4
```

```
screen(1)
b1 = boxplot(specnumber(temp4) ~ site2, data = meta4,
    col = "hotpink", border = "cyan3", las = 2, cex.axis = 0.5,
    cex.lab = 1, cex.main = 1, main = "Boxplot of species number per site",
    xlab = "")
screen(3)
b2 = boxplot(specnumber(temp4)/specpool2vect(pool1,
    index = "jack2") ~ site2, data = meta4, col = "hotpink",
    border = "cyan3", las = 2, cex.axis = 0.5, cex.lab = 0.6,
    cex.main = 1, main = "Coverage (Observed/Jack2)\n  per Librarie X site",
    xlab = "")
screen(4)
b3 = boxplot(specpool2vect(pool1, index = "Species")/specpool2vect(pool1,
    index = "jack2") ~ site2, data = meta4, col = "hotpink",
    border = "cyan3", las = 2, cex.axis = 0.5, cex.lab = 0.6,
    cex.main = 1, main = "Average Coverage (Observed/Jack2)\n  per site",
    xlab = "")
```

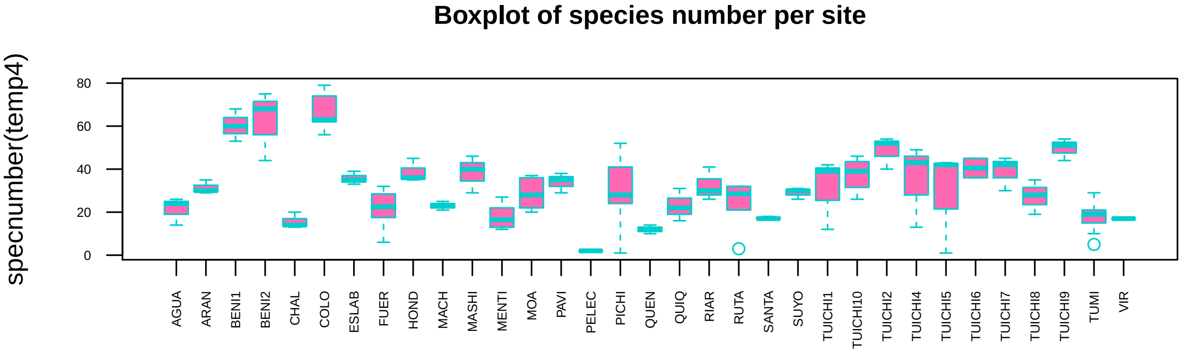

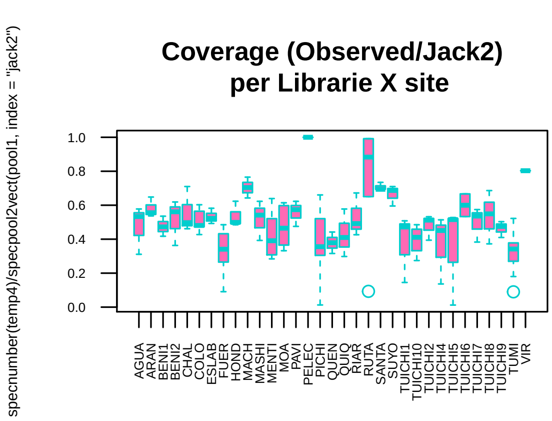

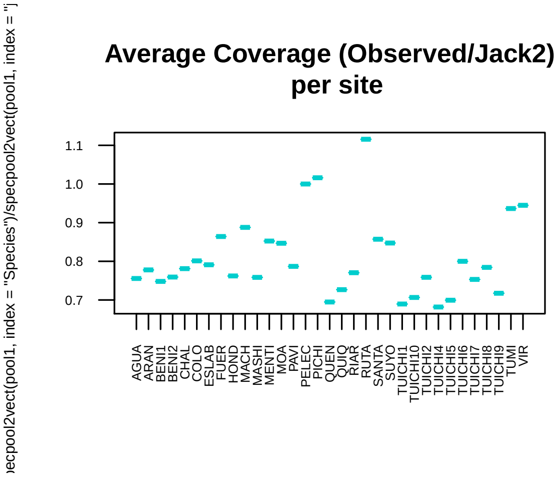


```
dev.off()
```

```
## null device
##           1
```

Richness at site level :"Observed", "Shannon","Simpson","ACE"

using a phyloseq object

```
bidon = merge_samples(physeq4_rem_controls, "site")
estimate_richness(bidon, measures = c("Observed", "Shannon",
    "Simpson", "ACE"))
```

Compute Mean percent ID per taxa

These command lines compute average ID% per taxa using as input file 5200 OTUs with their individual ID
Input file = SUPP DATA Tables and SFigures.xlsx
sheet = "ID%", columns abundance + ID.max.between.H.I.J + taxa_assigned.

```
setwd("C:/Users/mariac/Documents/Documents/ACEDRIC/Programmes/PIMELO-et-ECOBIO/E-DNA manipes/EDNA-2001li
Binary <- read.xlsx("SUPP DATA Tables and SFigures.xlsx",
    sheet = "ID%", rowNames = F, colNames = TRUE, rows = c(2:5248),
    cols = c(11:13))
```

abundance	% identity max)	taxa
2008	98	Acestrorhynchus lacustris
16	97	Acestrorhynchus lacustris
4	100	Acestrorhynchus pantaneiro
14	100	Acestrorhynchus pantaneiro
99	97	Acrobrycon ipanquianus
18	97	Acrobrycon ipanquianus
5	97	Acrobrycon ipanquianus

Create a txt file with these 3 columns : new.txt

computation must be done under cluster, not enough ressources with PC

```
# reserve a node
srun -p short --nodelist=node14 --pty bash -i
module load bioinfo/R/3.6.0
R
library(Rcmdr)
Dataset <- read.table("new.txt", header=TRUE, sep="\t", na.strings="NA",dec=".",
                      strip.white=TRUE)
```

Split Dataset in a table as :

abundance	ID.max_between_H_I_J	taxa_assigned
1	98	Acestrorhynchus lacustris
1	98	Acestrorhynchus lacustris
1	98	Acestrorhynchus lacustris

```
a <- rep(Dataset$ID.max.between.F.G.H, Dataset$abundance)
b <- rep(Dataset$taxa_assigned, Dataset$abundance)
c <- cbind.data.frame(ID.max.between.F.G.H = a, taxa_assigned = b)
d <- numSummary(c[, "ID.max.between.F.G.H", drop = FALSE],
    groups = c$taxa_assigned, statistics = c("mean",
        "sd"))
write.csv(d$table, file = "/home/mariac/d3.csv")
```

Correlation Altitude & richness

Input file : “SUPP DATA Tables and SFigures.xlsx”,sheet = “STable3 metad. site level” , columns “site”, “ALTITUDE” ,“Species”

```
setwd("C:/Users/mariac/Documents/Documents/ACEDRIC/Programmes/PIMELO-et-ECOBIO/E-DNA manipes/EDNA-2001li
```

Correlation and test

```
corela <- read.xlsx("SUPP DATA Tables and SFigures.xlsx",
    sheet = "STable3 metad. site level", rowNames = F,
    colNames = TRUE, rows = c(2:35), cols = c(1, 3,
        4))
res <- cor.test(log(corela$Species), log(corela$ALTITUDE),
    method = "pearson")
plot(corela$Species ~ corela$ALTITUDE)
curve(4459.5 * x^-0.808, from = 1, to = 4500, add = TRUE,
    col = 2)
res
```

Plot of Observed, extrapolated (Jack2) richness and percentage of covered richness

At site level

```
setwd("C:/Users/mariac/Documents/Documents/ACEDRIC/Programmes/PIMELO-et-ECOBIO/E-DNA manipes/EDNA-2001li
coverage <- read.xlsx("SUPP DATA Tables and SFigures.xlsx",
    sheet = "STable3 metad. site level", rowNames = F,
    colNames = TRUE, rows = c(2:35), cols = c(1:8))
p <- ggplot(coverage, aes(x = Site.Name, width = 0.3)) +
    geom_col(aes(y = Jack2), fill = "#00AFBB", position = position_nudge(x = 0)) +
    geom_col(aes(y = Species), fill = "#E7B800", position = position_nudge(x = -0.3)) +
    geom_col(aes(y = Coverage * 4), fill = "#FC4E07",
        position = position_nudge(x = 0.3)) + geom_errorbar(aes(x = Site.Name,
    ymin = (Coverage - IC95b * 100) * 4, ymax = (Coverage +
        IC95h * 100) * 4), size = 0.7, width = 0.15,
    colour = "black", position = position_nudge(x = 0.3)) +
    scale_y_continuous(name = "Number of taxa", sec.axis = sec_axis(~./4,
        name = "%Coverage")) + theme(panel.background = element_blank(),
    axis.title.y.left = element_text(color = "blue"),
    axis.text.y.left = element_text(color = "blue"),
    axis.title.y.right = element_text(color = "red"),
    axis.text.y.right = element_text(color = "red"),
    axis.text.x = element_text(angle = 45, hjust = 1)) +
    labs(title = "Observed, extrapolated (Jack2) richness and percentage of covered richness",
        x = element_blank())

p + theme(axis.line = element_line(colour = "grey"),
    plot.title = element_text(hjust = 0.5))
```

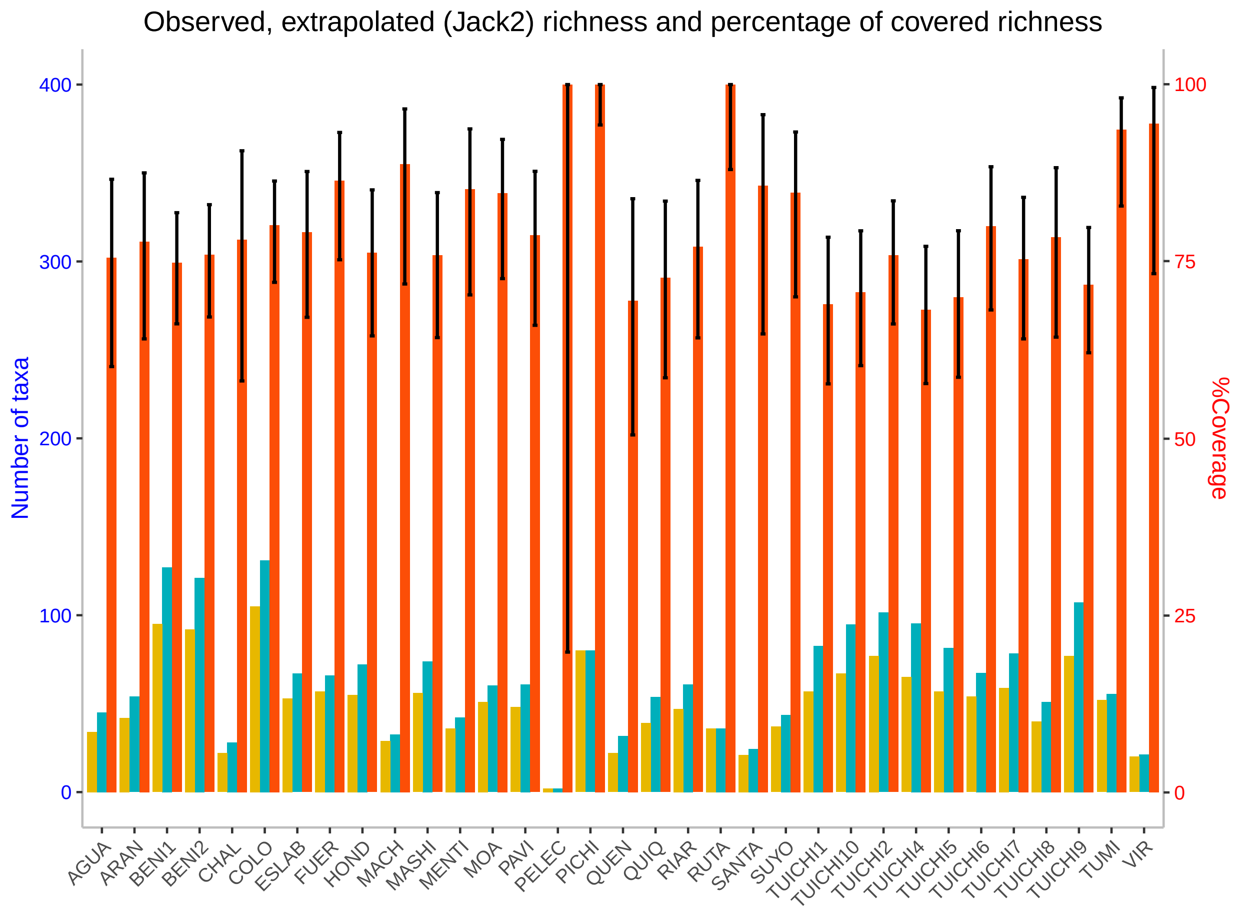

FIGURE 2 : At biogeographic entities level (include all sites)

```
setwd("C:/Users/mariac/Documents/Documents/ACEDRIC/Programmes/PIMELO-et-ECOBIO/E-DNA manipes/EDNA-2001il
coverage <- read.xlsx("SUPP DATA Tables and SFigures.xlsx",
    sheet = "STable3 metad. site level", rowNames = F,
    colNames = TRUE, rows = c(55:61), cols = c(1:8))

p <- ggplot(coverage, aes(x = CLUSTER, width = 0.3)) +
    geom_col(aes(y = Jack2, fill = "Jack2")) + geom_col(aes(y = Species,
    fill = "Species"), position = position_nudge(x = -0.3)) +
    geom_col(aes(y = Coverage * 4, fill = "Coverage"),
        position = position_nudge(x = 0.3)) + geom_errorbar(aes(x = CLUSTER,
    ymin = IC95b * 4, ymax = IC95h * 4), size = 0.9,
    width = 0.15, colour = "black", position = position_nudge(x = 0.3)) +
    scale_y_continuous(name = "Number of taxa", sec.axis = sec_axis(~./4,
        name = "%Coverage")) + theme(panel.background = element_blank(),
    axis.title.y.left = element_text(color = "black",
        size = 20), axis.text.y.left = element_text(color = "black",
        size = 15), axis.title.y.right = element_text(color = "red",
        size = 20), axis.text.y.right = element_text(color = "red",
        size = 15), axis.text.x = element_text(angle = 0,
        hjust = 1)) + labs(title = "Observed, extrapolated (Jack2) richness and percentage of coverage"
```

```r
    x = element_blank())
p + theme(axis.line = element_line(colour = "grey"),
    plot.title = element_text(hjust = 0.5)) + scale_x_discrete(limits = c("Alto-Tuichi",
    "Rank1_2", "Rank3", "Lakes", "Rivers", "ALL")) +
    theme(plot.title = element_text(hjust = 0.5), panel.grid.major = element_blank(),
        panel.grid.minor = element_blank(), panel.background = element_blank(),
        axis.line = element_line(colour = "black")) +
    theme(axis.text.x = element_text(size = 15, hjust = 0.5,
        vjust = 0.5)) + theme(legend.position = c(0.05,
    0.95), legend.title = element_blank()) + scale_fill_manual(labels = c("Coverage",
    "Jack2", "Observed"), values = c(Coverage = "#FC4E07",
    Jack2 = "#00AFBB", Species = "#E7B800"))
```

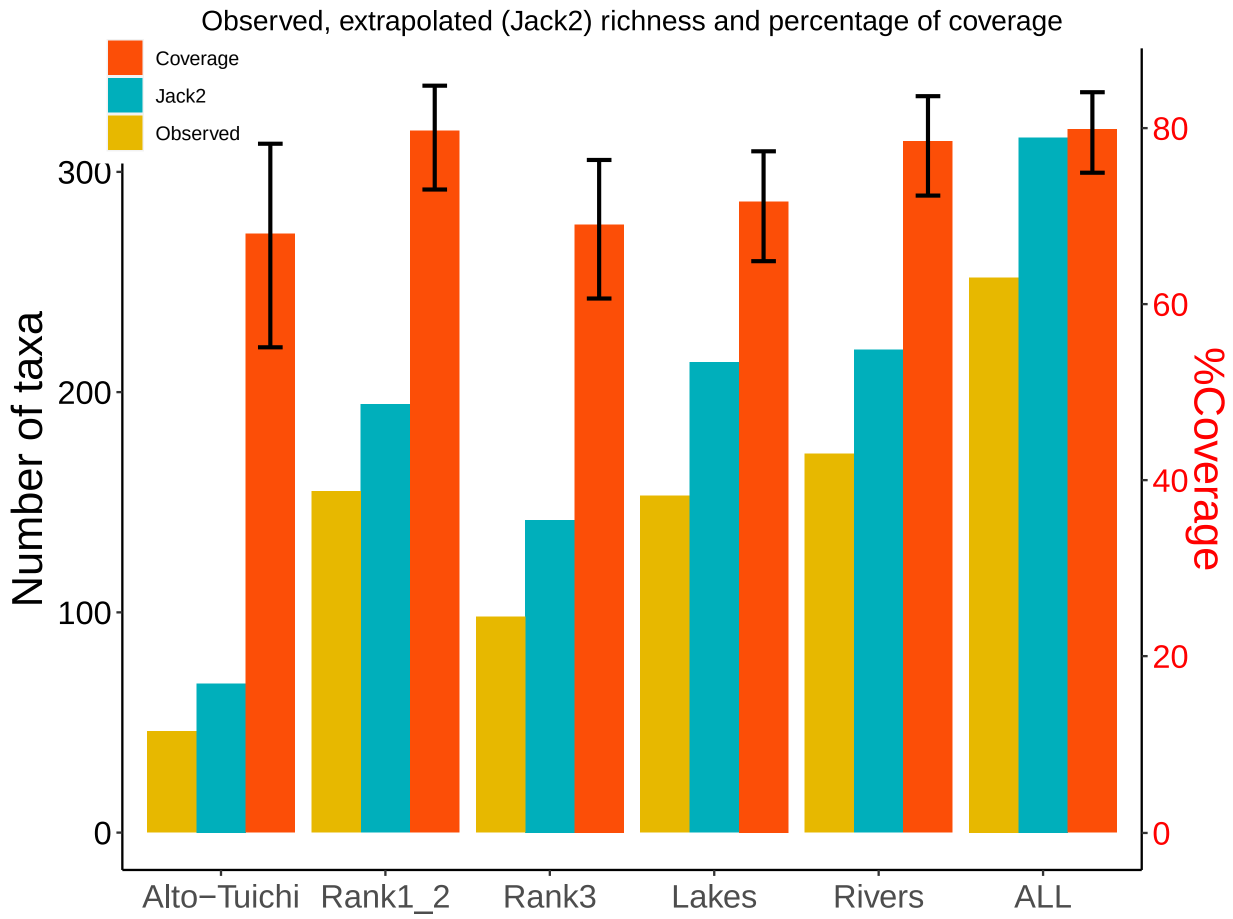


```r
# plot without % coverage

p <- ggplot(coverage, aes(x = CLUSTER, width = 0.3)) +
    geom_col(aes(y = Jack2, fill = "Jack2")) + geom_col(aes(y = Species,
    fill = "Species"), position = position_nudge(x = -0.3)) +
    theme(panel.background = element_blank(), axis.title.y.left = element_text(color = "black",
        size = 20), axis.text.y.left = element_text(color = "black",
        size = 15), axis.text.x = element_text(angle = 45,
        hjust = 1)) + labs(title = "Observed and extrapolated (Jack2) richness",
    x = element_blank())
```

```
p + theme(axis.line = element_line(colour = "grey"),
    plot.title = element_text(hjust = 0.5)) + scale_x_discrete(limits = c("Alto-Tuichi",
    "Rank1_2", "Rank3", "Lakes", "Rivers", "ALL")) +
    theme(plot.title = element_text(hjust = 0.5), panel.grid.major = element_blank(),
        panel.grid.minor = element_blank(), panel.background = element_blank(),
        axis.line = element_line(colour = "black")) +
    theme(axis.text.x = element_text(size = 15, hjust = 0.5,
        vjust = 0.5)) + theme(legend.position = c(0.15,
    0.95), legend.title = element_blank()) + scale_fill_manual(labels = c("Jack2",
    "Observed"), values = c(Jack2 = "#00AFBB", Species = "#E7B800"))
```

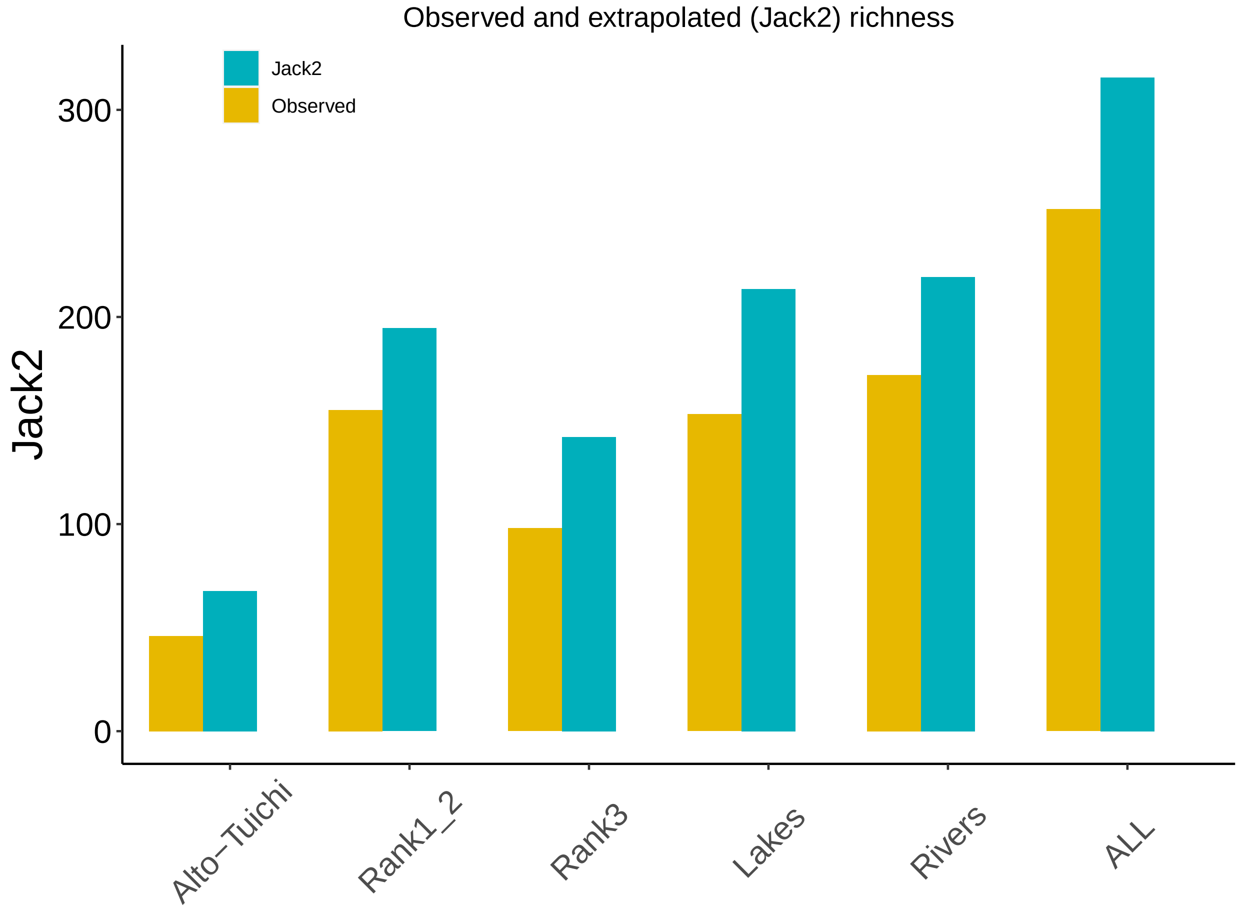

Compare coverage rate

Test percentage of coverage between Biogeographic entities

```
library("rstatix")
library(openxlsx, lib.loc = "C:/Program Files/R/R-4.0.3/library")
setwd("C:/Users/mariac/Documents/Documents/ACEDRIC/Programmes/PIMELO-et-ECOBIO/E-DNA manipes/EDNA-2001li
test_percent <- read.xlsx("SUPP DATA Tables and SFigures.xlsx",
    sheet = "STable3 metad. site level", rowNames = T,
    colNames = TRUE, rows = c(55:61), cols = c(1:3))
test_percent$Jack2 <- ceiling(test_percent$Jack2)
```

```r
test_percent$Coverage <- (round(test_percent$Species/test_percent$Jack2,
    2))
```

```r
# Plot IC95 of coverage for the 4 biogeographic
# entities and all sites

test_percent$Jack2 <- ceiling(test_percent$Jack2)
test_percent$Coverage <- (round(test_percent$Species/test_percent$Jack2,
    2))
n <- nrow(test_percent)
mins <- maxs <- numeric(n)
res <- vector("list", n)
for (i in 1:n) {
    res[[i]] <- prop.test(test_percent$Species[i],
        test_percent$Jack2[i])
    mins[i] <- res[[i]]$conf.int[1]
    maxs[i] <- res[[i]]$conf.int[2]
}

par(mfrow = c(1, 1))
plot(1:n, test_percent$Species/test_percent$Jack2,
    ylim = c(min(mins), max(maxs)), pch = 19, las = 1,
    ylab = "Coverage", xlab = "", bty = "n", xaxt = "n",
    main = "prop.test over Site Coverages")
arrows(1:n, mins, 1:n, maxs, code = 3, angle = 90,
    length = 0.1)
axis(1, 1:n, rownames(test_percent), col = "blue",
    las = 2)
abline(h = sum(test_percent$Species)/sum(test_percent$Jack2),
    lty = 2, col = "orange")  # line of mean over sites
```

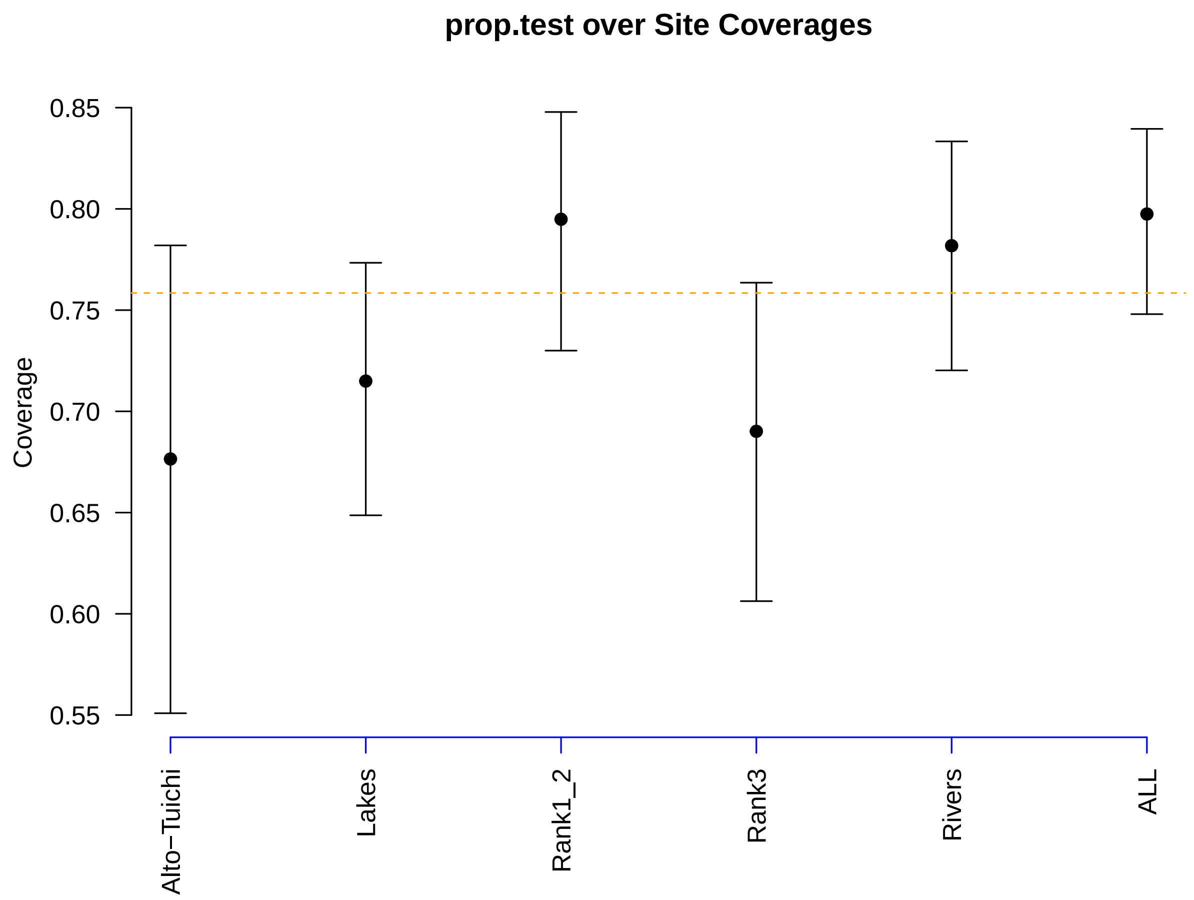


```r
# Test for coverage significativity between 6
# entities
setwd("C:/Users/mariac/Documents/Documents/ACEDRIC/Programmes/PIMELO-et-ECOBIO/E-DNA manipes/EDNA-2001il
test_percent <- read.xlsx("SUPP DATA Tables and SFigures.xlsx",
    sheet = "STable3 metad. site level", rowNames = T,
    colNames = TRUE, rows = c(55:61), cols = c(1:3))  # keep only entities (4)
prop.test(test_percent$Species, test_percent$Jack2,
    correct = F)
```

```
##
##  6-sample test for equality of proportions without continuity
##  correction
##
## data:  test_percent$Species out of test_percent$Jack2
## X-squared = 13.128, df = 5, p-value = 0.02221
## alternative hypothesis: two.sided
## sample estimates:
##    prop 1    prop 2    prop 3    prop 4    prop 5    prop 6
## 0.6798029 0.7163946 0.7967801 0.6903029 0.7846286 0.7984710
```

```r
test_percent[c("lower", "upper")] <- t(sapply(res,
    "[[", "conf.int"))  # return IC95
test_percent
```

```
##              Species     Jack2     lower     upper
## Alto-Tuichi      46  67.66667 0.5508946 0.7819646
## Lakes           153 213.56944 0.6486559 0.7733799
## Rank1_2         155 194.53297 0.7299950 0.8478474
## Rank3            98 141.96667 0.6062601 0.7635485
## Rivers          172 219.21200 0.7202293 0.8333365
## ALL             252 315.60320 0.7480181 0.8395203
```

```
# Test for coverage significativity between the 4
# biogeographic entities (excluding column ALL and
# Rivers merge)
setwd("C:/Users/mariac/Documents/Documents/ACEDRIC/Programmes/PIMELO-et-ECOBIO/E-DNA manipes/EDNA-2001li
test_percent <- read.xlsx("SUPP DATA Tables and SFigures.xlsx",
    sheet = "STable3 metad. site level", rowNames = T,
    colNames = TRUE, rows = c(55:59), cols = c(1:3))  # keep only entities (4)
prop.test(test_percent$Species, test_percent$Jack2,
    correct = F)
```

```
##
##  4-sample test for equality of proportions without continuity
##  correction
##
## data:  test_percent$Species out of test_percent$Jack2
## X-squared = 6.6195, df = 3, p-value = 0.08507
## alternative hypothesis: two.sided
## sample estimates:
##    prop 1    prop 2    prop 3    prop 4
## 0.6798029 0.7163946 0.7967801 0.6903029
```

Test percentage of coverage between sites

```
library("rstatix")
setwd("C:/Users/mariac/Documents/Documents/ACEDRIC/Programmes/PIMELO-et-ECOBIO/E-DNA manipes/EDNA-2001li
test_percent <- read.xlsx("SUPP DATA Tables and SFigures.xlsx",
    sheet = "STable3 metad. site level", rowNames = T,
    colNames = TRUE, rows = c(2:35), cols = c(1, 4,
        5))
test_percent$Jack2 <- ceiling(test_percent$Jack2)
test_percent$Coverage <- (round(test_percent$Species/test_percent$Jack2,
    2))

# row.names.remove <- c('PEL', 'PICHI','RUTA')
# test_percent <-
# test_percent[!(row.names(test_percent) %in%
# row.names.remove), ]
```

```
n <- nrow(test_percent)
mins <- maxs <- numeric(n)
res <- vector("list", n)
for (i in 1:n) {
    res[[i]] <- prop.test(test_percent$Species[i],
```

```
        test_percent$Jack2[i])
    mins[i] <- res[[i]]$conf.int[1]
    maxs[i] <- res[[i]]$conf.int[2]
}

par(mfrow = c(1, 1))
plot(1:n, test_percent$Species/test_percent$Jack2,
    ylim = c(min(mins), max(maxs)), pch = 19, las = 1,
    ylab = "Coverage", xlab = "", bty = "n", xaxt = "n",
    main = "Prop.test over Site Coverages")
arrows(1:n, mins, 1:n, maxs, code = 3, angle = 90,
    length = 0.1)
axis(1, 1:n, rownames(test_percent), col = "blue",
    las = 2)
# add line : mean over sites
abline(h = sum(test_percent$Species)/sum(test_percent$Jack2),
    lty = 2, col = "orange")
```

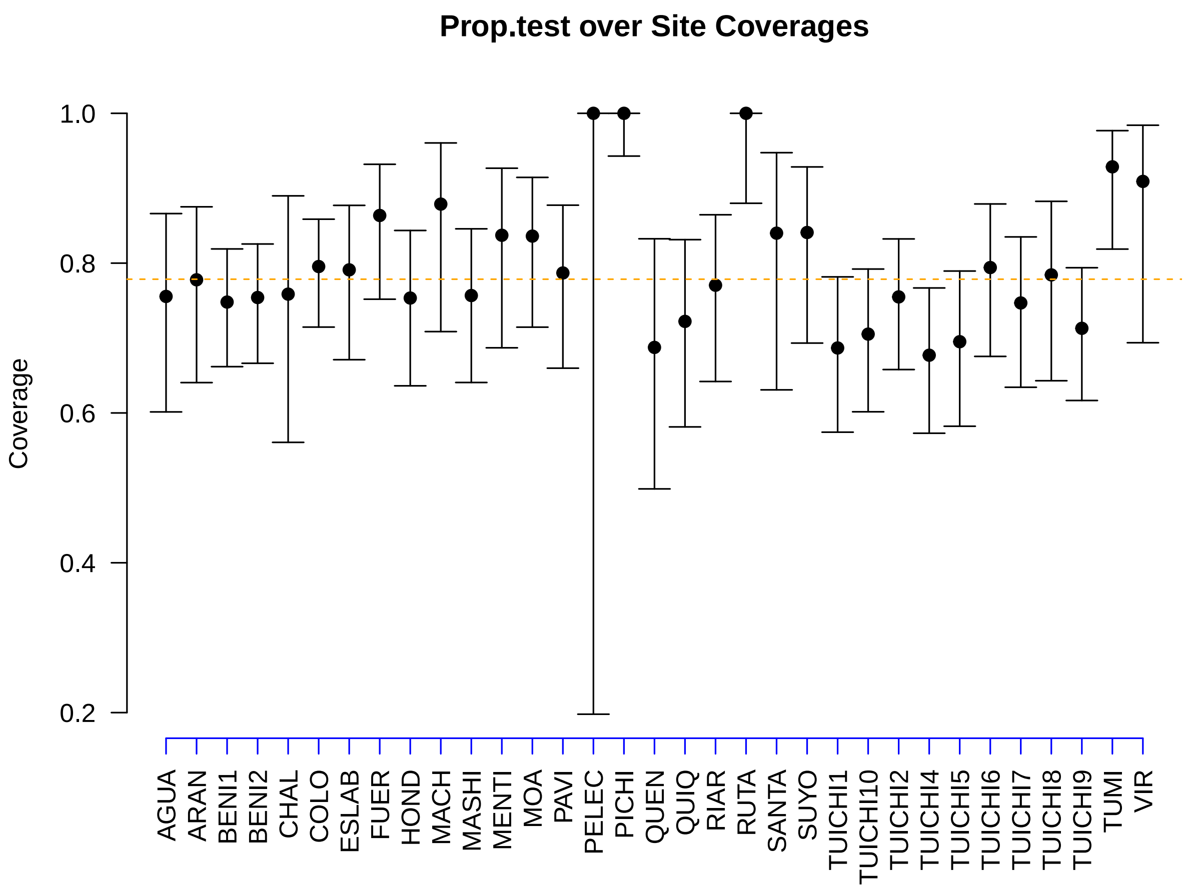


```
# Test for coverage significativity between the 4
# biogeographic entities (excluding column ALL)
prop.test(test_percent$Species, test_percent$Jack2,
    correct = F)
```

```
##
##  33-sample test for equality of proportions without continuity
##  correction
##
## data:  test_percent$Species out of test_percent$Jack2
## X-squared = 75.607, df = 32, p-value = 2.174e-05
## alternative hypothesis: two.sided
## sample estimates:
##    prop 1    prop 2    prop 3    prop 4    prop 5    prop 6    prop 7    prop 8
## 0.7555556 0.7777778 0.7480315 0.7540984 0.7586207 0.7954545 0.7910448 0.8636364
##    prop 9   prop 10   prop 11   prop 12   prop 13   prop 14   prop 15   prop 16
## 0.7534247 0.8787879 0.7567568 0.8372093 0.8360656 0.7868852 1.0000000 1.0000000
##   prop 17   prop 18   prop 19   prop 20   prop 21   prop 22   prop 23   prop 24
## 0.6875000 0.7222222 0.7704918 1.0000000 0.8400000 0.8409091 0.6867470 0.7052632
##   prop 25   prop 26   prop 27   prop 28   prop 29   prop 30   prop 31   prop 32
## 0.7549020 0.6770833 0.6951220 0.7941176 0.7468354 0.7843137 0.7129630 0.9285714
##   prop 33
## 0.9090909
```

```
res
```

```
## [[1]]
##
##  1-sample proportions test with continuity correction
##
## data:  test_percent$Species[i] out of test_percent$Jack2[i], null probability 0.5
## X-squared = 10.756, df = 1, p-value = 0.00104
## alternative hypothesis: true p is not equal to 0.5
## 95 percent confidence interval:
##  0.6013926 0.8661495
## sample estimates:
##         p
## 0.7555556
##
##
## [[2]]
##
##  1-sample proportions test with continuity correction
##
## data:  test_percent$Species[i] out of test_percent$Jack2[i], null probability 0.5
## X-squared = 15.574, df = 1, p-value = 7.933e-05
## alternative hypothesis: true p is not equal to 0.5
## 95 percent confidence interval:
##  0.6405360 0.8751885
## sample estimates:
##         p
## 0.7777778
##
##
## [[3]]
##
##  1-sample proportions test with continuity correction
##
## data:  test_percent$Species[i] out of test_percent$Jack2[i], null probability 0.5
```

```
## X-squared = 30.268, df = 1, p-value = 3.763e-08
## alternative hypothesis: true p is not equal to 0.5
## 95 percent confidence interval:
##  0.6618150 0.8189391
## sample estimates:
##         p
## 0.7480315
##
##
## [[4]]
##
##  1-sample proportions test with continuity correction
##
## data:  test_percent$Species[i] out of test_percent$Jack2[i], null probability 0.5
## X-squared = 30.5, df = 1, p-value = 3.339e-08
## alternative hypothesis: true p is not equal to 0.5
## 95 percent confidence interval:
##  0.6663297 0.8255388
## sample estimates:
##         p
## 0.7540984
##
##
## [[5]]
##
##  1-sample proportions test with continuity correction
##
## data:  test_percent$Species[i] out of test_percent$Jack2[i], null probability 0.5
## X-squared = 6.7586, df = 1, p-value = 0.00933
## alternative hypothesis: true p is not equal to 0.5
## 95 percent confidence interval:
##  0.5607494 0.8898213
## sample estimates:
##         p
## 0.7586207
##
##
## [[6]]
##
##  1-sample proportions test with continuity correction
##
## data:  test_percent$Species[i] out of test_percent$Jack2[i], null probability 0.5
## X-squared = 44.917, df = 1, p-value = 2.056e-11
## alternative hypothesis: true p is not equal to 0.5
## 95 percent confidence interval:
##  0.7146284 0.8586704
## sample estimates:
##         p
## 0.7954545
##
##
## [[7]]
##
##  1-sample proportions test with continuity correction
```

```
##
## data:  test_percent$Species[i] out of test_percent$Jack2[i], null probability 0.5
## X-squared = 21.552, df = 1, p-value = 3.443e-06
## alternative hypothesis: true p is not equal to 0.5
## 95 percent confidence interval:
##  0.6710850 0.8771184
## sample estimates:
##         p
## 0.7910448
##
##
## [[8]]
##
##  1-sample proportions test with continuity correction
##
## data:  test_percent$Species[i] out of test_percent$Jack2[i], null probability 0.5
## X-squared = 33.47, df = 1, p-value = 7.238e-09
## alternative hypothesis: true p is not equal to 0.5
## 95 percent confidence interval:
##  0.7518536 0.9319626
## sample estimates:
##         p
## 0.8636364
##
##
## [[9]]
##
##  1-sample proportions test with continuity correction
##
## data:  test_percent$Species[i] out of test_percent$Jack2[i], null probability 0.5
## X-squared = 17.753, df = 1, p-value = 2.515e-05
## alternative hypothesis: true p is not equal to 0.5
## 95 percent confidence interval:
##  0.6362156 0.8435988
## sample estimates:
##         p
## 0.7534247
##
##
## [[10]]
##
##  1-sample proportions test with continuity correction
##
## data:  test_percent$Species[i] out of test_percent$Jack2[i], null probability 0.5
## X-squared = 17.455, df = 1, p-value = 2.943e-05
## alternative hypothesis: true p is not equal to 0.5
## 95 percent confidence interval:
##  0.7085988 0.9604424
## sample estimates:
##         p
## 0.8787879
##
##
## [[11]]
```

```
##
##  1-sample proportions test with continuity correction
##
## data:  test_percent$Species[i] out of test_percent$Jack2[i], null probability 0.5
## X-squared = 18.5, df = 1, p-value = 1.699e-05
## alternative hypothesis: true p is not equal to 0.5
## 95 percent confidence interval:
##  0.6406816 0.8457968
## sample estimates:
##         p
## 0.7567568
##
##
## [[12]]
##
##  1-sample proportions test with continuity correction
##
## data:  test_percent$Species[i] out of test_percent$Jack2[i], null probability 0.5
## X-squared = 18.233, df = 1, p-value = 1.955e-05
## alternative hypothesis: true p is not equal to 0.5
## 95 percent confidence interval:
##  0.6869784 0.9267247
## sample estimates:
##         p
## 0.8372093
##
##
## [[13]]
##
##  1-sample proportions test with continuity correction
##
## data:  test_percent$Species[i] out of test_percent$Jack2[i], null probability 0.5
## X-squared = 26.23, df = 1, p-value = 3.032e-07
## alternative hypothesis: true p is not equal to 0.5
## 95 percent confidence interval:
##  0.7145315 0.9144514
## sample estimates:
##         p
## 0.8360656
##
##
## [[14]]
##
##  1-sample proportions test with continuity correction
##
## data:  test_percent$Species[i] out of test_percent$Jack2[i], null probability 0.5
## X-squared = 18.951, df = 1, p-value = 1.341e-05
## alternative hypothesis: true p is not equal to 0.5
## 95 percent confidence interval:
##  0.6597951 0.8773900
## sample estimates:
##         p
## 0.7868852
##
```

```
##
## [[15]]
##
##  1-sample proportions test with continuity correction
##
## data:  test_percent$Species[i] out of test_percent$Jack2[i], null probability 0.5
## X-squared = 0.5, df = 1, p-value = 0.4795
## alternative hypothesis: true p is not equal to 0.5
## 95 percent confidence interval:
##  0.1978675 1.0000000
## sample estimates:
## p
## 1
##
##
## [[16]]
##
##  1-sample proportions test with continuity correction
##
## data:  test_percent$Species[i] out of test_percent$Jack2[i], null probability 0.5
## X-squared = 78.013, df = 1, p-value < 2.2e-16
## alternative hypothesis: true p is not equal to 0.5
## 95 percent confidence interval:
##  0.9429074 1.0000000
## sample estimates:
## p
## 1
##
##
## [[17]]
##
##  1-sample proportions test with continuity correction
##
## data:  test_percent$Species[i] out of test_percent$Jack2[i], null probability 0.5
## X-squared = 3.7812, df = 1, p-value = 0.05183
## alternative hypothesis: true p is not equal to 0.5
## 95 percent confidence interval:
##  0.4986377 0.8325051
## sample estimates:
##      p
## 0.6875
##
##
## [[18]]
##
##  1-sample proportions test with continuity correction
##
## data:  test_percent$Species[i] out of test_percent$Jack2[i], null probability 0.5
## X-squared = 9.7963, df = 1, p-value = 0.001749
## alternative hypothesis: true p is not equal to 0.5
## 95 percent confidence interval:
##  0.5813827 0.8313514
## sample estimates:
##         p
```

```
## 0.7222222
##
##
## [[19]]
##
##  1-sample proportions test with continuity correction
##
## data:  test_percent$Species[i] out of test_percent$Jack2[i], null probability 0.5
## X-squared = 16.787, df = 1, p-value = 4.182e-05
## alternative hypothesis: true p is not equal to 0.5
## 95 percent confidence interval:
##  0.6419867 0.8645608
## sample estimates:
##         p
## 0.7704918
##
##
## [[20]]
##
##  1-sample proportions test with continuity correction
##
## data:  test_percent$Species[i] out of test_percent$Jack2[i], null probability 0.5
## X-squared = 34.028, df = 1, p-value = 5.433e-09
## alternative hypothesis: true p is not equal to 0.5
## 95 percent confidence interval:
##  0.8799336 1.0000000
## sample estimates:
## p
## 1
##
##
## [[21]]
##
##  1-sample proportions test with continuity correction
##
## data:  test_percent$Species[i] out of test_percent$Jack2[i], null probability 0.5
## X-squared = 10.24, df = 1, p-value = 0.001374
## alternative hypothesis: true p is not equal to 0.5
## 95 percent confidence interval:
##  0.6308323 0.9474593
## sample estimates:
##    p
## 0.84
##
##
## [[22]]
##
##  1-sample proportions test with continuity correction
##
## data:  test_percent$Species[i] out of test_percent$Jack2[i], null probability 0.5
## X-squared = 19.114, df = 1, p-value = 1.232e-05
## alternative hypothesis: true p is not equal to 0.5
## 95 percent confidence interval:
##  0.6932938 0.9284359
```

```
## sample estimates:
##         p
## 0.8409091
##
##
## [[23]]
##
##  1-sample proportions test with continuity correction
##
## data:  test_percent$Species[i] out of test_percent$Jack2[i], null probability 0.5
## X-squared = 10.843, df = 1, p-value = 0.0009915
## alternative hypothesis: true p is not equal to 0.5
## 95 percent confidence interval:
##  0.5743518 0.7816488
## sample estimates:
##        p
## 0.686747
##
##
## [[24]]
##
##  1-sample proportions test with continuity correction
##
## data:  test_percent$Species[i] out of test_percent$Jack2[i], null probability 0.5
## X-squared = 15.2, df = 1, p-value = 9.67e-05
## alternative hypothesis: true p is not equal to 0.5
## 95 percent confidence interval:
##  0.6015516 0.7921252
## sample estimates:
##         p
## 0.7052632
##
##
## [[25]]
##
##  1-sample proportions test with continuity correction
##
## data:  test_percent$Species[i] out of test_percent$Jack2[i], null probability 0.5
## X-squared = 25.5, df = 1, p-value = 4.424e-07
## alternative hypothesis: true p is not equal to 0.5
## 95 percent confidence interval:
##  0.6579352 0.8323057
## sample estimates:
##        p
## 0.754902
##
##
## [[26]]
##
##  1-sample proportions test with continuity correction
##
## data:  test_percent$Species[i] out of test_percent$Jack2[i], null probability 0.5
## X-squared = 11.344, df = 1, p-value = 0.000757
## alternative hypothesis: true p is not equal to 0.5
```

```
## 95 percent confidence interval:
##  0.5729256 0.7668723
## sample estimates:
##         p
## 0.6770833
##
##
## [[27]]
##
##  1-sample proportions test with continuity correction
##
## data:  test_percent$Species[i] out of test_percent$Jack2[i], null probability 0.5
## X-squared = 11.72, df = 1, p-value = 0.0006185
## alternative hypothesis: true p is not equal to 0.5
## 95 percent confidence interval:
##  0.5822788 0.7894610
## sample estimates:
##        p
## 0.695122
##
##
## [[28]]
##
##  1-sample proportions test with continuity correction
##
## data:  test_percent$Species[i] out of test_percent$Jack2[i], null probability 0.5
## X-squared = 22.368, df = 1, p-value = 2.251e-06
## alternative hypothesis: true p is not equal to 0.5
## 95 percent confidence interval:
##  0.6754842 0.8789880
## sample estimates:
##         p
## 0.7941176
##
##
## [[29]]
##
##  1-sample proportions test with continuity correction
##
## data:  test_percent$Species[i] out of test_percent$Jack2[i], null probability 0.5
## X-squared = 18.278, df = 1, p-value = 1.909e-05
## alternative hypothesis: true p is not equal to 0.5
## 95 percent confidence interval:
##  0.6343016 0.8350123
## sample estimates:
##         p
## 0.7468354
##
##
## [[30]]
##
##  1-sample proportions test with continuity correction
##
## data:  test_percent$Species[i] out of test_percent$Jack2[i], null probability 0.5
```

```
## X-squared = 15.373, df = 1, p-value = 8.826e-05
## alternative hypothesis: true p is not equal to 0.5
## 95 percent confidence interval:
##  0.6430183 0.8824960
## sample estimates:
##         p
## 0.7843137
##
##
## [[31]]
##
##  1-sample proportions test with continuity correction
##
## data:  test_percent$Species[i] out of test_percent$Jack2[i], null probability 0.5
## X-squared = 18.75, df = 1, p-value = 1.49e-05
## alternative hypothesis: true p is not equal to 0.5
## 95 percent confidence interval:
##  0.6166361 0.7938832
## sample estimates:
##        p
## 0.712963
##
##
## [[32]]
##
##  1-sample proportions test with continuity correction
##
## data:  test_percent$Species[i] out of test_percent$Jack2[i], null probability 0.5
## X-squared = 39.446, df = 1, p-value = 3.372e-10
## alternative hypothesis: true p is not equal to 0.5
## 95 percent confidence interval:
##  0.8187475 0.9768697
## sample estimates:
##         p
## 0.9285714
##
##
## [[33]]
##
##  1-sample proportions test with continuity correction
##
## data:  test_percent$Species[i] out of test_percent$Jack2[i], null probability 0.5
## X-squared = 13.136, df = 1, p-value = 0.0002896
## alternative hypothesis: true p is not equal to 0.5
## 95 percent confidence interval:
##  0.6937558 0.9840956
## sample estimates:
##         p
## 0.9090909
```

```
test_percent[c("lower", "upper")] <- t(sapply(res,
    "[[", "conf.int"))  # return IC95
test_percent
```

```
##           Species Jack2 Coverage     lower     upper
## AGUA           34    45     0.76 0.6013926 0.8661495
## ARAN           42    54     0.78 0.6405360 0.8751885
## BENI1          95   127     0.75 0.6618150 0.8189391
## BENI2          92   122     0.75 0.6663297 0.8255388
## CHAL           22    29     0.76 0.5607494 0.8898213
## COLO          105   132     0.80 0.7146284 0.8586704
## ESLAB          53    67     0.79 0.6710850 0.8771184
## FUER           57    66     0.86 0.7518536 0.9319626
## HOND           55    73     0.75 0.6362156 0.8435988
## MACH           29    33     0.88 0.7085988 0.9604424
## MASHI          56    74     0.76 0.6406816 0.8457968
## MENTI          36    43     0.84 0.6869784 0.9267247
## MOA            51    61     0.84 0.7145315 0.9144514
## PAVI           48    61     0.79 0.6597951 0.8773900
## PELEC           2     2     1.00 0.1978675 1.0000000
## PICHI          80    80     1.00 0.9429074 1.0000000
## QUEN           22    32     0.69 0.4986377 0.8325051
## QUIQ           39    54     0.72 0.5813827 0.8313514
## RIAR           47    61     0.77 0.6419867 0.8645608
## RUTA           36    36     1.00 0.8799336 1.0000000
## SANTA          21    25     0.84 0.6308323 0.9474593
## SUYO           37    44     0.84 0.6932938 0.9284359
## TUICHI1        57    83     0.69 0.5743518 0.7816488
## TUICHI10       67    95     0.71 0.6015516 0.7921252
## TUICHI2        77   102     0.75 0.6579352 0.8323057
## TUICHI4        65    96     0.68 0.5729256 0.7668723
## TUICHI5        57    82     0.70 0.5822788 0.7894610
## TUICHI6        54    68     0.79 0.6754842 0.8789880
## TUICHI7        59    79     0.75 0.6343016 0.8350123
## TUICHI8        40    51     0.78 0.6430183 0.8824960
## TUICHI9        77   108     0.71 0.6166361 0.7938832
## TUMI           52    56     0.93 0.8187475 0.9768697
## VIR            20    22     0.91 0.6937558 0.9840956
```

Return CI of coverage per site

```r
library(gridExtra)
library(magick)
x <- prop.test(test_percent$Species, test_percent$Jack2,
    correct = TRUE)
x$p.value
```

```
## [1] 2.173539e-05
```

```r
test_percent[c("lower", "upper")] <- t(sapply(res,
    "[[", "conf.int"))  # return IC95
test_percent$lower <- (round(test_percent$lower, 2))
test_percent$upper <- (round(test_percent$upper, 2))
# test_percent
png(filename = "output.png", width = 600, height = 800,
```

```r
    bg = "white")
grid.table(test_percent)
dev.off()
```

```
## pdf
##   2
```

```r
img <- magick::image_read("output.png")
plot(img)
```

	Species	Jack2	Coverage	lower	upper
AGUA	34	45	0.76	0.6	0.87
ARAN	42	54	0.78	0.64	0.88
BENI1	95	127	0.75	0.66	0.82
BENI2	92	122	0.75	0.67	0.83
CHAL	22	29	0.76	0.56	0.89
COLO	105	132	0.8	0.71	0.86
ESLAB	53	67	0.79	0.67	0.88
FUER	57	66	0.86	0.75	0.93
HOND	55	73	0.75	0.64	0.84
MACH	29	33	0.88	0.71	0.96
MASHI	56	74	0.76	0.64	0.85
MENTI	36	43	0.84	0.69	0.93
MOA	51	61	0.84	0.71	0.91
PAVI	48	61	0.79	0.66	0.88
PELEC	2	2	1	0.2	1
PICHI	80	80	1	0.94	1
QUEN	22	32	0.69	0.5	0.83
QUIQ	39	54	0.72	0.58	0.83
RIAR	47	61	0.77	0.64	0.86
RUTA	36	36	1	0.88	1
SANTA	21	25	0.84	0.63	0.95
SUYO	37	44	0.84	0.69	0.93
TUICHI1	57	83	0.69	0.57	0.78
TUICHI10	67	95	0.71	0.6	0.79
TUICHI2	77	102	0.75	0.66	0.83
TUICHI4	65	96	0.68	0.57	0.77
TUICHI5	57	82	0.7	0.58	0.79
TUICHI6	54	68	0.79	0.68	0.88
TUICHI7	59	79	0.75	0.63	0.84
TUICHI8	40	51	0.78	0.64	0.88
TUICHI9	77	108	0.71	0.62	0.79
TUMI	52	56	0.93	0.82	0.98
VIR	20	22	0.91	0.69	0.98

HAC and HEATMAP

```r
library("heatmap3")
library("pvclust")
library(dendextend)
```

Extract data from physeq5 object :

abundance data merge per SITE excluded : CTRL, IRD and TUICHI3

```
tempo = physeq5
tempo <- prune_taxa(taxa_sums(tempo) > 0, tempo)
mar <- tempo@otu_table
mar <- data.matrix(t(mar))
# convert in matrix
MARf <- as.data.frame(mar)
# remove species without value (freq =0)dim(MARf)
MARF_6p = MARf[rowSums(MARf) > 0, ]
```

Built dendrogram without Boot

Built dendro on sites (translate matrix)

Compute distances between sites

```
tdata.dist <- vegdist(t(MARF_6p), method = "jaccard",
    binary = T)
col.clus_MAR <- hclust(tdata.dist, "ward.D2")
# plot(col.clus_MAR, cex=0.8, main= 'EDNA sites
# clustering')
```

Built dendro on taxas (Original matrix)

Compute distances between taxas

```
data.dist <- vegdist(MARF_6p, method = "jaccard", binary = T)
row.clus_MAR <- hclust(data.dist, "ward.D2")
plot(row.clus_MAR, cex = 0.6, main = "EDNA, species clustering")
```

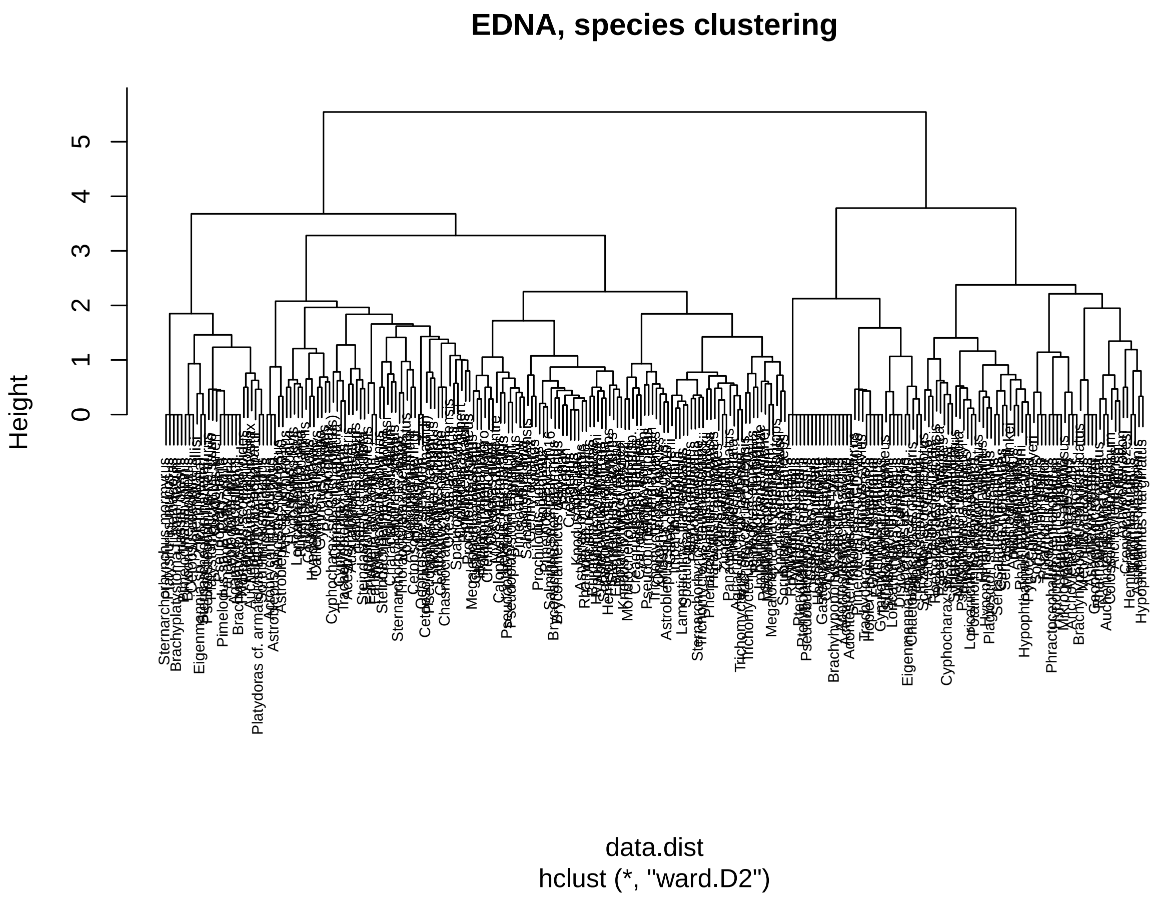


```
row.clus_MAR
```

```
##
## Call:
## hclust(d = data.dist, method = "ward.D2")
##
## Cluster method   : ward.D2
## Distance         : binary jaccard
## Number of objects: 252
```

Built dendrogram with Boot

Built dendro on sites (translate matrix)

```
tresp6_MAR <- pvclust(t(MARF_6p), nboot = 1000, method.dist = function(x) {
    vegan::vegdist(t(x), r = seq(0.5, 1.4, by = 0.01),
        iseed = 666, "jaccard", binary = T)
}, method.hclust = "ward.D2")
par(mfrow = c(1, 1))
plot(tresp6_MAR, main = "EDNA, 1000 boot species",
    cex = 0.7, float = 0.01)
```

Built dendro on taxas (Original matrix)

```r
set.seed(626262)
resp6_MAR <- pvclust(MARF_6p, nboot = 1000, method.dist = function(x) {
    vegan::vegdist(t(x), r = seq(0.2, 1, by = 0.05),
        "jaccard", binary = T)
}, method.hclust = "ward.D2")
```

```
## Bootstrap (r = 0.5)... Done.
## Bootstrap (r = 0.6)... Done.
## Bootstrap (r = 0.7)... Done.
## Bootstrap (r = 0.8)... Done.
## Bootstrap (r = 0.9)... Done.
## Bootstrap (r = 1.0)... Done.
## Bootstrap (r = 1.1)... Done.
## Bootstrap (r = 1.2)... Done.
## Bootstrap (r = 1.3)... Done.
## Bootstrap (r = 1.4)... Done.
```

```r
plot(resp6_MAR, main = "EDNA, 10000 boot Sites, 01062020",
    cex = 0.7)
```

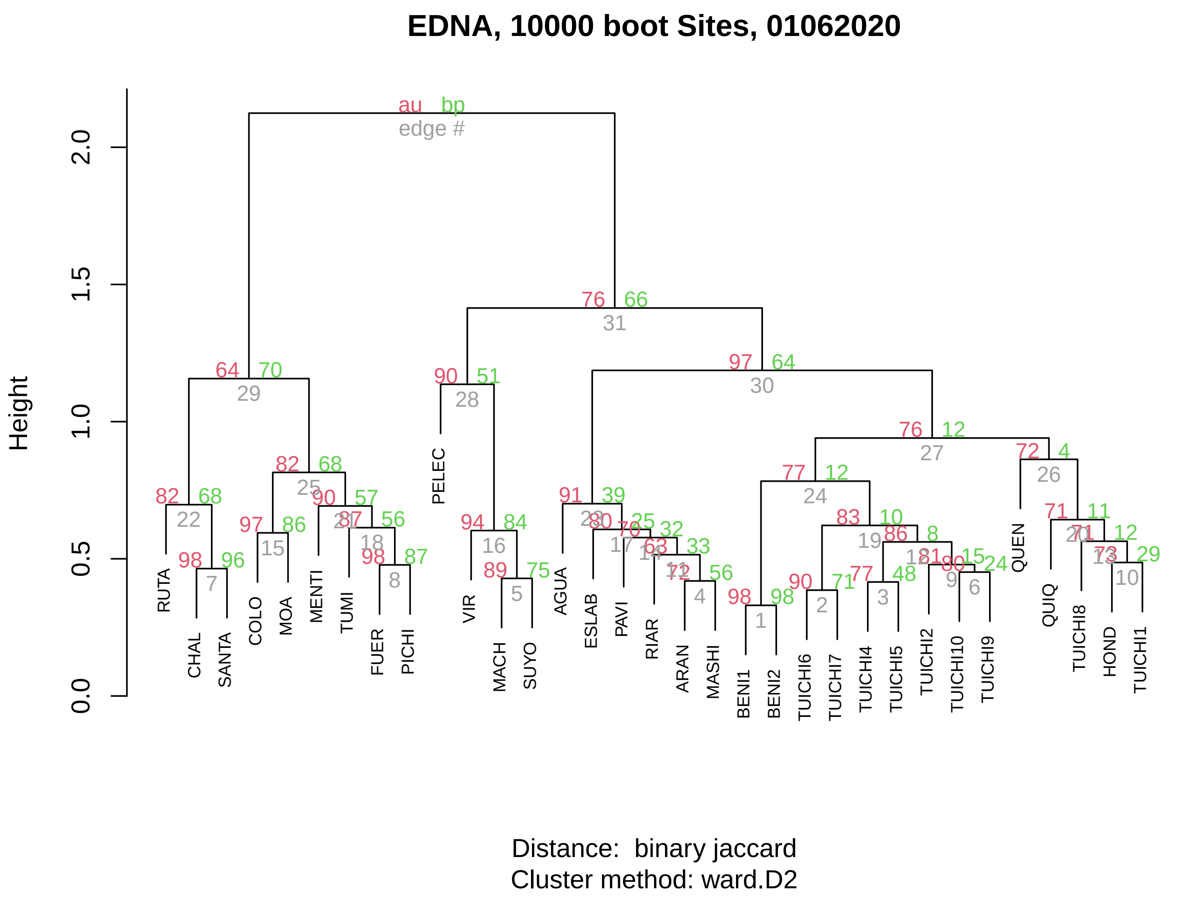

Inertia

```
par(mfrow = c(1, 2))
inertiecol_MAR <- sort(col.clus_MAR$height, decreasing = TRUE)
inertierow_MAR <- sort(row.clus_MAR$height, decreasing = TRUE)
a <- plot(inertiecol_MAR[1:10], type = "s", xlab = "Number of class",
    ylab = "Inertia", main = "Sites")
b <- plot(inertierow_MAR[1:30], type = "s", xlab = "Number of class",
    ylab = "Inertia", main = "Taxa")
```

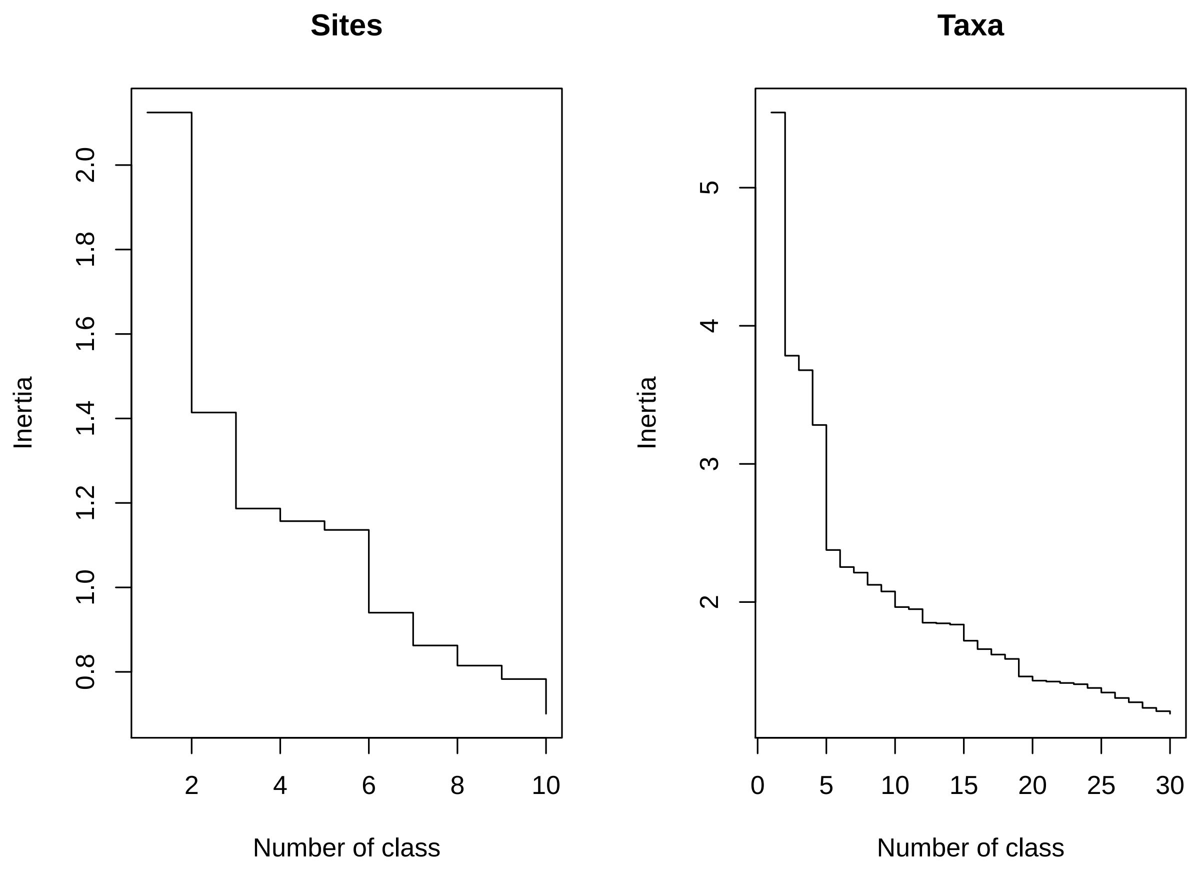


```
par(mfrow = c(1, 1))
```

Standardize abundances

To the minimum number of reads in samples on a phylose object (MEGAN) This makes it possible to have comparable intensity of abundance on the heatmap because the sites do not have the same total number of reads

```
minimum = min(sample_sums(tempo))
# sample_sums(tempo)
standfm = function(x, m = minimum) round(m * (x/sum(x)))
```

```
norm2 = transform_sample_counts(tempo, standfm)
# sample_sums(norm2) Extract abundance matrix from
# the phyloseq object
OTU1 = as(otu_table(norm2), "matrix")
# transpose if necessary
OTU1 <- t(OTU1)
# Coerce to data.frame
OTUdf = as.data.frame(OTU1)
# heatmap3(OTUdf,scale='none',Rowv=NA,Colv=NA,col=
# colorRampPalette(brewer.pal(9,
# 'Blues'),bias=10)(1000))
```

FIGURE4 : HEATMAP

ADD var on graph ATTENTION possible conflits entre fonctions rotate de dendextend et de Venn

```
r <- rownames(OTUdf)
# returns the corresponding Order of taxa (idem recherchev)
newx<-as.data.frame(taxmat$Order[match(r,row.names(taxmat))])
names(newx)[1] <- "V1" # change header de la variable
my_groupmar <- as.numeric(as.factor(newx$V1))
# col_vector<-c('#e6194b', '#3cb44b',  '#4363d8','#008080', '#f58231',
#               '#911eb4', '#46f0f0', '#f032e6', '#bcf60c', '#fabebe',
#               '#ffe119', '#9a6324', '#fffac8', '#800000', '#aaffc3',
#               '#808000', '#ffd8b1', '#000075', '#808080', '#ffffff',
#               '#000000')
# colSidemar <- col_vector[my_groupmar]
#
# pdf("C:/Users/mariac/Desktop/heat2.pdf", height=24, width=18)
#
# h <-heatmap3(as.matrix(OTUdf),scale="none",
#               Rowv =  as.dendrogram(row.clus_MAR),
#               Colv =  as.dendrogram(col.clus_MAR) ,
#               #Colv =(rotate(as.dendrogram(col.clus_MAR), c(1:13,27:33,24:26,14:23))) ,
#               RowSideColors=colSidemar,
#               col= colorRampPalette(brewer.pal(9, "Blues"),bias=10)(5000),
#               cexRow=0.2, cexCol = 1.5,main = " EDNA 01062020",
#               legendfun=function() showLegend(legend=unique(newx$V1),col=unique(colSidemar),cex=2))
# dev.off()

OTUdfb <- OTUdf
OTUdfb[OTUdfb > 1] <- 1
col_vector<-c('#e6194b', '#3cb44b', '#4363d8', '#f58231', '#911eb4', '#46f0f0','#bcf60c',  '#fabebe' ,'#
colSidemar <- col_vector[my_groupmar]

 #show_col(col_vector)

pdf("C:/Users/mariac/Desktop/heat2.pdf", height=24, width=18)

h <-heatmap3(as.matrix(OTUdfb),scale="none",
              Rowv =  as.dendrogram(row.clus_MAR),
              Colv =  as.dendrogram(col.clus_MAR) ,
```

```r
              #Colv =(rotate(as.dendrogram(col.clus_MAR), c(1:13,27:33,24:26,14:23))) ,
              RowSideColors=colSidemar,
              col= colorRampPalette(brewer.pal(5, "Blues"),bias=5)(10),
              cexRow=0.2, cexCol = 1.5,main = " EDNA 01062020",
              legendfun=function() showLegend(legend=unique(newx$V1),col=unique(colSidemar),cex=2,title=
dev.off()
```

```
## pdf
##   2
```

```r
# Plot only species dendrogram https://stackoverflow.com/questions/18802519/label-and-color-leaf-dendrog
Rowv =  as.dendrogram(row.clus_MAR)
par(cex=0.3, mar=c(5, 60, 4, 50))

colorCodes <-colSidemar
labels_colors(Rowv) <- colorCodes[order.dendrogram(Rowv)]

Rowv %>%
  #set("labels_col", colSidemar) %>% # change color
  set("labels_cex", 0.5) %>% # Change size
  set("leaves_pch", 15)%>% # add point
  set("leaves_col", colorCodes[order.dendrogram(Rowv)]) %>%
  set("leaves_cex", 0.8)%>%
  plot(main = "Species dendrogram (Jaccard binary distance)",horiz=T)
```

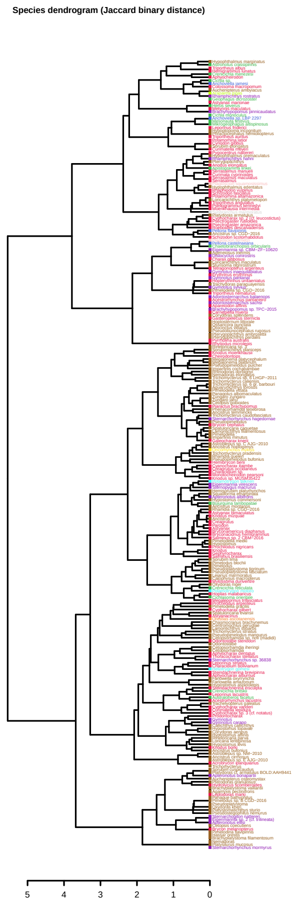


```r
knitr::include_graphics("C:/Users/mariac/Desktop/heat2.pdf")
```

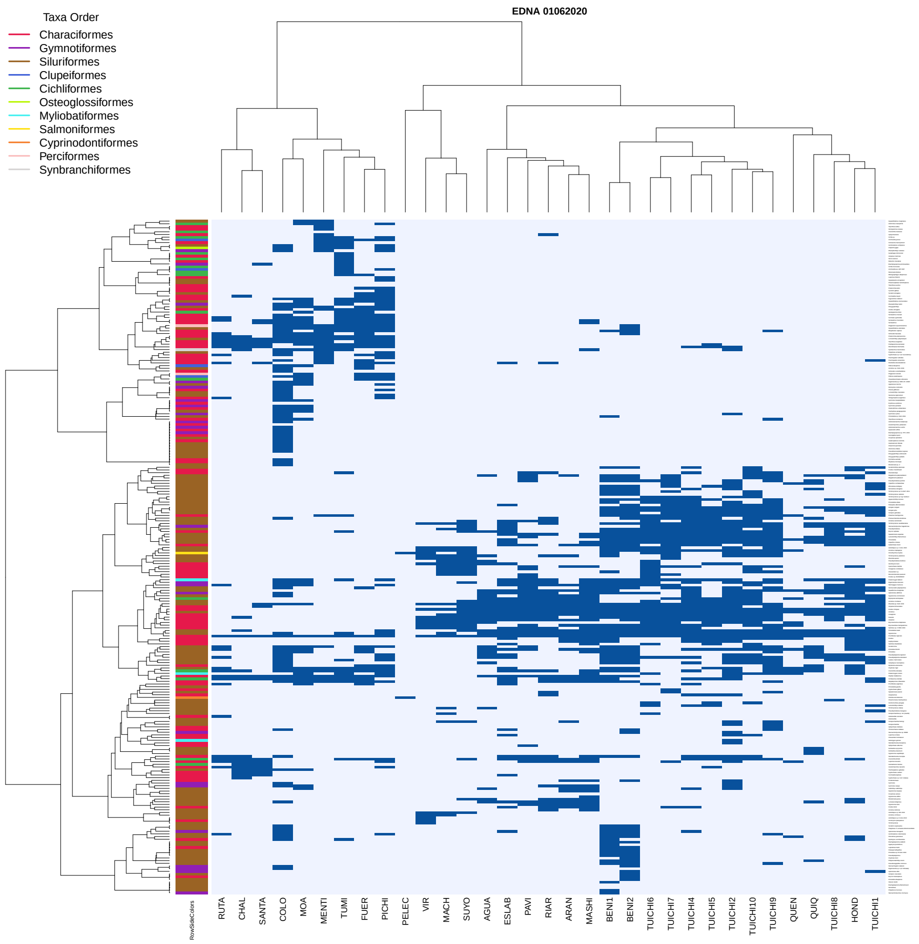

NMDS ordination + environmental variables VEGAN

Notes

Shape file used for environmentale variable
geologic data :https://catalog.data.gov/dataset/south-america-geologic-map-geo6ag
pedologic data : https://datacatalog.worldbank.org/dataset/bolivia-soil-classification/resource/63e7215a-15b3-4a3f-bc89-cc2027f1276d river network :https://www.metis.upmc.fr/en/node/375

Loading Libraries and function

```
# setwd('C:/Users/mariac/Desktop')
library(vegan)
library("plyr")
library(ggrepel)
library("factoextra")
library(maditr)
library(ggpubr)
library(maditr)
find_hull <- function(hull.data) hull.data[chull(hull.data$NMDS1,
    hull.data$NMDS2), ]
```

NMDS ALL SITES (using PHYLOSEQ)

Import data

```
# add a var named 'SampleID' in sam_data
sample_data(physeq5)$SampleID <- sample_data(physeq5)$Site.Name
physeq7 <- physeq5
sample_data(physeq7)$ClusterGroups <- as.character(sample_data(physeq7)$ClusterGroups)
```

Dendrogram

```
mar <- physeq7@otu_table
mar <- data.matrix(t(mar))  # convert in matrix
MARf <- as.data.frame(mar)
MARF_6p = MARf[rowSums(MARf) > 0, ]
tdata.dist <- vegdist(t(MARF_6p), method = "jaccard",
    binary = T)
col.clus_MAR <- hclust(tdata.dist, "ward.D2")
fviz_dend(col.clus_MAR, k = 4, k_colors = c("dodgerblue",
    "red", "forestgreen", "orange"))
```

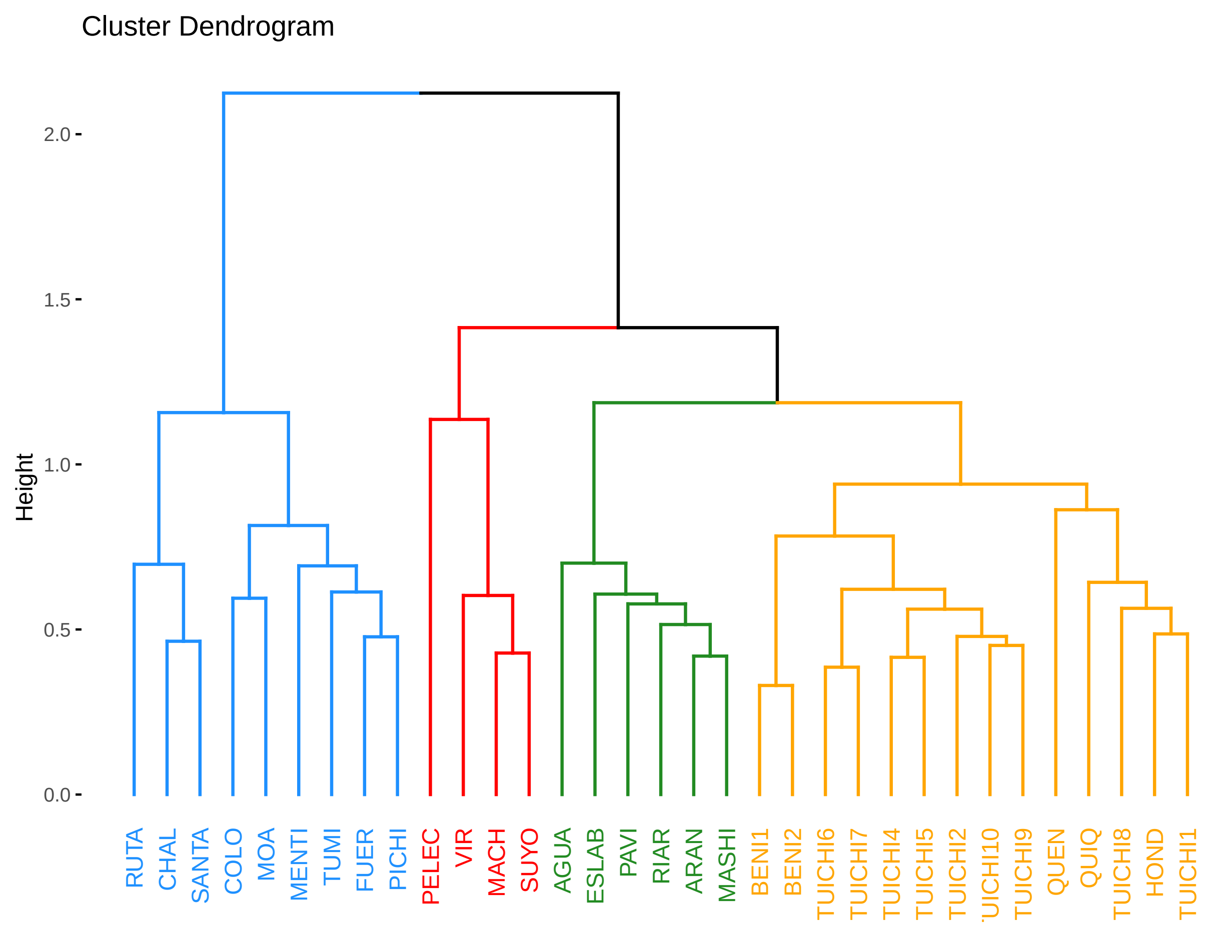

Run ordination on the generated distance

```r
set.seed(62)
ord <- ordinate(physeq7, "NMDS", distance = "jaccard",
    binary = TRUE, k = 2, maxit = 1500, trymax = 1000,
    wascores = TRUE, halfchange = TRUE)
```

Extract scrs

```r
# scores(ord, display = 'sites') scores(ord,
# display = 'species') goodness(ord)
stressplot(ord, main = "33 samples")
```

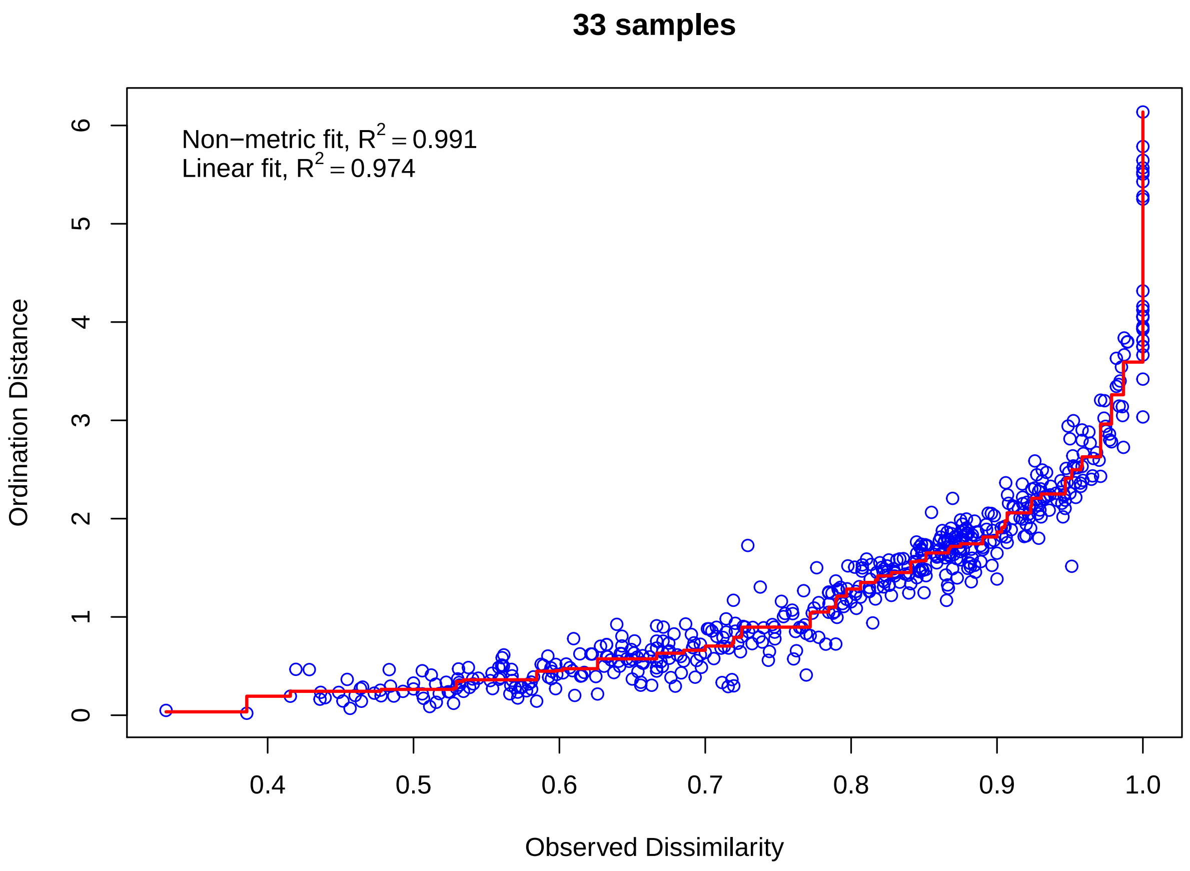


```
ord$stress
```

```
## [1] 0.09402186
```

Define color vector

Extract richness for futur point sizing

```
col_list = c("dodgerblue", "red", "forestgreen", "orange")
pr <- plot_richness(physeq7, x = "SampleID", color = "ECOTYPE",
    measures = c("Observed", "Shannon", "ACE"), nrow = 1)
Richness <- dcast(pr$data, samples + SampleID ~ variable)
```

Preliminary graph

```
# label= 'SampleID'
p0 <- plot_ordination(physeq7, ord, color = "ClusterGroups",
    shape = "ECOTYPE", title = "NMDS jaccard binary") +
    geom_point(aes(size = Richness$Observed)) + theme_bw() +
```

```
    labs(size = "Richness") + scale_color_manual(values = c("dodgerblue",
    "red", "forestgreen", "orange")) + theme_bw() +
    # geom_polygon(data = hulls,mapping =
# aes(NMDS1,NMDS2,group=factor(clustgroups),fill=factor(clustgroups)),
# alpha = 0.2,inherit.aes = FALSE)+
# scale_color_manual(values = col_list) +
scale_fill_manual(values = col_list) + guides(fill = FALSE)
# geom_text_repel(mapping = aes(label =
# SampleID),size = 3, vjust=-3) could add vjust,
# hjust=
p0
```

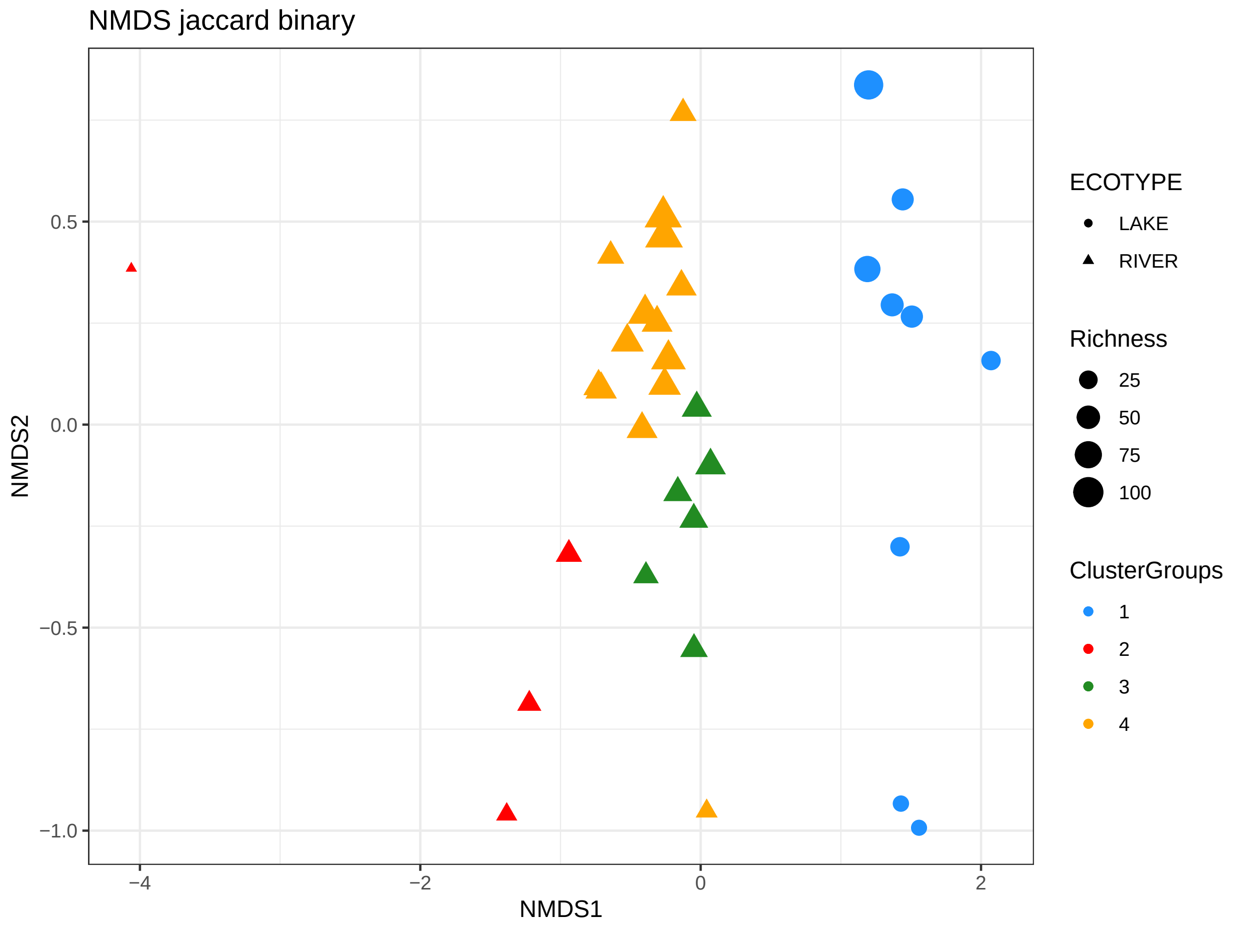

Search for environmental variables that are significantly correlated with NMDS.:

two steps so as not to delete sites for which envir variables are not available. The envit function removes observations for which only one var is missing
not used :+Saturation+Turbidity+TDS+Temperature+Conductity+Salinity+pH+Oxigene

```
ord.fit.tempo <- envfit(ord ~ River.Rank + PEDO + GEOL +
    Latitude + ALTITUDE + Longitude + ECOTYPE + HydroEcoRegion,
    data = as(sample_data(physeq7), "data.frame"),
```

```r
    perm = 10000, na.rm = TRUE)
ord.fit.tempo
```

```
##
## ***VECTORS
##
##               NMDS1    NMDS2     r2    Pr(>r)
## Latitude   0.73735  0.67552 0.2836    0.0147 *
## ALTITUDE  -0.99956 -0.02960 0.5273 9.999e-05 ***
## Longitude  0.67541  0.73744 0.6880 9.999e-05 ***
## ---
## Signif. codes:  0 '***' 0.001 '**' 0.01 '*' 0.05 '.' 0.1 ' ' 1
## Permutation: free
## Number of permutations: 10000
##
## ***FACTORS:
##
## Centroids:
##                       NMDS1   NMDS2
## River.Rank1         -0.2643  0.4878
## River.Rank2         -0.7214  0.0554
## River.Rank3         -0.2615 -0.2962
## River.RankL          1.4645  0.0296
## PEDOAp14-2/3a        1.2762  0.5915
## PEDOGd1-3a           0.5547  0.2508
## PEDOI-Bd-Po-c       -1.0812 -0.5033
## PEDOI-Bd-Rd-c       -0.0055 -0.2368
## PEDOI-Be-c          -4.0615  0.3848
## PEDORd13-1c         -0.3177 -0.1303
## GEOLCarboniferous   -1.3031 -0.8237
## GEOLCretaceous      -0.8342 -0.1125
## GEOLQuaternary       0.6363  0.0472
## GEOLSilurian        -4.0615  0.3848
## GEOLTertiary        -0.3117  0.0638
## ECOTYPELAKE          1.4645  0.0296
## ECOTYPERIVER        -0.5492 -0.0111
## HydroEcoRegionAb-a   1.7888  0.2119
## HydroEcoRegionAb-b   1.2987  0.5174
## HydroEcoRegionAy    -1.0812 -0.5033
## HydroEcoRegionCg    -4.0615  0.3848
## HydroEcoRegionSa-a   0.0428 -0.9519
## HydroEcoRegionSa-b  -0.0549  0.0018
## HydroEcoRegionYa-a  -1.3837 -0.9599
##
## Goodness of fit:
##                     r2    Pr(>r)
## River.Rank      0.5637 9.999e-05 ***
## PEDO            0.5909 9.999e-05 ***
## GEOL            0.6082     2e-04 ***
## ECOTYPE         0.5184 9.999e-05 ***
## HydroEcoRegion  0.7342 9.999e-05 ***
## ---
## Signif. codes:  0 '***' 0.001 '**' 0.01 '*' 0.05 '.' 0.1 ' ' 1
```

```
## Permutation: free
## Number of permutations: 10000
```

```
ord.fit <- envfit(ord ~ River.Rank + P. + Geol. + Latitude +
    ALTITUDE + Longitude + ECOTYPE + HydroEcoRegion,
    data = as(sample_data(physeq7), "data.frame"),
    perm = 10000, na.rm = T)
ord.fit
```

```
##
## ***VECTORS
##
##                NMDS1    NMDS2     r2    Pr(>r)
## Latitude    0.73735  0.67552 0.2836    0.0176 *
## ALTITUDE  -0.99956 -0.02960 0.5273 9.999e-05 ***
## Longitude  0.67541  0.73744 0.6880 9.999e-05 ***
## ---
## Signif. codes:  0 '***' 0.001 '**' 0.01 '*' 0.05 '.' 0.1 ' ' 1
## Permutation: free
## Number of permutations: 10000
##
## ***FACTORS:
##
## Centroids:
##                         NMDS1   NMDS2
## River.Rank1           -0.2643  0.4878
## River.Rank2           -0.7214  0.0554
## River.Rank3           -0.2615 -0.2962
## River.RankL            1.4645  0.0296
## P.Ap14                 1.2762  0.5915
## P.Gd13                 0.5547  0.2508
## P.IBd                 -0.1848 -0.2812
## P.IBe                 -4.0615  0.3848
## P.Rd13                -0.3177 -0.1303
## Geol.C                -0.8342 -0.1125
## Geol.Ca               -1.3031 -0.8237
## Geol.Q                 0.6363  0.0472
## Geol.S                -4.0615  0.3848
## Geol.T                -0.3117  0.0638
## ECOTYPELAKE            1.4645  0.0296
## ECOTYPERIVER          -0.5492 -0.0111
## HydroEcoRegionAb-a  1.7888  0.2119
## HydroEcoRegionAb-b  1.2987  0.5174
## HydroEcoRegionAy   -1.0812 -0.5033
## HydroEcoRegionCg   -4.0615  0.3848
## HydroEcoRegionSa-a  0.0428 -0.9519
## HydroEcoRegionSa-b -0.0549  0.0018
## HydroEcoRegionYa-a -1.3837 -0.9599
##
## Goodness of fit:
##                       r2    Pr(>r)
## River.Rank     0.5637 9.999e-05 ***
## P.             0.5509     2e-04 ***
## Geol.          0.6082 9.999e-05 ***
```

```
## ECOTYPE        0.5184 9.999e-05 ***
## HydroEcoRegion 0.7342 9.999e-05 ***
## ---
## Signif. codes:  0 '***' 0.001 '**' 0.01 '*' 0.05 '.' 0.1 ' ' 1
## Permutation: free
## Number of permutations: 10000
```

```
data.scores = as.data.frame(scores(ord))
data = as(sample_data(physeq7), "data.frame")
data.scores$ClusterGroups = data$ClusterGroups
data.scores$ECOTYPE = data$ECOTYPE
data.scores$Richness = data$Species
arrowhead = arrow(length = unit(0.02, "npc"))
en_coord_cont = as.data.frame(scores(ord.fit, "vectors")) *
    ordiArrowMul(ord.fit, fill = 0.8)
en_coord_cat = as.data.frame(scores(ord.fit, "factors")) *
    ordiArrowMul(ord.fit, fill = 0.55)
rownames(en_coord_cat)[rownames(en_coord_cat) == "HydroEcoRegionAb-a"] <- "HER-Aba"
rownames(en_coord_cat)[rownames(en_coord_cat) == "HydroEcoRegionAb-b"] <- "HER-Abb"
rownames(en_coord_cat)[rownames(en_coord_cat) == "HydroEcoRegionSa-b"] <- "HER-Sab"
rownames(en_coord_cat)[rownames(en_coord_cat) == "HydroEcoRegionAy"] <- "HER-Ay"
rownames(en_coord_cat)[rownames(en_coord_cat) == "HydroEcoRegionCg"] <- "HER-Cg"
rownames(en_coord_cat)[rownames(en_coord_cat) == "HydroEcoRegionSa-a"] <- "HER-Saa"
rownames(en_coord_cat)[rownames(en_coord_cat) == "HydroEcoRegionYa-a"] <- "HER-Yaa"
```

FIGURE 6A : NMDS & environmental variables All sites

For drawing polygons : keep external points

```
hull.data <- merge(scores(ord, display = "sites"), meta(physeq7),by = 0)
hulls <- ddply(hull.data, "ClusterGroups", find_hull)

gg = ggplot(data = data.scores, aes(x = NMDS1, y = NMDS2)) +
  geom_point(data = data.scores, aes(colour = ClusterGroups,shape=ECOTYPE, size=Richness), alpha = 1) +
  scale_colour_manual(values = c("dodgerblue","red","forestgreen","orange"))  +
   scale_size( limits = c(0, 125),breaks =c(20,60,120),range = c(0, 10) )+ #waiver()
  geom_segment(aes(x = 0, y = 0, xend = NMDS1, yend = NMDS2),
             data = en_coord_cont, size =1, alpha = 0.5, colour = "grey30",arrow = arrowhead) +
  geom_point(data = en_coord_cat, aes(x = NMDS1, y = NMDS2),  shape = "diamond", size = 3, alpha = 1, c
  geom_label_repel(data = en_coord_cat, aes(x = NMDS1, y = NMDS2),force= 20,label.padding= 0.2, size = 
  label = row.names(en_coord_cat), colour = "navy", fontface = "bold") +
  geom_label(data = en_coord_cont, aes(x = NMDS1, y = NMDS2), colour = "grey30",  size = 3,
            fontface = "bold", label = row.names(en_coord_cont), hjust=1.2) + #force= 10,label.padding
  theme(axis.title = element_text(size = 10, face = "bold", colour = "grey30"),
        panel.background = element_blank(), panel.border = element_rect(fill = NA, colour = "grey30"),
        axis.ticks = element_blank(), axis.text = element_blank(), legend.key = element_blank(),
        legend.title = element_text(size = 10, face = "bold", colour = "grey30"),
        legend.text = element_text(size = 9, colour = "grey30")) +
  labs(colour = "ClusterGroups")+
    geom_polygon(data = hulls,mapping = aes(NMDS1,NMDS2,group=factor(ClusterGroups),
  fill=factor(ClusterGroups)), alpha = 0.2,inherit.aes = TRUE)+
  scale_color_manual(values = col_list) +
  scale_fill_manual(values = col_list)+
```

```
  theme_bw()+
  guides( fill=FALSE)+
  labs(size="Richness",title = paste0("All SITES, NMDS Jaccard Binary, stress = ", round(ord$stress,2))

gg + theme(legend.position="none")
```

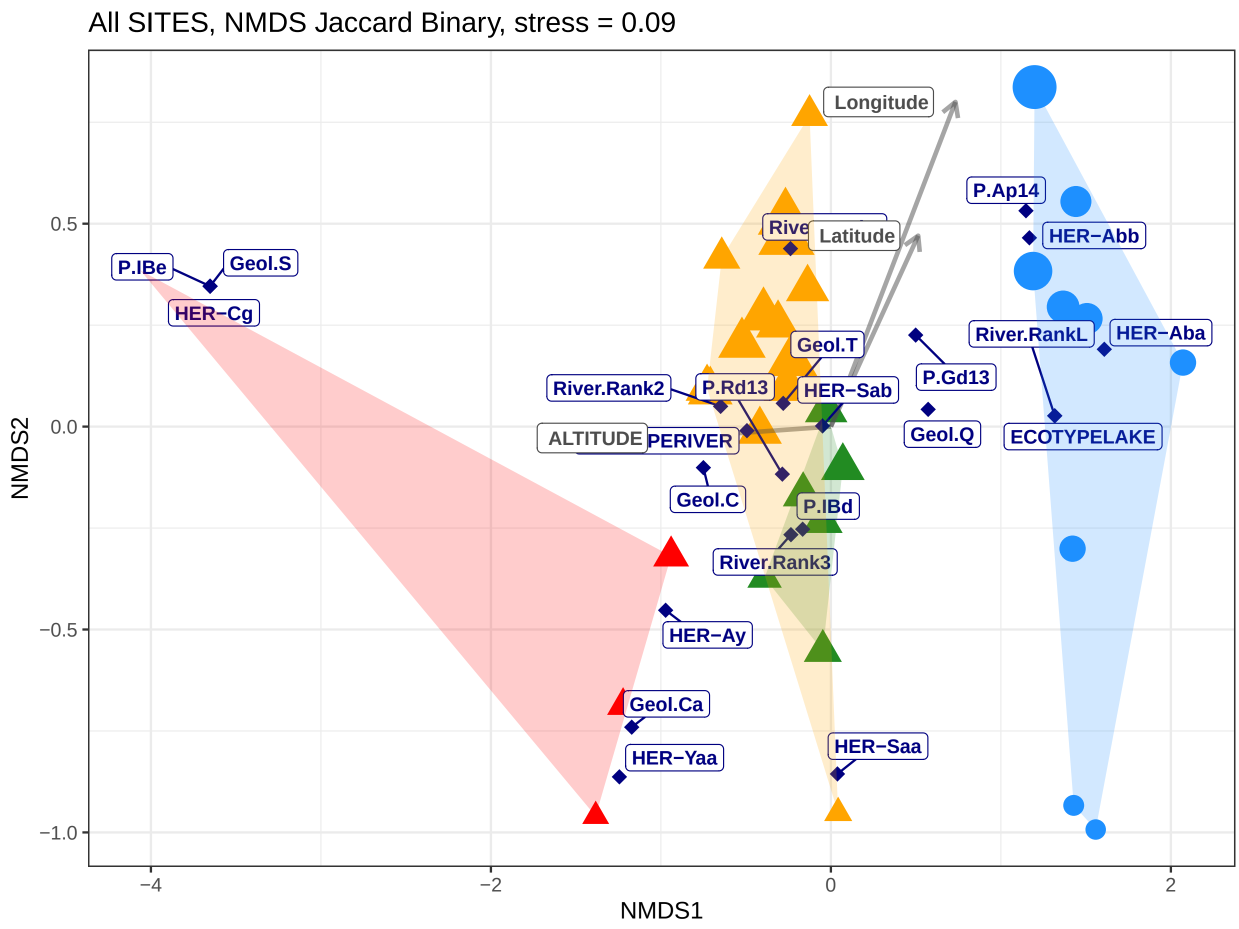


```
gg1 <- gg + labs(color ="Biogeographic entities")

gg1 + guides(color = guide_legend(override.aes = list(size = 5, pch=15)))+
      guides(shape = guide_legend(override.aes = list(size = 5)))+
      guides(size = guide_legend(override.aes = list(pch=17)))
```

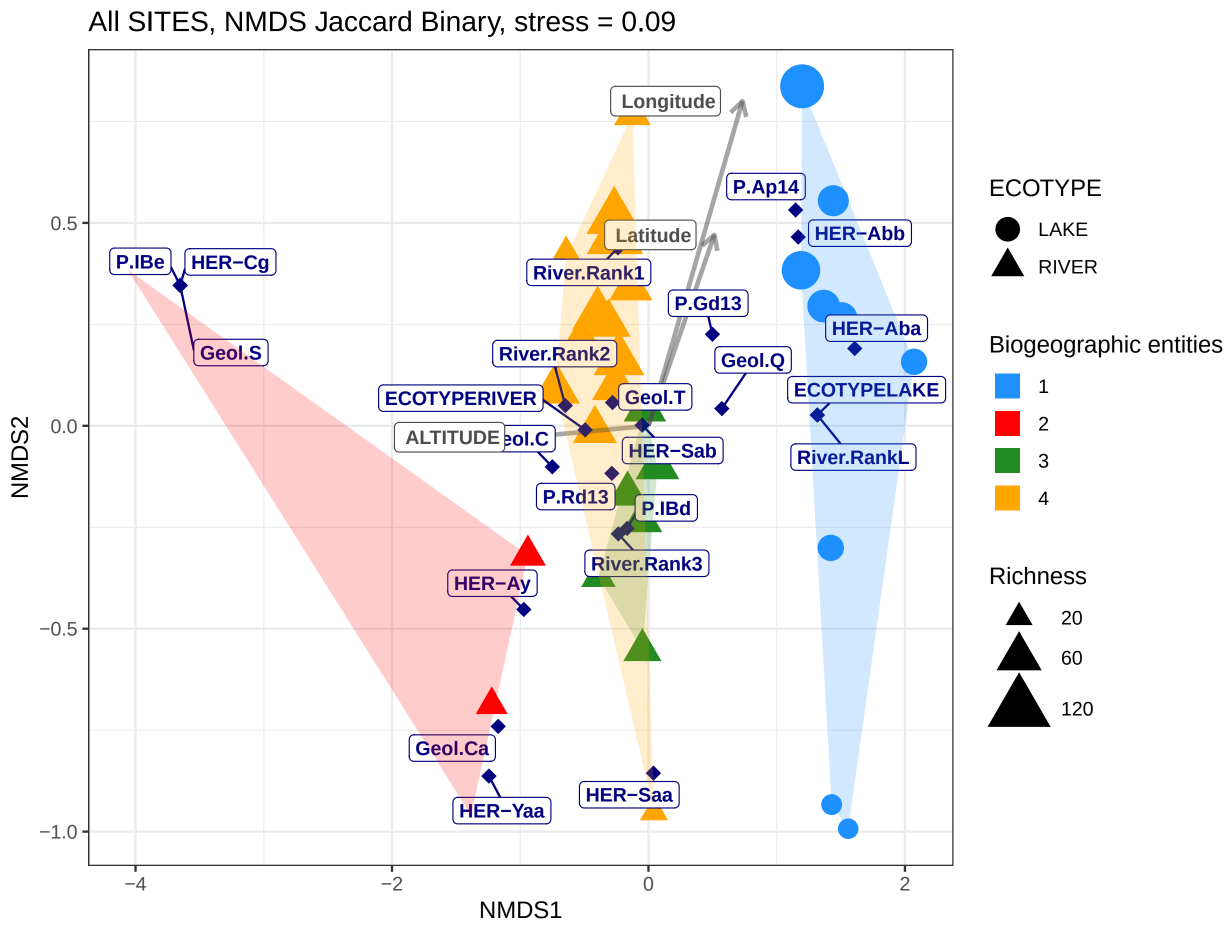

NMDS RIVERS sites (using PHYLOSEQ)

Import data

```
# add a var named 'SampleID' in sam_data

sample_data(physeq5)$SampleID <- sample_data(physeq5)$Site.Name
physeq7 <- subset_samples(physeq5, ECOTYPE == "RIVER" &
    SampleID != "PELEC")
sample_data(physeq7)$ClusterGroups <- as.character(sample_data(physeq7)$ClusterGroups)
```

Run ordination on the generated distance

```
set.seed(62)
ord <- ordinate(physeq7, "NMDS", distance = "jaccard",
    binary = TRUE, k = 2, maxit = 1500, trymax = 1000,
    wascores = TRUE)
```

Extract scrs

```r
# scores(ord, display = 'sites') scores(ord,
# display = 'species') goodness(ord)
stressplot(ord)
```

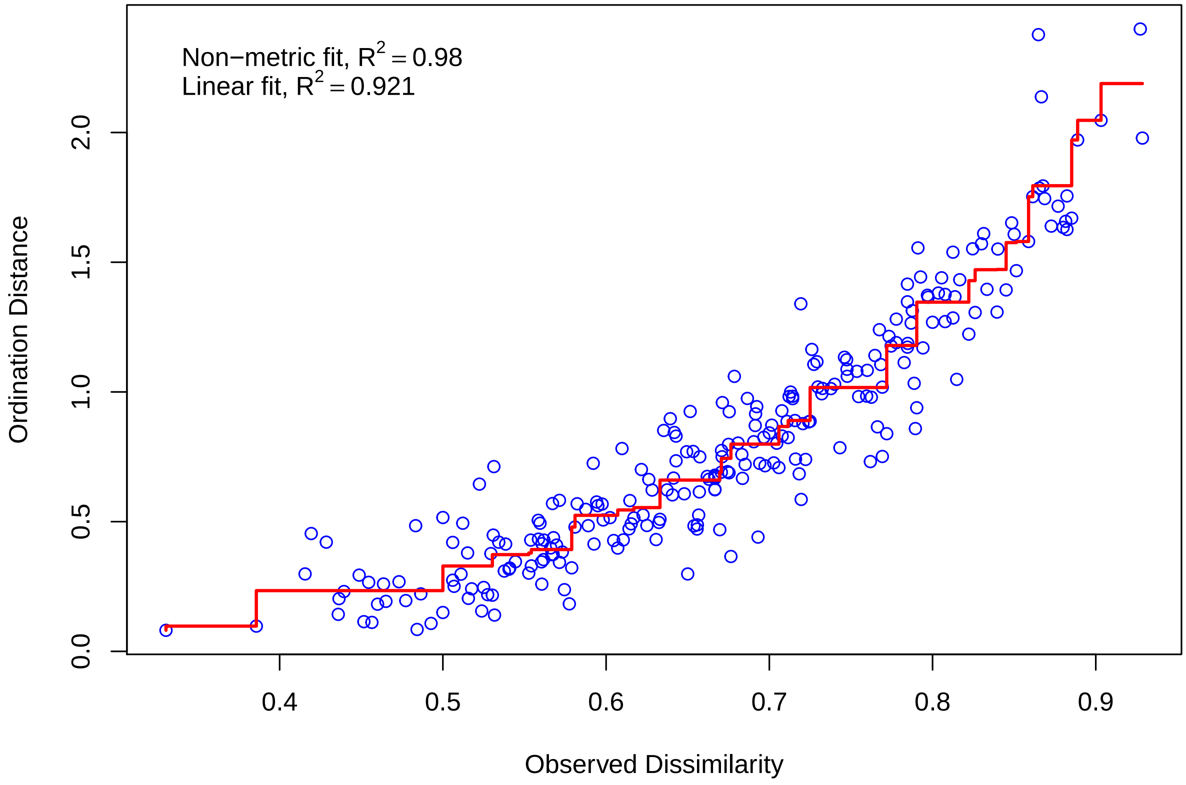


```r
ord$stress
```

```
## [1] 0.141246
```

Define color vector

```r
col_list = c("red", "forestgreen", "orange")
```

Extract richness for futur point sizing

```r
pr2 <- plot_richness(physeq7, x = "SampleID", color = "ECOTYPE",
    measures = c("Observed", "Shannon", "ACE"), nrow = 1)
Richness <- dcast(pr2$data, samples + SampleID ~ variable)
```

Preliminary graph

```
p0 <- plot_ordination(physeq7,
                      ord,
                      color = "ClusterGroups",
                      shape="ECOTYPE",
                      type="sites",
                      #label= "SampleID",
                      title = "NMDS jaccard binary") +
  scale_shape_manual(  values = c(17))+
  scale_color_manual(values=c("red","forestgreen","orange"))+
  geom_point(aes(size=Richness$Observed)) +
  theme_bw()+
  geom_text_repel(mapping = aes(label = SampleID),size = 3, vjust = 3)
p0
```

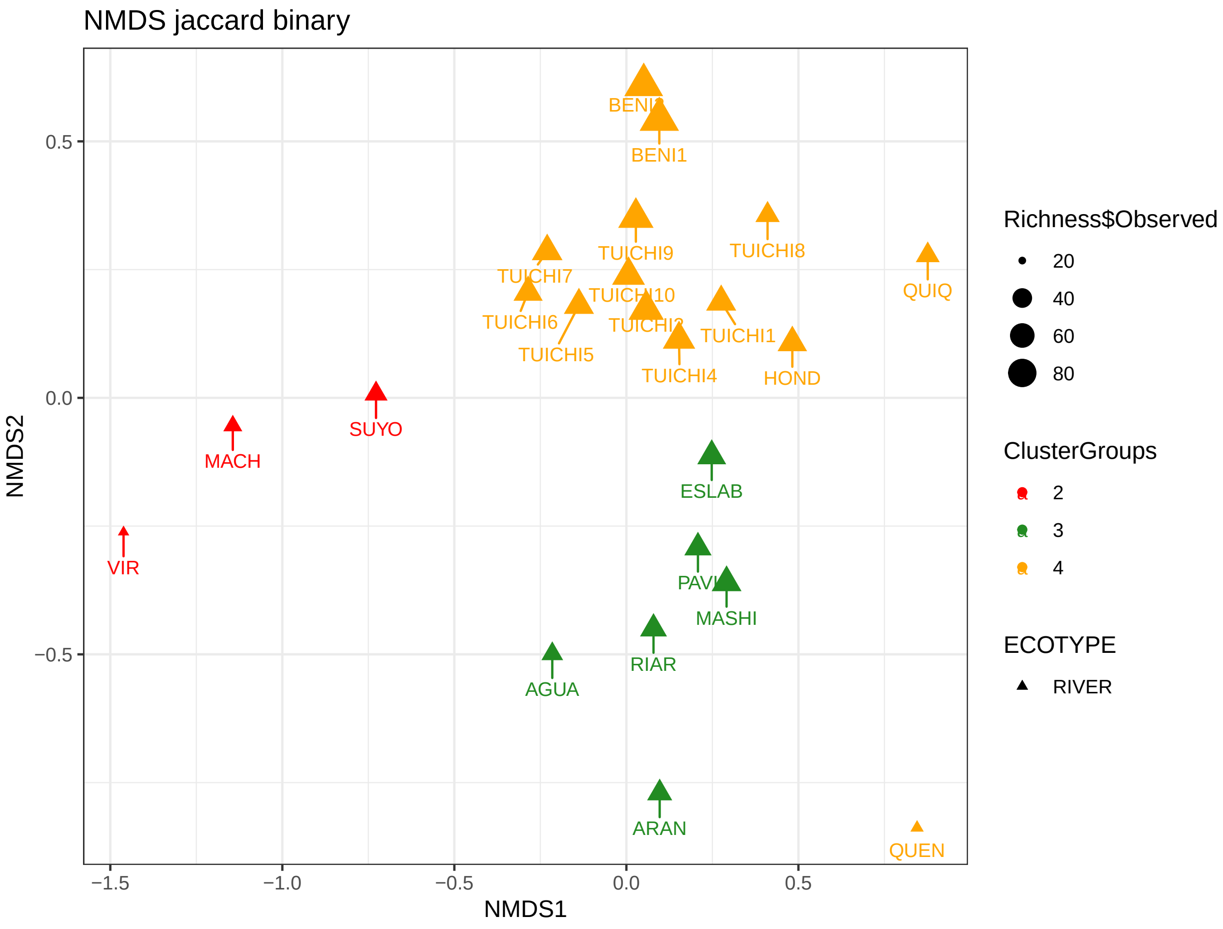

Search for environmental variables that are significantly correlated with NMDS

```
# ord.fit1 <- envfit(ord ~
# Saturation+Turbidity+TDS+Temperature+Conductity+Salinity+pH+Oxigene
```

```r
# , data=as(sample_data(physeq7), 'data.frame'),
# perm=10000, na.rm=TRUE) ord.fit1
ord.fit2 <- envfit(ord ~ PEDO + Latitude + ALTITUDE +
    Longitude + GEOL + HydroEcoRegion, data = as(sample_data(physeq7),
    "data.frame"), perm = 10000, na.rm = TRUE)
ord.fit2
```

```
##
## ***VECTORS
##
##              NMDS1    NMDS2     r2    Pr(>r)
## Latitude  -0.76181  0.64780 0.1608    0.1805
## ALTITUDE  -0.95768 -0.28784 0.7845 9.999e-05 ***
## Longitude  0.92932  0.36926 0.7310 9.999e-05 ***
## ---
## Signif. codes:  0 '***' 0.001 '**' 0.01 '*' 0.05 '.' 0.1 ' ' 1
## Permutation: free
## Number of permutations: 10000
##
## ***FACTORS:
##
## Centroids:
##                       NMDS1   NMDS2
## PEDOGd1-3a           0.1863  0.3598
## PEDOI-Bd-Po-c       -0.9358 -0.0233
## PEDOI-Bd-Rd-c        0.1238 -0.0139
## PEDORd13-1c         -0.0063 -0.2051
## GEOLCarboniferous   -1.3027 -0.1581
## GEOLCretaceous      -0.5067  0.1068
## GEOLQuaternary       0.2304 -0.0658
## GEOLTertiary         0.1545  0.0695
## HydroEcoRegionAy    -0.9358 -0.0233
## HydroEcoRegionSa-a   0.8449 -0.8380
## HydroEcoRegionSa-b   0.1310  0.0603
## HydroEcoRegionYa-a -1.4615 -0.2617
##
## Goodness of fit:
##                    r2    Pr(>r)
## PEDO           0.3122    0.0201 *
## GEOL           0.4922    0.0012 **
## HydroEcoRegion 0.5934 9.999e-05 ***
## ---
## Signif. codes:  0 '***' 0.001 '**' 0.01 '*' 0.05 '.' 0.1 ' ' 1
## Permutation: free
## Number of permutations: 10000
```

```r
# PEDO+Longitude+GEOL+GEOL+pH+Salinity+Conductivity+Temperature+TDS+Longitude+ALTITUDE
ord.fit11 <- envfit(ord ~ ALTITUDE + Longitude, data = as(sample_data(physeq7),
    "data.frame"), perm = 10000, na.rm = TRUE)

ord.fit22 <- envfit(ord ~ P. + Geol. + HydroEcoRegion,
    data = as(sample_data(physeq7), "data.frame"),
    perm = 10000, na.rm = TRUE)
```

```r
data.scores = as.data.frame(scores(ord))
data = as(sample_data(physeq7), "data.frame")
data.scores$ClusterGroups = data$ClusterGroups
data.scores$ECOTYPE = data$ECOTYPE
data.scores$Richness = data$Species
arrowhead = arrow(length = unit(0.02, "npc"))
en_coord_cont = as.data.frame(scores(ord.fit11, "vectors"))  #* ordiArrowMul(ord.fit11, fill=1)
en_coord_cat = as.data.frame(scores(ord.fit22, "factors"))  #* ordiArrowMul(ord.fit22, fill=0.1)

rownames(en_coord_cat)[rownames(en_coord_cat) == "HydroEcoRegionAb-a"] <- "HER-Aba"
rownames(en_coord_cat)[rownames(en_coord_cat) == "HydroEcoRegionAb-b"] <- "HER-Abb"
rownames(en_coord_cat)[rownames(en_coord_cat) == "HydroEcoRegionSa-b"] <- "HER-Sab"
rownames(en_coord_cat)[rownames(en_coord_cat) == "HydroEcoRegionAy"] <- "HER-Ay"
rownames(en_coord_cat)[rownames(en_coord_cat) == "HydroEcoRegionCg"] <- "HER-Cg"
rownames(en_coord_cat)[rownames(en_coord_cat) == "HydroEcoRegionSa-a"] <- "HER-Saa"
rownames(en_coord_cat)[rownames(en_coord_cat) == "HydroEcoRegionYa-a"] <- "HER-Yaa"
```

FIGURE 6B : NMDS & environmentales variables RIVERS sites

For drawing polygons : keep external points

```r
col_list=c("red","forestgreen","orange")
hull.data <- merge(scores(ord, display = "sites"), meta(physeq7),by = 0)
hulls <- ddply(hull.data, "ClusterGroups", find_hull)

gg = ggplot(data = data.scores, aes(x = NMDS1, y = NMDS2)) +
  geom_point(data = data.scores, aes(colour = ClusterGroups, size=Richness),shape=17, alpha = 1) +
  scale_colour_manual(values = col_list)  +
  scale_size( limits = c(0, 125),breaks =c(20,60,120),range = c(0, 10) )+ #waiver()
  geom_segment(aes(x = 0, y = 0, xend = NMDS1, yend = NMDS2),
               data = en_coord_cont, size =1, alpha = 0.5, colour = "grey30",arrow = arrowhead) +
  geom_point(data = en_coord_cat, aes(x = NMDS1, y = NMDS2),
             shape = "diamond", size = 3, alpha = 1, colour = "navy") +
  geom_label_repel(data = en_coord_cat, aes(x = NMDS1, y = NMDS2),force= 10,label.padding= 0.2, size = 
                   label = row.names(en_coord_cat), colour = "navy", fontface = "bold") +
  geom_label_repel(data = en_coord_cont, aes(x = NMDS1, y = NMDS2), colour = "grey30",  size = 3,
                   fontface = "bold", force= 3,label.padding= 0.2,label = row.names(en_coord_cont),vjus
  theme(axis.title = element_text(size = 10, face = "bold", colour = "grey30"),
        panel.background = element_blank(), panel.border = element_rect(fill = NA, colour = "grey30"),
        axis.ticks = element_blank(), axis.text = element_blank(), legend.key = element_blank(),
        legend.title = element_text(size = 10, face = "bold", colour = "grey30"),
        legend.text = element_text(size = 9, colour = "grey30")) +
  labs(colour = "ClusterGroups")+
  geom_polygon(data = hulls,mapping = aes(NMDS1,NMDS2,group=factor(ClusterGroups),fill=factor(ClusterGr
  scale_color_manual(values = col_list) +
  scale_fill_manual(values = col_list)+
  theme_bw()+
  guides( fill=FALSE)+
  labs(size="Richness",title = paste0("ALL RIVER SITES but Pelechuco, NMDS Jaccard Binary, stress = ", 

# gg+ guides(color=guide_legend(title="Biogeographic entities"))
# Remove title for all legends
```

```
gg + theme(legend.position="none")
```

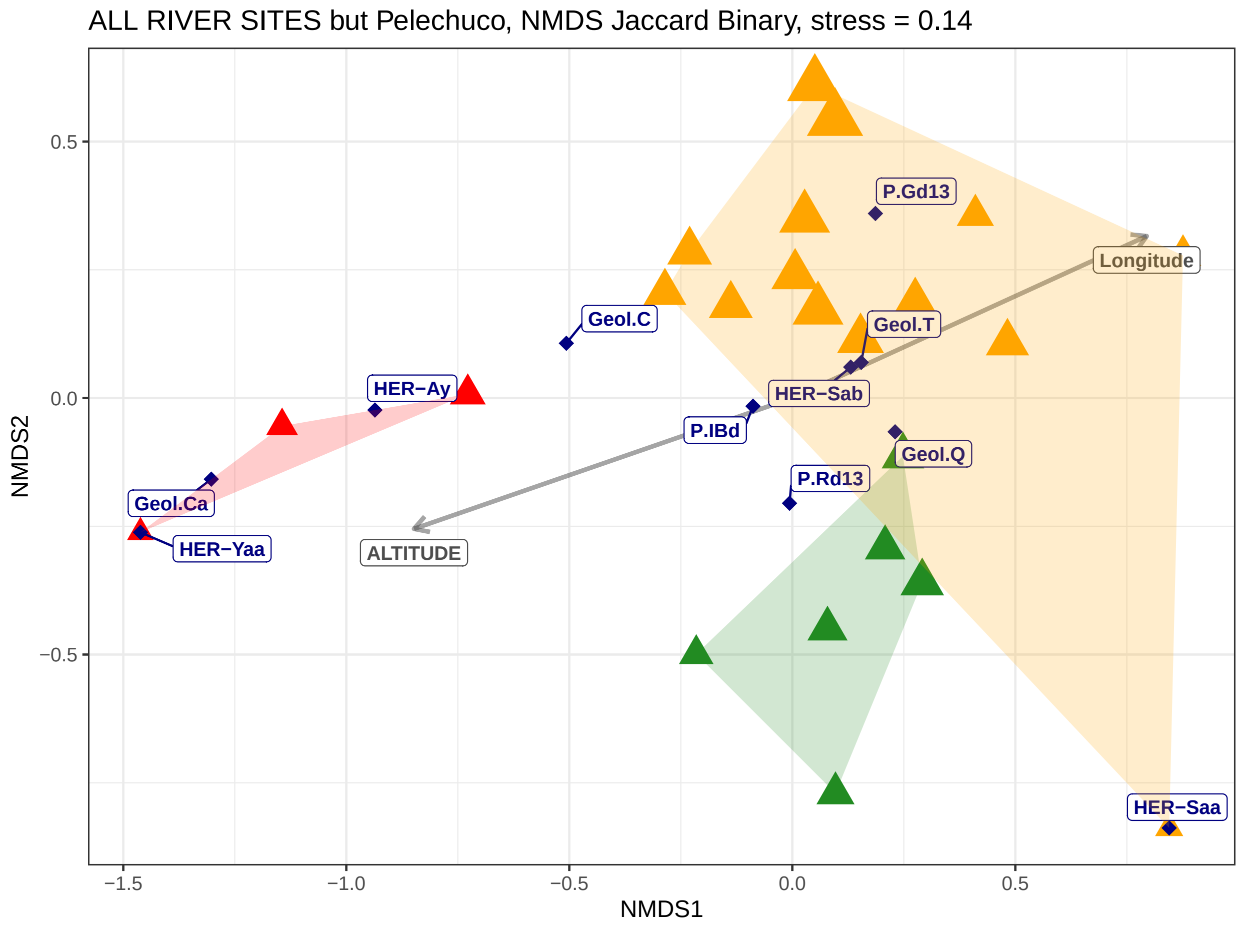

NMDS LAKES Sites (using PHYLOSEQ)

Import data

```
sample_data(physeq5)$SampleID <- sample_data(physeq5)$Site.Name  # add a var named 'SampleID' in sam_da
physeq7 <- subset_samples(physeq5, ECOTYPE == "LAKE")
sample_data(physeq7)$ClusterGroups <- as.character(sample_data(physeq7)$ClusterGroups)
```

Run ordination on the generated distance

```
set.seed(62)
ord <- ordinate(physeq7, "NMDS", distance = "jaccard",
    binary = TRUE, k = 2, maxit = 1500, trymax = 10000,
    wascores = TRUE)
```

Extract scrs

```r
# scores(ord, display = 'sites') scores(ord,
# display = 'species') goodness(ord)
stressplot(ord)
```

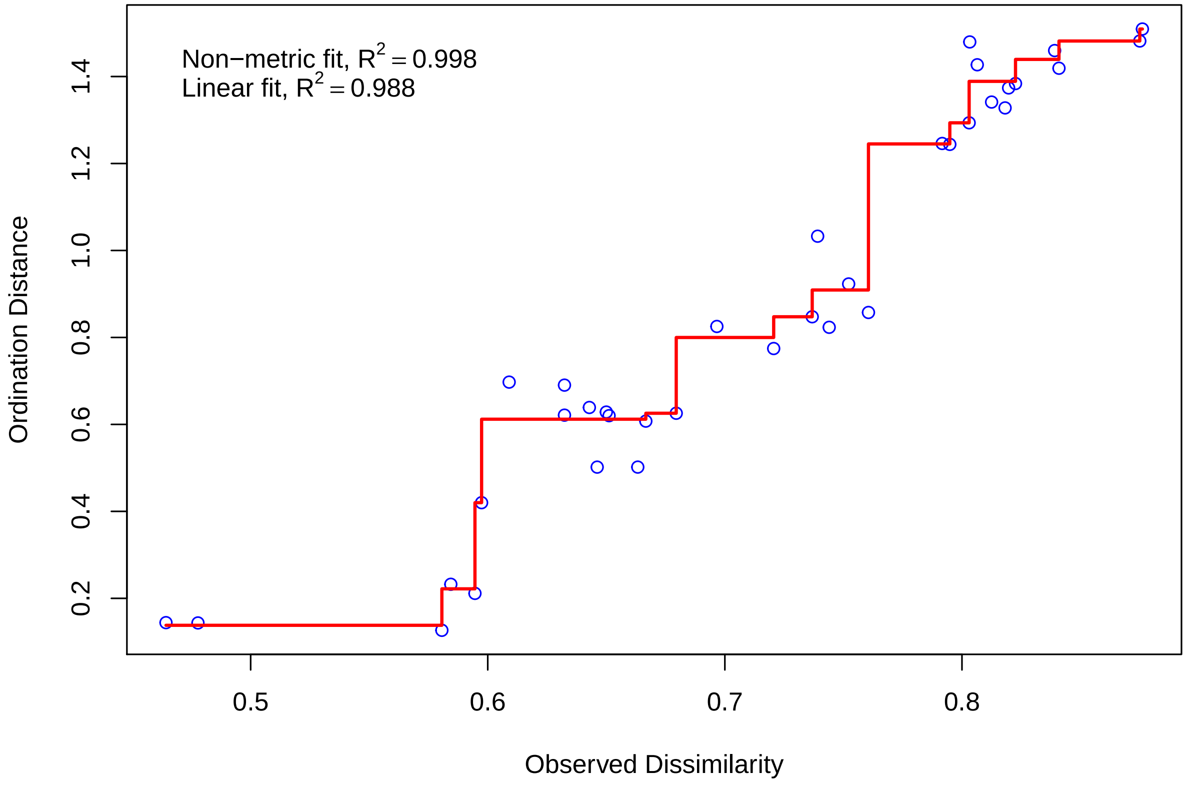


```r
ord$stress
```

```
## [1] 0.04903996
```

Define color vector

```r
col_list = c("dodgerblue")
```

Extract richness for futur point sizing

```r
p <- plot_richness(physeq7, x = "sample", color = "SampleID",
    measures = c("Observed", "Shannon", "ACE"), nrow = 1)
print(p)
```

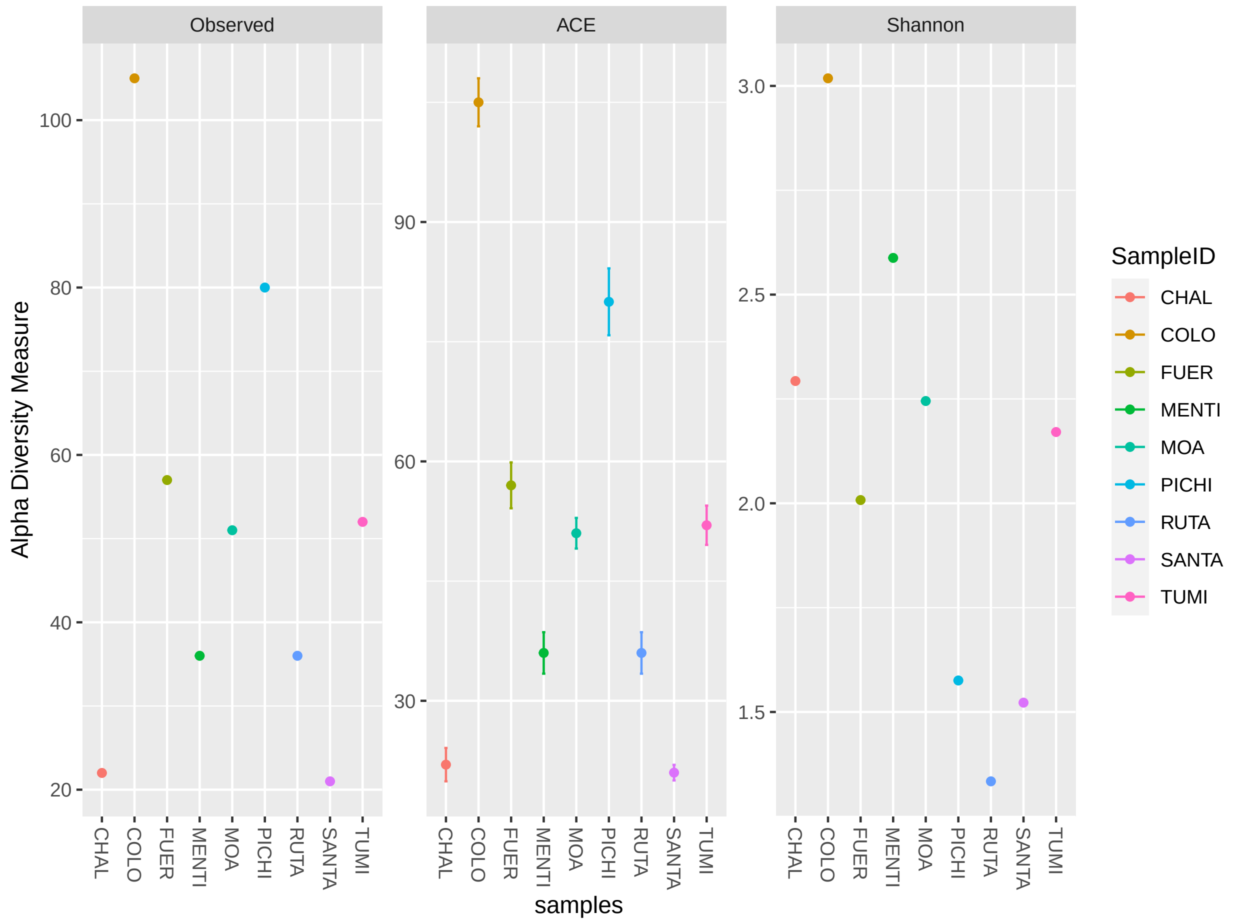


```
Richness <- dcast(p$data, samples + SampleID ~ variable)
```

Preliminary graph

```
p0 <- plot_ordination(physeq7,
                      ord,
                      #color = as.character("AGE_Laguna"),
                      #color = as.character("PEDO"),
                      color="ClusterGroups",
                      #shape= "PEDO",
                      title = "NMDS jaccard binary" ) + #label= "SampleID"
  geom_point(aes(size=Richness$Observed)) +
  theme_bw()
#p0
p0+scale_color_manual(values=c("dodgerblue"))+
  theme_bw() + geom_text_repel(mapping = aes(label = SampleID),size = 3, vjust = 3)
```

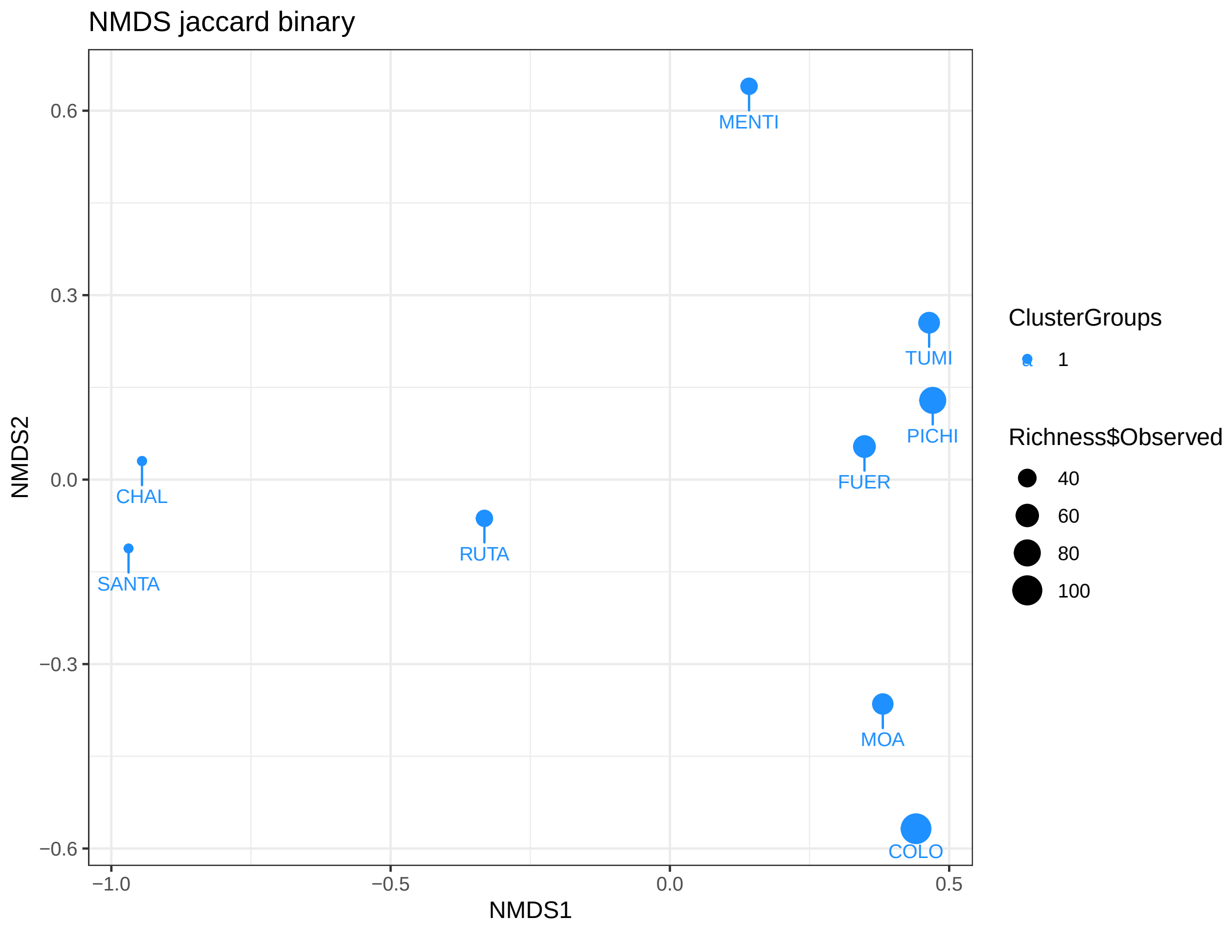

Plot Vegan + surf on hydrologigal network size

```
sampledf <- data.frame(sample_data(physeq7))
set.seed(62)
ord <- ordinate(physeq7, "NMDS", distance = "jaccard",
    binary = TRUE, k = 2, maxit = 1500, trymax = 10000,
    wascores = TRUE)
```

```
plot(ord, display = "sites", type = "n")
points(ord, display = "sites")
# ordipointlabel(ord,display='sites', add=TRUE)
plot(ord$points, col = col_list, pch = 16, type = "n")
points(ord, display = "sites", cex = sampledf$Species/30,
    pch = 16)
ordispider(ord, groups = sampledf$ClusterGroups, label = TRUE,
    scaling = scl, col = col_list)
# ordiellipse(ord,groups=sampledf$clustgroups,
# col=col_list, lwd=2, scaling=scl, draw='polygon'
# ) ordiellipse(ord,groups=sampledf$clustgroups,
# col=col_list, lwd=2, scaling=scl)
surf <- ordisurf(ord ~ sampledf$Red.drenaje..m., data = sampledf,
    knots = 5, isotropic = TRUE, main = NULL, add = TRUE)
```

Search for environmental variables that are significantly correlated with NMDS

Draw significative environmental variables : + PEDO+Longitude+GEOL+GEOL+pH+Salinity+Conductivity+Temperature

```
set.seed(62)
# ord.fit <- envfit(ord ~
# Stability+Saturation+Turbidity+TDS+Temperature+Conductity+Salinity+pH+Oxigene,
# data=as(sample_data(physeq7), 'data.frame'),
# perm=10000, na.rm=TRUE) ord.fit
ord.fit1 <- envfit(ord ~ AGE_Laguna + AREA_ha + PEDO +
    Latitude + ALTITUDE + Longitude + GEOL + AREA_basin_ha +
    Red.drenaje..m. + HydroEcoRegion, data = as(sample_data(physeq7),
    "data.frame"), perm = 10000, na.rm = TRUE)
ord.fit1
```

```
##
## ***VECTORS
##
##                     NMDS1    NMDS2     r2  Pr(>r)
## AGE_Laguna       0.80304  0.59593 0.0367 0.88051
## AREA_ha          0.90455 -0.42636 0.0750 0.81762
## Latitude         0.34246  0.93953 0.6564 0.05329 .
## ALTITUDE        -0.89871 -0.43853 0.9328 0.00030 ***
## Longitude        0.44588  0.89509 0.6623 0.04140 *
## AREA_basin_ha    0.28806 -0.95761 0.6666 0.04660 *
## Red.drenaje..m.  0.28430 -0.95874 0.6691 0.04320 *
## ---
## Signif. codes:  0 '***' 0.001 '**' 0.01 '*' 0.05 '.' 0.1 ' ' 1
## Permutation: free
## Number of permutations: 10000
##
## ***FACTORS:
##
## Centroids:
##                       NMDS1   NMDS2
## PEDOAp14-2/3a        0.4308 -0.2680
## PEDOGd1-3a           0.1555  0.2214
## PEDOI-Bd-Rd-c       -0.9571 -0.0409
## GEOLQuaternary       0.0000  0.0000
## HydroEcoRegionAb-a   0.3029  0.4475
## HydroEcoRegionAb-b   0.4101 -0.1875
## HydroEcoRegionSa-b  -0.7488 -0.0482
##
## Goodness of fit:
##                    r2   Pr(>r)
## PEDO           0.7579 0.005299 **
## GEOL           0.0000 1.000000
## HydroEcoRegion 0.8065 0.002100 **
## ---
## Signif. codes:  0 '***' 0.001 '**' 0.01 '*' 0.05 '.' 0.1 ' ' 1
## Permutation: free
## Number of permutations: 10000
```

Significative var

```r
ord.fit11 <- envfit(ord ~ ALTITUDE + Longitude + P. +
    Red.drenaje..m. + AREA_basin_ha + HydroEcoRegion +
    P., data = as(sample_data(physeq7), "data.frame"),
    perm = 1000, na.rm = TRUE)
ord.fit11
```

```
##
## ***VECTORS
##
##                      NMDS1    NMDS2     r2   Pr(>r)
## ALTITUDE          -0.89871 -0.43853 0.9328 0.002997 **
## Longitude          0.44588  0.89509 0.6623 0.044955 *
## Red.drenaje..m.    0.28430 -0.95874 0.6691 0.028971 *
## AREA_basin_ha      0.28806 -0.95761 0.6666 0.034965 *
## ---
## Signif. codes:  0 '***' 0.001 '**' 0.01 '*' 0.05 '.' 0.1 ' ' 1
## Permutation: free
## Number of permutations: 1000
##
## ***FACTORS:
##
## Centroids:
##                        NMDS1   NMDS2
## P.Ap14                0.4308 -0.2680
## P.Gd13                0.1555  0.2214
## P.IBd                -0.9571 -0.0409
## HydroEcoRegionAb-a    0.3029  0.4475
## HydroEcoRegionAb-b    0.4101 -0.1875
## HydroEcoRegionSa-b   -0.7488 -0.0482
##
## Goodness of fit:
##                    r2   Pr(>r)
## P.             0.7579 0.006993 **
## HydroEcoRegion 0.8065 0.001998 **
## ---
## Signif. codes:  0 '***' 0.001 '**' 0.01 '*' 0.05 '.' 0.1 ' ' 1
## Permutation: free
## Number of permutations: 1000
```

```r
# ord.fit22 <- envfit(ord ~ Turbidity+pH,
# data=as(sample_data(physeq7), 'data.frame'),
# perm=1000, na.rm=TRUE)

data.scores = as.data.frame(scores(ord))
data = as(sample_data(physeq7), "data.frame")
data.scores$ClusterGroups = data$ClusterGroups
data.scores$ECOTYPE = data$ECOTYPE
data.scores$Richness = data$Species
arrowhead = arrow(length = unit(0.02, "npc"))
en_coord_cont1 = as.data.frame(scores(ord.fit11, "vectors"))  #* ordiArrowMul(ord.fit11, fill=1)
```

```
en_coord_cat1 = as.data.frame(scores(ord.fit11, "factors"))  #* ordiArrowMul(ord.fit22, fill=0.1)
# en_coord_cont2 = as.data.frame(scores(ord.fit22,
# 'vectors'))
en_coord_cont <- rbind(en_coord_cont1)  #,en_coord_cont2
rownames(en_coord_cont)[rownames(en_coord_cont) ==
    "Red.drenaje..m."] <- "Network length"
rownames(en_coord_cont)[rownames(en_coord_cont) ==
    "AREA_basin_ha"] <- "Basin Area"

rownames(en_coord_cat1)[rownames(en_coord_cat1) ==
    "HydroEcoRegionAb-a"] <- "HER-Aba"
rownames(en_coord_cat1)[rownames(en_coord_cat1) ==
    "HydroEcoRegionAb-b"] <- "HER-Abb"
rownames(en_coord_cat1)[rownames(en_coord_cat1) ==
    "HydroEcoRegionSa-b"] <- "HER-Sab"
rownames(en_coord_cat1)[rownames(en_coord_cat1) ==
    "HydroEcoRegionAy"] <- "HER-Ay"
rownames(en_coord_cat1)[rownames(en_coord_cat1) ==
    "HydroEcoRegionCg"] <- "HER-Cg"
rownames(en_coord_cat1)[rownames(en_coord_cat1) ==
    "HydroEcoRegionSa-a"] <- "HER-Saa"
rownames(en_coord_cat1)[rownames(en_coord_cat1) ==
    "HydroEcoRegionYa-a"] <- "HER-Yaa"
```

FIGURE 6C : NMDS & environmentales variables LAKE

```
gg = ggplot(data = data.scores, aes(x = NMDS1, y = NMDS2)) +
  geom_point(data = data.scores, aes(colour = ClusterGroups, size=Richness),shape=16, alpha = 1) +
  scale_colour_manual(values = col_list)  +
  scale_size( limits = c(0, 125),breaks =c(20,60,120),range = c(0, 10) )+ #waiver()
  geom_segment(aes(x = 0, y = 0, xend = NMDS1, yend = NMDS2),
               data = en_coord_cont, size =1, alpha = 0.5, colour = "grey30",arrow = arrowhead) +
  geom_point(data = en_coord_cat1, aes(x = NMDS1, y = NMDS2),
             shape = "diamond", size = 3, alpha = 1, colour = "navy") +
  geom_label_repel(data = en_coord_cat1, aes(x = NMDS1, y = NMDS2),force= 10,label.padding= 0.2, size =
                   label = row.names(en_coord_cat1), colour = "navy", fontface = "bold") +
  geom_label_repel(data = en_coord_cont, aes(x = NMDS1, y = NMDS2), colour = "grey30",  size = 3,
                   fontface = "bold", force= 3,label.padding= 0.2,label = row.names(en_coord_cont),vjus
  theme(axis.title = element_text(size = 10, face = "bold", colour = "grey30"),
        panel.background = element_blank(), panel.border = element_rect(fill = NA, colour = "grey30"),
        axis.ticks = element_blank(), axis.text = element_blank(), legend.key = element_blank(),
        legend.title = element_text(size = 10, face = "bold", colour = "grey30"),
        legend.text = element_text(size = 9, colour = "grey30")) +
  labs(colour = "ClusterGroups", title="toto")+
  #geom_polygon(data = hulls,mapping = aes(NMDS1,NMDS2,group=factor(clustgroups),fill=factor(clustgroup
  scale_color_manual(values = col_list) +
  scale_fill_manual(values = col_list)+
  theme_bw()+
  guides( fill=FALSE)+
  labs(size="Richness",title = paste0("LAKES, NMDS Jaccard Binary, stress = ", round(ord$stress,2)))
```

```
# gg + guides(color=guide_legend(title="Biogeographic entities"))
# Remove title for all legends
gg + theme(legend.position="none")
```

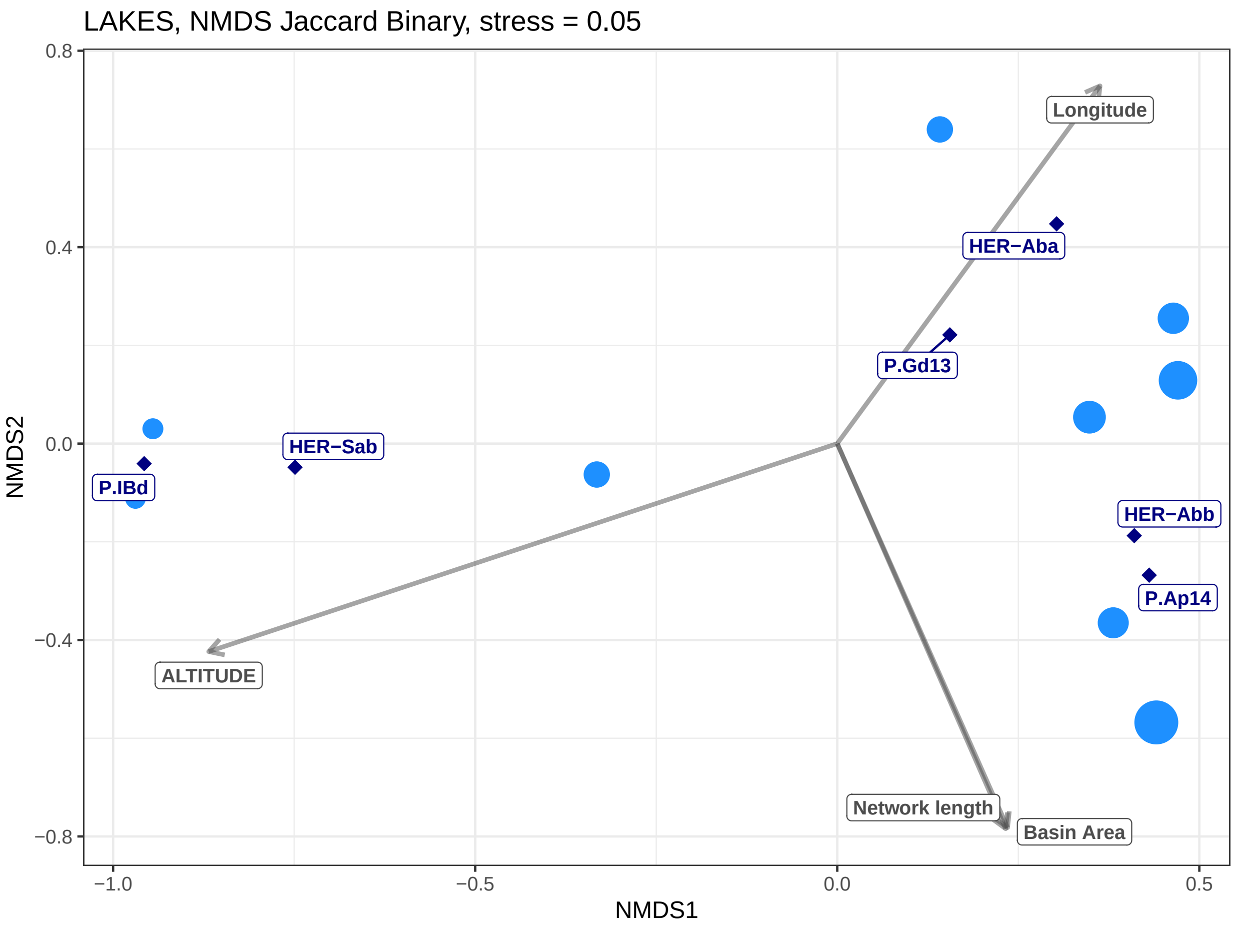

NOT USED : NMDS under PHYLOSEQ subset analysis on RIVERS of RANKIII only

```
physeq7 <- subset_samples(physeq5, River.Rank == "3" &
    SampleID != "MACH")
sample_data(physeq7)$ClusterGroups <- as.character(sample_data(physeq7)$ClusterGroups)
```

Run ordination on the generated distance

```
ord <- ordinate(physeq7, "NMDS", distance = "jaccard",
    binary = TRUE, k = 2, maxit = 1500, trymax = 10000,
    wascores = TRUE)

# ord.fit <- envfit(ord ~
```

```
# Saturation+Turbidity+TDS+Temperature+Conductity+Salinity+pH+Oxigene,
# data=as(sample_data(physeq7), 'data.frame'),
# perm=10000, na.rm=TRUE) ord.fit
ord.fit <- envfit(ord ~ PEDO + Latitude + ALTITUDE +
    Longitude + GEOL + AREA_basin_ha + HydroEcoRegion,
    data = as(sample_data(physeq7), "data.frame"),
    perm = 10000, na.rm = TRUE)
ord.fit
```

Draw significative environmental variables = NONE are significatives

```
# PEDO+Longitude+GEOL+GEOL+pH+Salinity+Conductivity+Temperature+TDS+Longitude+ALTITUDE+HydroEcoRegion
# ord.fit1 <- envfit(ord ~ Temperature ,
# data=as(sample_data(physeq7), 'data.frame'),
# perm=10000, na.rm=TRUE) ord.fit1
# col_list=c('forestgreen') data.scores =
# as.data.frame(scores(ord))
# data=as(sample_data(physeq7), 'data.frame')
# data.scores$cluster = data$clustgroups
# data.scores$ECOTYPE = data$ECOTYPE
# data.scores$SampleID = data$SampleID
# data.scores$Richness =
# data$Number_of_Species_Observed_reads.4.f.0.001
# arrowhead = arrow(length = unit(0.02, 'npc'))
# en_coord_cont = as.data.frame(scores(ord.fit1,
# 'vectors')) * ordiArrowMul(ord.fit1, fill=1.5)
# en_coord_cat = as.data.frame(scores(ord.fit1,
# 'factors')) #* ordiArrowMul(ord.fit1, fill=0.1)
```

FIGURE 6X : NMDS & environmentales variables Rivers Rank3

```
# gg = ggplot(data = data.scores, aes(x = NMDS1, y
# = NMDS2)) + geom_point(data = data.scores,
# aes(colour = cluster, size=Richness),shape=17,
# alpha = 1) + geom_text_repel(aes(label =
# SampleID),size = 3, vjust = 3)+ scale_size(
# limits = c(0, 100),breaks =c(25,50,75,100) )+
# #waiver() scale_colour_manual(values = col_list)
# + geom_segment(aes(x = 0, y = 0, xend = NMDS1,
# yend = NMDS2), data = en_coord_cont, size =1,
# alpha = 0.5, colour = 'grey30',arrow = arrowhead)
# + #geom_point(data = en_coord_cat, aes(x = NMDS1,
# y = NMDS2),shape = 'diamond', size = 3, alpha =
# 1, colour = 'navy') + #geom_label_repel(data =
# en_coord_cat, aes(x = NMDS1, y = NMDS2),force=
# 10,label.padding= 0.2, size = 3, label =
# row.names(en_coord_cat), colour = 'navy',
# fontface = 'bold') + geom_label_repel(data =
# en_coord_cont, aes(x = NMDS1, y = NMDS2), colour
# = 'grey30', size = 3, fontface = 'bold', force=
```

```r
# 1,label.padding= 0.2,label =
# row.names(en_coord_cont), vjust=1.2) + #,
# hjust=1.2 theme(axis.title = element_text(size =
# 10, face = 'bold', colour = 'grey30'),
# panel.background = element_blank(), panel.border
# = element_rect(fill = NA, colour = 'grey30'),
# axis.ticks = element_blank(), axis.text =
# element_blank(), legend.key = element_blank(),
# legend.title = element_text(size = 10, face =
# 'bold', colour = 'grey30'), legend.text =
# element_text(size = 9, colour = 'grey30')) +
# labs(colour = 'cluster')+ #geom_polygon(data =
# hulls,mapping =
# aes(NMDS1,NMDS2,group=factor(clustgroups),fill=factor(clustgroups)),
# alpha = 0.2,inherit.aes = FALSE)+
# scale_color_manual(values = col_list) +
# scale_fill_manual(values = col_list)+ theme_bw()+
# guides( fill=FALSE)+ labs(size='Observed
# richness',title = paste0('RIVERS of rank 3, but
# Machariapo, NMDS Jaccard Binary, stress = ',
# round(ord$stress,2))) gg
```

Plot NMDS ordination Altitude iso lines

```r
library(ggrepel)
```

Input data

```r
physeq7 <- physeq5  # from 10_NMDS-ENVI-VAR.R
sample_data(physeq7)$ClusterGroups <- as.character(sample_data(physeq7)$ClusterGroups)
```

Extract richness for futur point sizing

```r
pr <- plot_richness(physeq7, x = "sample", color = "Site.Name",
    measures = c("Observed", "Shannon", "ACE"), nrow = 1)
Richness <- dcast(pr$data, samples + SampleID ~ variable)
```

NMDS Ordination

```r
ord <- ordinate(physeq7, "NMDS", distance = "jaccard",
    binary = TRUE, k = 2, maxit = 1500, trymax = 1000,
    wascores = TRUE)
# plot
p0 = plot_ordination(physeq7, ord, type = "samples",
```

```r
    color = "ClusterGroups", shape = "ECOTYPE", title = "NMDS jaccard binary",
    label = "SampleID") + geom_point(aes(size = Richness$Observed)) +
    theme_bw() + scale_color_manual(values = c("dodgerblue",
    "red", "forestgreen", "orange"))
```

combine NMDS points and metadata

```r
mite.NMDS.data <- physeq7@sam_data  # do it for ease of plotting
mite.NMDS.data$NMDS1 <- ord$points[, 1]  # this puts the NMDS scores for the plots into a new dataframe
mite.NMDS.data$NMDS2 <- ord$points[, 2]
# mite.NMDS.data
```

Ordisurf

```r
ordi <- ordisurf(ord ~ mite.NMDS.data$ALTITUDE)  # created the ordisurf object
```

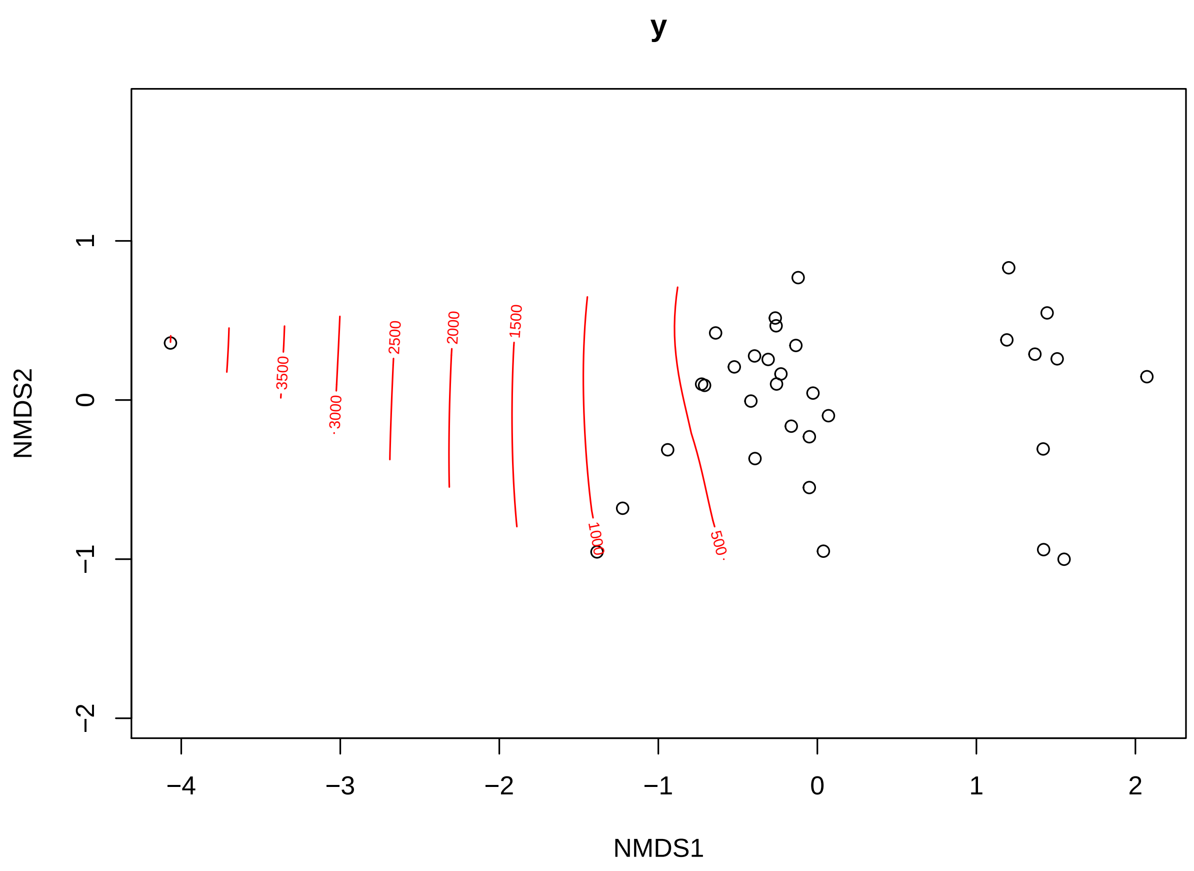


```r
ordi.grid <- ordi$grid  # extracts the ordisurf object
str(ordi.grid)  # it's a list though - cannot be plotted as is
```

```
ordi.mite <- expand.grid(x = ordi.grid$x, y = ordi.grid$y)  # get x and ys
ordi.mite$z <- as.vector(ordi.grid$z)  # unravel the matrix for the z scores
ordi.mite.na <- data.frame(na.omit(ordi.mite))  # gets rid of the nas
# ordi.mite.na #looks ready for plotting!
```

Plotting

```
my_groupmar <- as.numeric(as.factor(mite.NMDS.data$ClusterGroups))
col_vector <- c("dodgerblue", "red", "forestgreen",
    "orange")
colSidemar <- col_vector[my_groupmar]

p <- ggplot(mite.NMDS.data, aes(x = NMDS1, y = NMDS2)) +
    geom_point(alpha = 0.9, aes(shape = ECOTYPE, size = Richness$Observed),
        color = colSidemar) + # color=GEOL,plots the NMDS points, with shape by
# topo type geom_text(aes(label=SampleID),hjust=0,
# vjust=0, cex=2)+
stat_contour(data = ordi.mite.na, aes(x = x, y = y,
    z = z, colour = ..level..), binwidth = 200) + # can change the binwidth depending on how many
# contours you want
theme_bw() + # scale_shape_manual('ECOTYPE', values = c(1,16)) +
# #sets the name of the legend for shape, and says
# which symbols we want (equivalent to the pch
# command in plot)
labs(colour = "ALTITUDE", size = "Richness") + # another way to set the labels, in this case, for
# the colour legend
scale_colour_gradient(high = "darkolivegreen1", low = "darkgreen") +
    # here we set the high and low of the colour scale.
# Can delete to go back to the standard blue, or
# specify others
geom_text_repel(mapping = aes(label = SampleID), size = 3,
    vjust = 3, color = colSidemar) + # geom_label_repel(mapping = aes(label =
# SampleID),size = 3, vjust = 3,color=colSidemar)+
labs(size = "Richness", title = paste0("NMDS Jaccard Binary, stress = ",
    round(ord$stress, 2)))
p
```

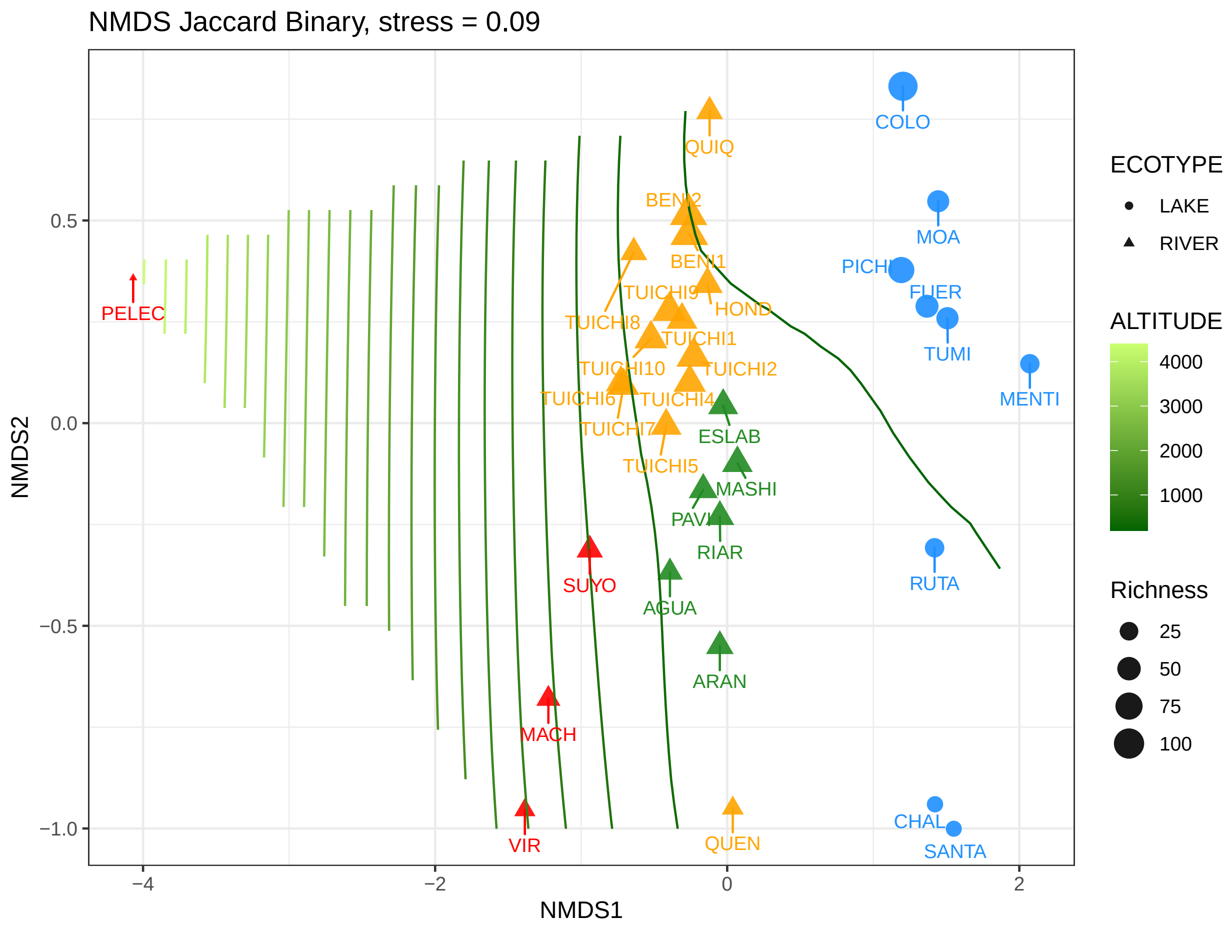

Plot NMDS ordination, Order facet and species contributions

Input data

```
physeq7 <- physeq5  # from 10_NMDS-ENVI-VAR.R
sample_data(physeq7)$ClusterGroups <- as.character(sample_data(physeq7)$ClusterGroups)
sampledf <- data.frame(sample_data(physeq7))  # extract metadata in dataframe
otudf <- data.frame(otu_table(physeq7))  # extract OTU in dataframe
col_list = c("dodgerblue", "red", "forestgreen", "orange")
```

Plotting bi-plot in ordination space

```
ord <- ordinate(physeq7, "NMDS", distance = "jaccard",
    binary = TRUE, k = 2, maxit = 1500, trymax = 1000,
    wascores = TRUE)
plot(ord)
orditorp(ord, "sites", col = "blue")  # Gives points labels plot(ord, 'species')   # Plots species scor
orditorp(ord, "species", col = "red", pch = 3, air = 0.011)
```

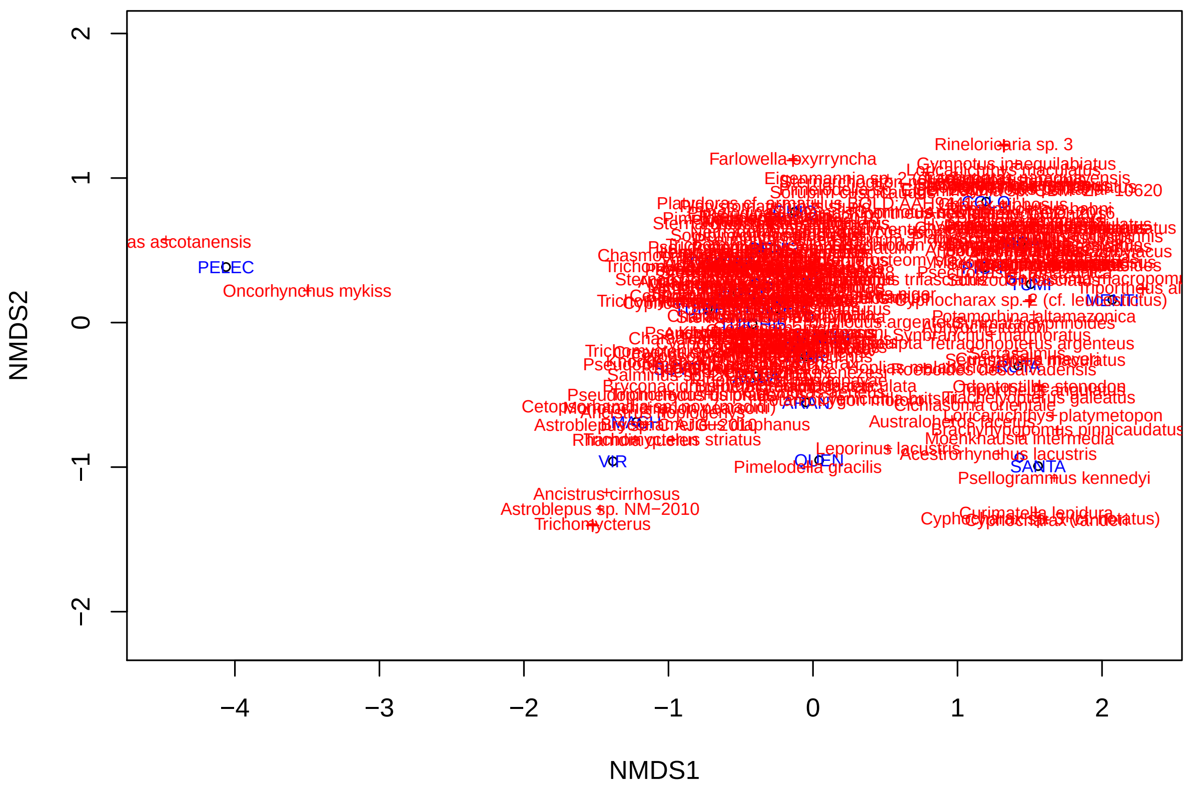


```
p0 <- plot_ordination(physeq7, ord, color = "ClusterGroups",
    shape = "ECOTYPE", title = "NMDS jaccard binary")
```

SFIGURE3

```
# For drawing polygons : keep external points
find_hull <- function(hull.data) hull.data[chull(hull.data$NMDS1, hull.data$NMDS2), ]
hull.data <- merge(scores(ord, display = "sites"), sampledf,by = 0)
hulls <- ddply(hull.data, "ClusterGroups", find_hull)

p01<- p0 + geom_polygon(data = hulls,mapping = aes(NMDS1,NMDS2,group=factor(ClusterGroups),fill=factor(C
  scale_color_manual(values = col_list) +
  scale_fill_manual(values = col_list)

p1 = plot_ordination(physeq7, ord,type="taxa", title = "NMDS jaccard binary")+ # ,color = "Order", labe
   geom_polygon(data = hulls,mapping = aes(NMDS1,NMDS2,group=factor(ClusterGroups),fill=factor(ClusterGr
                , alpha = 0.2,inherit.aes = FALSE)+
   scale_fill_manual(values = col_list)

p1 + facet_wrap(~Order, 3)+
  geom_polygon(data = hulls,mapping = aes(NMDS1,NMDS2,group=factor(ClusterGroups),fill=factor(ClusterGro
  theme_bw()
```

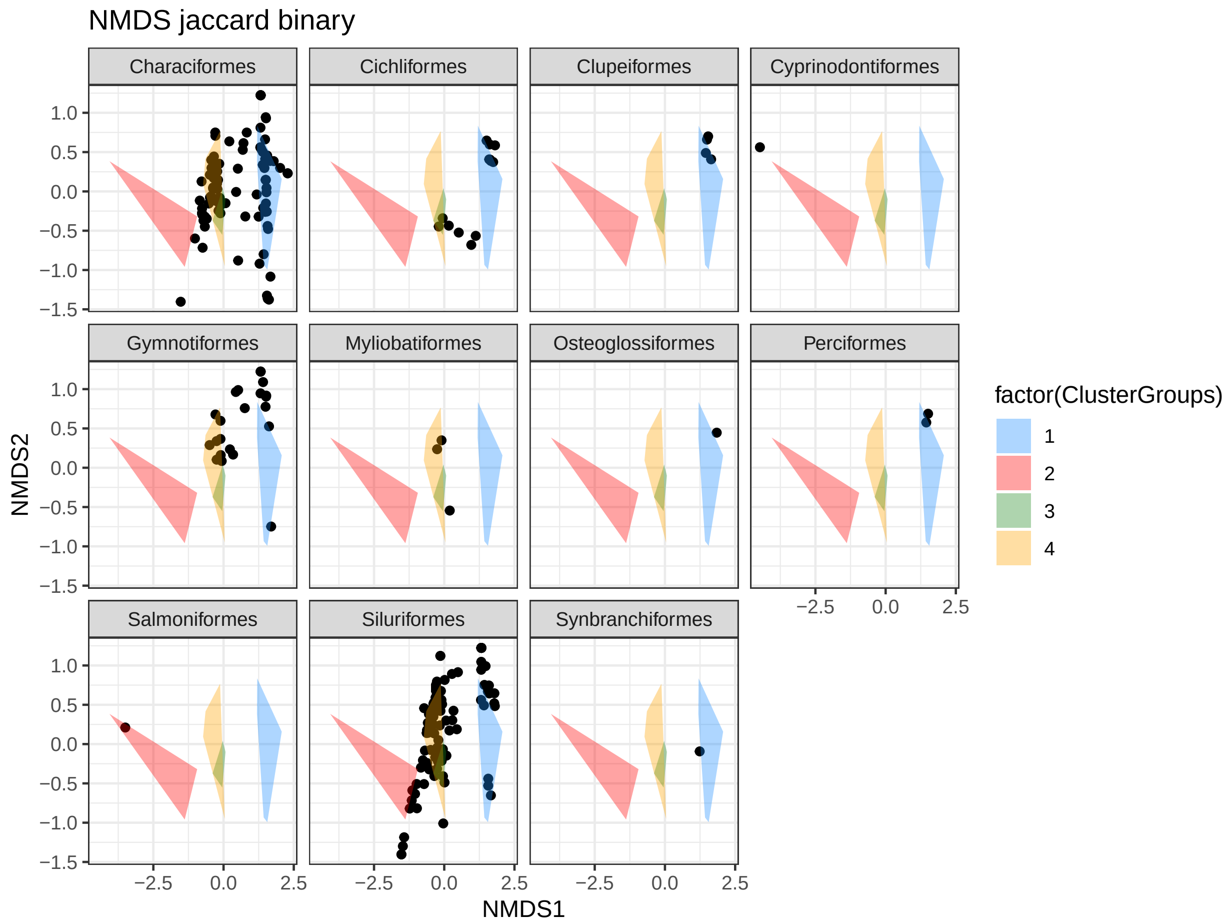

corelation between var

```
setwd("C:/Users/mariac/Documents/Documents/ACEDRIC/Programmes/PIMELO-et-ECOBIO/E-DNA manipes/EDNA-2001il

corela <- read.xlsx("SUPP DATA Tables and SFigures.xlsx",
    sheet = "STable3 metad. site level", rowNames = F,
    colNames = TRUE, rows = c(2:35), cols = c(1:29))

res <- cor.test(log(corela$ALTITUDE), log(corela$Temperature),
    method = "pearson")
plot(corela$ALTITUDE ~ corela$Temperature)
res
summary(corela)
library(pastecs)
stat.desc(corela)
```

Compare % of Characiformes / Siluriformes in Lakes and Rivers

Data

	Characiformes	Autres
LAKES	61	92
RIVERS	56	116

p-value = 0.1705

	Siluriformes	Autres
LAKES	49	103
RIVERS	90	82

p-value = 0.0002663

Import data and test

copy and paste table in excel, then copy

```
test_percent <- read.table("clipboard", sep = "\t",
    header = T, row.names = 1)
## Pearson's Chi-squared test
chisq.test(test_percent, correct = FALSE)
```

ADONIS : test for cluster differences in ordination plot

```
library(vegan)
set.seed(62)  # for reproduciblity
```

Input data (Phyloeq object)

```
physeq77 = physeq5
sample_data(physeq77)$ClusterGroups <- as.character(sample_data(physeq77)$ClusterGroups)
```

Export OTU and Metadata in data.frame

```
sampledf <- data.frame(sample_data(physeq77))  # extract metadata in dataframe
otudf <- data.frame(otu_table(physeq77))  # extract OTU in dataframe
```

Ordination Using phyloseq

```r
ord2 <- ordinate(physeq77, "NMDS", distance = "jaccard",
    binary = TRUE, k = 2, maxit = 1500, trymax = 1000,
    wascores = TRUE, autotransform = TRUE, center = TRUE)
```

Plot ordination ellipses, spider, ordisurf

```r
col_list = c("dodgerblue", "red", "forestgreen", "orange")
plot(ord2$points, col = col_list[sampledf$ClusterGroups],
    pch = 16)

ordipointlabel(ord, display = "sites", add = TRUE,
    cex = 0.7)
# ordipointlabel(ord,display='sites', add=TRUE,
# cex=0.7)
ordispider(ord2, groups = sampledf$ClusterGroups, label = T,
    scaling = scl, col = col_list)
ordiellipse(ord2, groups = sampledf$ClusterGroups,
    col = col_list, lwd = 2, scaling = scl, draw = "polygon")
ordiellipse(ord2, groups = sampledf$ClusterGroups,
    col = col_list, lwd = 2, scaling = scl)

points(ord2, display = "sites", cex = 0.5, pch = 16,
    col = col_list)
```

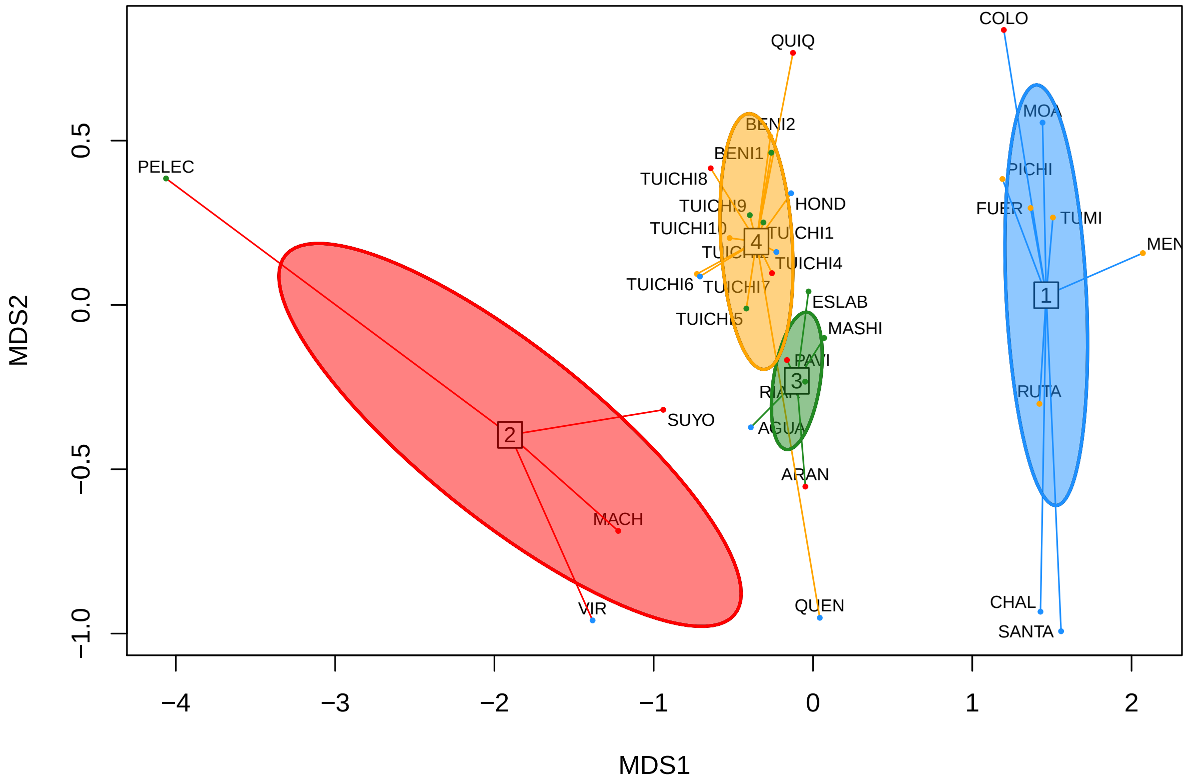


```r
# surf <- ordisurf(ord2 ~ sampledf$ALTITUDE,
# data=sampledf, knots=10, isotropic=TRUE,
# main=NULL, add=TRUE)
```

test for differences in community among the clustergroups

```r
# this transformation was done during metaMDS, see
# ord In this standardization, each element is
# divided by its column maximum and then divided by
# the row total
otusdt2 <- wisconsin(sqrt(otudf))
```

Adonis on OTU table directly

```r
adonis(otusdt2 ~ ClusterGroups, method = "jaccard",
    binary = "TRUE", data = sampledf, permutations = 1000)
```

```
##
## Call:
```

```
## adonis(formula = otusdt2 ~ ClusterGroups, data = sampledf, permutations = 1000,      method = "jacca
##
## Permutation: free
## Number of permutations: 1000
##
## Terms added sequentially (first to last)
##
##               Df SumsOfSqs MeanSqs F.Model      R2   Pr(>F)
## ClusterGroups  3    3.9607 1.32023  6.1806 0.39001 0.000999 ***
## Residuals     29    6.1946 0.21361         0.60999
## Total         32   10.1553                 1.00000
## ---
## Signif. codes:  0 '***' 0.001 '**' 0.01 '*' 0.05 '.' 0.1 ' ' 1
```

```r
# adonis(otudf~ClusterGroups,method='jaccard',binary='TRUE',
# data=sampledf, permutations = 1000)
```

Adonis on distance matrix

```r
jacc1 <- vegan::vegdist(otusdt2, "jaccard", binary = T)  # similar with : jacc0 <- phyloseq::distance(p
# adonis(jacc1~jacc1, data=sampledf, permutations =
# 1000)
```

p=0.000999

Homogeneity of dispersion test (critical assumption here is that variance is egal among the clustgroups)

using anova

```r
anova(betadisper(jacc1, sampledf$ClusterGroups))
```

```
## Analysis of Variance Table
##
## Response: Distances
##           Df  Sum Sq  Mean Sq F value Pr(>F)
## Groups     3 0.05358 0.017860  1.3226 0.2861
## Residuals 29 0.39161 0.013504
```

p=0.2861
Thus the dispersion between these groups is similar

Using permanova

```r
permutest(betadisper(jacc1, sampledf$ClusterGroups))
```

```
##
## Permutation test for homogeneity of multivariate dispersions
## Permutation: free
## Number of permutations: 999
##
## Response: Distances
##           Df  Sum Sq  Mean Sq      F N.Perm Pr(>F)
## Groups     3 0.05358 0.017860 1.3226    999  0.285
## Residuals 29 0.39161 0.013504
```

Annexe1: FROGS sample file script (slurm)

```
#!/bin/bash
frogs_dir="/home/mariac/frogs/FROGS/" # pour fichier pynast
#samplefile="/home/mariac/frogs-R72-11122018/Classeur.tsv" ####### A MODIFIER/VERIFIER
samplefile="./Classeur.tsv"
db="/usr/local/frogs_databases-2.01/silva_123_16S/silva_123_16S.fasta"  #######
nb_cpu=4
java_mem=20
# from user
minAmpliconSize=$1
maxAmpliconSize=$2
fivePrimPrimer=$3
threePrimPrimer=$4
R1size=$5
R2size=$6
expectedAmpliconSize=$7
out_dir=$8
datasetTarGz=$9
# attention a l'orientation du primer 3' a placer en 5' 3' non rev comp
# qsub -q bioinfo.q -N frogsCL -b yes -V -cwd -pe ompi 4 '/home/mariac/frogs/run_frogs_pipeline.sh 110 
##  --min-sample-presence 1 \ : nombre d'echantillon dans lesquels doivent au mini etre retrouvé ce clu
#TODO: ne marche pas s'il n'y a pas les seq des amorces utilisées pour l'amplicon (methode )

# Check parameters
if [ "$#" -ne 9 ]; then
    echo "ERROR: Illegal number of parameters." ;
    echo 'Command usage: run_frogs_pipeline.sh <minAmpliconSize> <maxAmpliconSize> <fivePrimPrimer> <thi
    #echo 'Command usage: run_frogs_pipeline <FROGS_FOLDER> <NB_CPU> <JAVA_MEM> <OUT_FOLDER> <datasetTa
    exit 1 ;
fi
echo $minAmpliconSize;
echo $maxAmpliconSize;
echo $fivePrimPrimer;
echo $threePrimPrimer;
echo $R1size;
echo $R2size;
echo $expectedAmpliconSize;
echo $out_dir;
echo $datasetTarGz;
```

```
# Set ENV
#export PATH=$frogs_dir/libexec:$frogs_dir/app:$PATH
#export PYTHONPATH=$frogs_dir/lib:$PYTHONPATH
module purge
# module load bioinfo/FROGS/2.01
module load bioinfo/FROGS/3.1
source activate frogs

# Create output folder
if [ ! -d "$out_dir" ]
then
    mkdir $out_dir
fi

echo "Step preprocess `date`"

/home/mariac/frogs/preprocess.py illumina \
 --min-amplicon-size $minAmpliconSize --max-amplicon-size $maxAmpliconSize \
 --five-prim-primer $fivePrimPrimer --three-prim-primer $threePrimPrimer \
 --R1-size $R1size --R2-size $R2size --expected-amplicon-size $expectedAmpliconSize \
 --input-archive $datasetTarGz \
 --output-dereplicated $out_dir/01-prepro.fasta \
 --output-count $out_dir/01-prepro.tsv \
 --summary $out_dir/01-prepro.html \
 --log-file $out_dir/01-prepro.log \
 --nb-cpus $nb_cpu --mismatch-rate 0.1

if [ $? -ne 0 ]
then
    echo "Error in preprocess" >&2
    exit 1;
fi

echo "Step clustering `date`"

clustering.py \
 --distance 1 \
 --input-fasta $out_dir/01-prepro.fasta \
 --input-count $out_dir/01-prepro.tsv \
 --output-biom $out_dir/02-clustering.biom \
 --output-fasta $out_dir/02-clustering.fasta \
 --output-compo $out_dir/02-clustering_compo.tsv \
 --log-file $out_dir/02-clustering.log \
 --nb-cpus $nb_cpu

#clustering.py \
# --distance 1 \
# --denoising \
# --input-fasta $out_dir/01-prepro.fasta \
# --input-count $out_dir/01-prepro.tsv \
# --output-biom $out_dir/02-clustering.biom \
# --output-fasta $out_dir/02-clustering.fasta \
# --output-compo $out_dir/02-clustering_compo.tsv \
```

```
# --log-file $out_dir/02-clustering.log \
# --nb-cpus $nb_cpu

if [ $? -ne 0 ]
then
    echo "Error in clustering" >&2
    exit 1;
fi

echo "Step remove_chimera `date`"

remove_chimera.py \
 --input-fasta $out_dir/02-clustering.fasta \
 --input-biom $out_dir/02-clustering.biom \
 --non-chimera $out_dir/03-chimera.fasta \
 --out-abundance $out_dir/03-chimera.biom \
 --summary $out_dir/03-chimera.html \
 --log-file $out_dir/03-chimera.log \
 --nb-cpus $nb_cpu

if [ $? -ne 0 ]
then
    echo "Error in remove_chimera" >&2
    exit 1;
fi

echo "Step filters `date`"

filters.py \
 --min-abundance 0.000000000001 \
 --min-sample-presence 1 \
 --input-biom $out_dir/03-chimera.biom \
 --input-fasta $out_dir/03-chimera.fasta \
 --output-fasta $out_dir/04-filters.fasta \
 --output-biom $out_dir/04-filters.biom \
 --excluded $out_dir/04-filters.excluded \
 --summary $out_dir/04-filters.html \
 --log-file $out_dir/04-filters.log

if [ $? -ne 0 ]
then
    echo "Error in filters" >&2
    exit 1;
fi

echo "Step clusters_stat `date`"

clusters_stat.py \
 --input-biom $out_dir/04-affiliation.biom \
 --output-file $out_dir/05-clustersStat.html \
 --log-file $out_dir/05-clustersStat.log

if [ $? -ne 0 ]
```

```
then
    echo "Error in clusters_stat" >&2
    exit 1;
fi

echo "Step biom_to_tsv `date`"

biom_to_tsv.py \
 --input-biom $out_dir/04-affiliation.biom \
 --input-fasta $out_dir/04-filters.fasta \
 --output-tsv $out_dir/07-biom2tsv.tsv \
 --output-multi-affi $out_dir/07-biom2tsv.multi \
 --log-file $out_dir/07-biom2tsv.log

if [ $? -ne 0 ]
then
    echo "Error in biom_to_tsv" >&2
    exit 1;
fi

echo "Step biom_to_stdBiom `date`"
biom_to_stdBiom.py \
 --input-biom $out_dir/04-affiliation.biom \
 --output-biom $out_dir/08-affiliation_std.biom \
 --output-metadata $out_dir/08-affiliation_multihit.tsv \
 --log-file $out_dir/08-biom2stdbiom.log

if [ $? -ne 0 ]
then
    echo "Error in biom_to_stdBiom" >&2
    exit 1;
fi
echo "Step tsv_to_biom `date`"
tsv_to_biom.py \
 --input-tsv $out_dir/07-biom2tsv.tsv \
 --input-multi-affi $out_dir/07-biom2tsv.multi \
 --output-biom $out_dir/09-tsv2biom.biom \
 --output-fasta $out_dir/09-tsv2biom.fasta \
 --log-file $out_dir/09-tsv2biom.log

if [ $? -ne 0 ]
then
    echo "Error in tsv_to_biom" >&2
    exit 1;
fi
echo "Completed with success"
#on desactive l'environement FROGS
source deactivate
```

Annexe2: BLASTn sample file script (slurm)

```
#!/bin/sh
## Give a name to  your job
#SBATCH --job-name=Blastn
## precise the logfile for your job
#SBATCH --output=Blastn.out
## precise the error file for your job
#SBATCH --error=Blastn.out
# Precise the partion you want to use
#SBATCH --partition=highmem
# precise when you receive the email
#SBATCH --mail-type=end
# precise to  which address you have to send the mail to
#SBATCH --mail-user=cedric.mariac@ird.fr
# Only 1 hour wall-clock time will be given to this job
#SBATCH -t 0-12:00:00
#SBATCH --mem 100GB
# number of cpu you want to use on you node
#SBATCH --cpus-per-task=8

path_to_data="/home/mariac/EDNA/MK1-200lib-min3/";
path_to_scratch="/scratch/mariac_$SLURM_JOB_ID";
path_to_dest="/home/mariac/EDNA/MK1-200lib-min3";

############# chargement du module
module load bioinfo/blast/2.8.1+


###### Creation du repertoire temporaire sur  la partition /scratch du noeud
mkdir $path_to_scratch;

####### copie du repertoire de données  vers la partition /scratch du noeud
scp -r nas:$path_to_data $path_to_scratch; # choisir nas pour/home, /data2 et /teams ou nas2 pour /data
echo "tranfert donnees master -> noeud";
cd $path_to_scratch/MK1-200lib-min3/;

###### Execution des programmes
blastn -db /data/projects/banks/nt_v4/nt -query 04-filters-MK1-200lib-min3.fasta -out blast-ncbi-MK1-20

##### Transfert des données du noeud vers master
scp -rp $path_to_scratch/MK1-200lib-min3/*.out  nas:$path_to_dest/;
echo "Transfert donnees node -> master";

#### Suppression du repertoire tmp noeud
rm -rf $path_to_tmp;
echo "Suppression des donnees sur le noeud";
```
