# Supplementary material for: Unveiling biogeographical patterns of the ichthyofauna in the Tuichi basin, a biodiversity hotspot in the Bolivian Amazon, using environmental DNA
Source: PLoS One. 2022 Jan 4;17(1):e0262357. doi: 10.1371/journal.pone.0262357 (PMC8726463; doi:10.1371/journal.pone.0262357)
Supplement: S2 Text — (PDF) [file pone.0262357.s008.pdf]

Text S2 : Barcode taxonomic resolution

In order to estimate the taxonomic resolution of MK1-185 bp and MK2-285 bp COI barcodes used in this study, an in silico analysis was first performed. We extracted all sequences of Amazonian species available in our COI database (Table S2), if they covered at least 90% of our MK1 and MK2 barcodes.

The MK1 dataset (503 species) is made up of 14 different orders, 46 families and 234 genera and MK2 dataset (543 species) is made up of 14 different orders, 46 families and 246 genera. These sequences were trimmed to remove the region corresponding to the primers thus generating fragments of 130 and 229 bp for MK1 and MK2, respectively.

These MK1 and MK2 sequence datasets were blasted against our COI database which contain 160,944 entries representing 3,480 genera and 15,429 fish species distributed worldwide.

Blastn results were parsed with MEGAN using a minimum identity parameter of 97% and a minimum score value of 100. Under these conditions, if a sequence is assigned to more than one reference in the database, the LCA (lowest common ancestor) is retained.

Among the 503 references extracted on the MK1 barcode, 428 were correctly assigned at the species level, 56 have been assigned to the correct genus and 19 to a higher taxonomic level than the genus.

Among the 543 references extracted on the MK2 barcode, 484 were correctly assigned at the species level, 39 have been assigned to the correct genus and 20 to a higher taxonomic level than the genus.

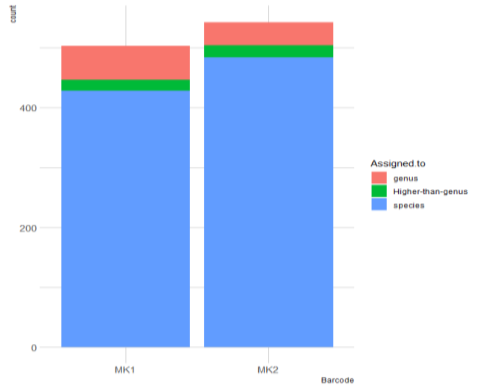

Barplot representing for each COI barcode used in this study (MK1 and MK2) the number of Amazonian fish sequences (extracted from reference database) which are reassigned (Blastn) at the species, genus or a higher taxonomic level. Taxonomic resolution at the species level was 85% and 89% for MK1 and MK2, respectively.

Moreover, as presented in the results, the proportion of OTUs assigned to the species level, which reached 90.5 and 92.5% for MK1 and MK2, respectively, confirm their high taxonomic resolution.
