# Supplementary material for: Unveiling biogeographical patterns of the ichthyofauna in the Tuichi basin, a biodiversity hotspot in the Bolivian Amazon, using environmental DNA
Source: PLoS One. 2022 Jan 4;17(1):e0262357. doi: 10.1371/journal.pone.0262357 (PMC8726463; doi:10.1371/journal.pone.0262357)
Supplement: Attachment — Submitted filename: Comments_to_the_authors.pdf [file pone.0262357.s016.pdf]

Review

Comments to the Author

Environmental DNA (eDNA) approaches have been revolutionizing the assessment and monitoring of biodiversity in both aquatic and terrestrial ecosystems, yet their applications to rich biodiversity sites, especially in the Neotropical region, have largely been limited despite their potential. In this study, the authors assessed the potential of eDNA approaches for monitoring Neotropical fishes by analyzing eDNA from water samples collected both in rivers and lakes in the Bolivian Amazon region. Authors amplified DNA using a newly designed two COI metabarcoding primers targeting Amazon fishes and sequenced the amplicons on Novaseq illumina platform. Thus, they detected a high fish species richness and described their biogeographical distribution patterns related it to a set of ecological descriptors.

The topic of this manuscript fits to the scope of PlosOne, and it is timely and may potentially attract readers in both the field of Ichthyology and of biodiversity assessment using eDNA techniques. However, I found several points (detailed below), especially in the Method section that the authors should address to improve the clarity of the manuscript.

Abstract

Abstract is over the 300 words limit.

Although authors should explain how the study was done without methodological detail, it would greatly strengthen the manuscript to inform in the Abstract the use of designed primers targeting Amazon fishes.

Introduction

L60-61: "The Tuichi basin is included in the Madidi National Park and Natural Area of Integrated Management (hereafter abbreviated Madidi NP), **a recognised biodiversity hotspot [8–10]** and also likely hosts one of the few identified spawning area for many fish species [11]"

It is not recognized among the world's 36 **hotspots** biodiversity. If it is only locally/nationally recognized I would suggest replacing the term "*biodiversity hotspot*" to a "*rich biodiversity site*".

L68-86: Here authors include a brief review of the use of eDNA techniques including examples on amphibians, aquatic mammals, and fishes. However, they failed to current state of eDNA research in the Amazon region. I strongly suggest that authors strengthen this part. A key recent literature on the Amazon fish (Jackman et al. 2021) and mammals (Sales et al. 2019) might help.

L92-94: I would state specifically what your objectives were. - Why did you do this using eDNA? What were your objectives? The aim of the study as it is, seems to be a report case. Make clear the main aims of the study and how significant they are for the field.

Methods

What is the average distance between the 34 collection sites?

Did authors collect eDNA samples across years (2017, 2018, 2019) in the same 34 sites?

L129-130: Include the License/authorization Number. Permits and approvals obtained for the work, including the full name of the authority that approved the study; if none were obtained, authors should explain why.

eDNA field sampling

Were temperature and pH recorded at each site?

Inform depth of sample water collection.

Detailed information on the field and laboratory negative controls are missing. Make clear what is empty control. Please describe what was the field, “alien” and laboratory negative controls (commercial water, distilled water, or ultrapure water?). Additionally, please describe at which site and at which timing the field negative controls were collected and filtered. Also, detailed information on the sequencing platform is also required. I would guess that you used Illumina NovaSeq 6000, but please make it clear.

Did authors validate the designed primers and laboratory protocols before eDNA extraction?

How did authors verify taxonomic resolution of COI fragments targeted by the designed primers? Did they use a database of Neotropical fishes to improve taxonomic assignments? With how many species?

Results

303-307: It is not clear how authors discarded the four taxa.

The accession number AMNHI782-12 (*Marcusenius monteiri)* was not found in GenBank. Make sure it is correct. And the other three taxa have their information matched in both GenBank and Boldsystem (but *Epinephelus marginatus* JX124778.1 was found in Bolsystem by its voucher LBPV53018).

How many reads do those four discarded taxa have? How is the author sure they were not putative contamination?

L315-316: Did authors remove the maximum number of reads found in the controls from all samples before downstream the analysis to avoid putative contamination?

L323-324: It is a very low threshold to ensure non-contamination. Most eDNA studies remove all MTOUS <10 reads from the final dataset. Do authors have a citation for why 4 reads were adopted or just type a further clear justification.

L332-333: One of the cost-efficiency of using eDNA is to detect rare or cryptic species that are difficult to detect by using traditional methods. However, it is highly prone to putative contamination. Since key information on the Methods section is missing, I am not truly convinced that these 16 named *affinis* are not putative contamination. How many reads do those taxa have? Is it possible they have been introduced in the Amazon basin?

Discussion

Authors should put their results in the context with respect to other eDNA studies on fish surveys in the Amazon and Neotropical region. It would greatly strengthen the discussion to briefly detail the survey success obtained in this study to the success of other studies.

Authors should discuss in more detail the conservation issues regarding the species found in the study site, especially regarding the newly detected species.

Are there any threatened species? Is it possible for any of those *affins* species to be an exotic species introduced in the area? *Prochilodus argenteus*, for example, is an invasive species in several Brazilian river basins. Is it possible for the species to be present in the study site?

English

The level of English is very high throughout the proposal, but there are some minor errors throughout. It would therefore be worthwhile having the final version carefully reviewed before final submission.

Figure 3: see comments in the pdf file.

Figure S3: Taxonomic diversity at Order level per site: Axis is very difficult to read. I suggest increasing the text size, or include this figure in an entire page.

Minor comments are in the main text pdf file. Good luck revising the manuscript and I look forward to seeing the revised version!
