# Supplementary material for: Unveiling biogeographical patterns of the ichthyofauna in the Tuichi basin, a biodiversity hotspot in the Bolivian Amazon, using environmental DNA
Source: PLoS One. 2022 Jan 4;17(1):e0262357. doi: 10.1371/journal.pone.0262357 (PMC8726463; doi:10.1371/journal.pone.0262357)
Supplement: Attachment — Submitted filename: Figure_3.pdf [file pone.0262357.s018.pdf]

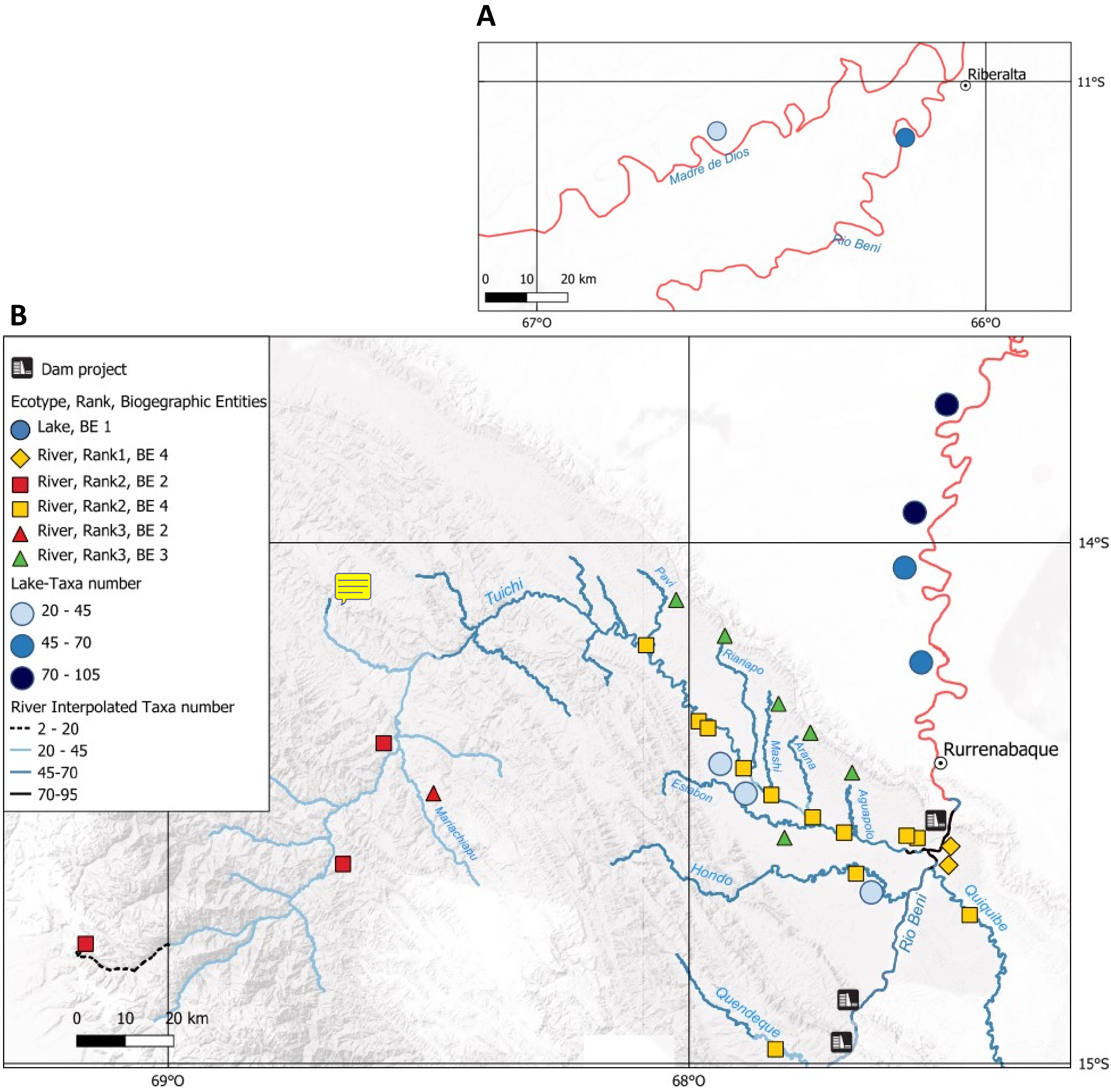
A
Riberalta
11°S
Madre de Dios
Rio Beni
0
10
20 km
67°O
66°O
B
Dam project
Ecotype, Rank, Biogegraphic Entities
Lake, BE 1
River, Rank1, BE 4
River, Rank2, BE 2
River, Rank2, BE 4
River, Rank3, BE 2
River, Rank3, BE 3
Lake-Taxa number
20 - 45
45 - 70
70 - 105
River Interpolated Taxa number
2 - 20
20 - 45
45-70
70-95
Tuichi
Pavi
Riariapo
Mashi
Arana
Aguapolo
Esmbon
Mariachiapu
Hondo
Rio Beni
Quiquibe
Quendeque
Rurrenabaque
14°S
15°S
0
10
20 km
69°O
68°O
