# Supplementary material for: Unveiling biogeographical patterns of the ichthyofauna in the Tuichi basin, a biodiversity hotspot in the Bolivian Amazon, using environmental DNA
Source: PLoS One. 2022 Jan 4;17(1):e0262357. doi: 10.1371/journal.pone.0262357 (PMC8726463; doi:10.1371/journal.pone.0262357)
Supplement: Attachment — Submitted filename: Manuscript-eDNA-16062021.docx [file pone.0262357.s017.docx]

**Unveiling biogeographical patterns of the ichthyofauna in the Tuichi basin, a biodiversity hotspot in the Bolivian Amazon, using environmental DNA.**

Cédric Mariac^1,2,^ †^,^, Guido Miranda^2,3,4,^ ‡, Fabrice Duponchelle^2,5,^ ‡, Camila Ramallo^3^, Robert Wallace^3^, Gabriel Tarifa^3^, Carmen Garcia-Davila^2,6^, Hernán Ortega^7^, Julio Pinto^4^, Jean-François Renno^1,2^

^1^ DIADE, Univ Montpellier, CIRAD, IRD, Montpellier, France.

^2^ Laboratoire Mixte International - Evolution et Domestication de l’Ichtyofaune Amazonienne (LMI - EDIA), IIAP, UAGRM, IRD.

^3^ Wildlife Conservation Society, Bolivia Program, La Paz, Bolivia.

^4^ Unidad de Limnología, Instituto de Ecología, Universidad Mayor de San Andrés, La Paz, Bolivia.

^5^ MARBEC,Univ Montpellier, CNRS, Ifremer, IRD, Montpellier, France.

^6^ Instituto de Investigaciones de la Amazonía Peruana (IIAP), Laboratorio de Biología y Genética Molecular (LBGM), Iquitos, Perú.

^7^ Departamento de Ictiología, Museo de Historia Natural, Universidad Nacional Mayor San Marcos, Avenida Arenales 1256, Jesús María 15072, 14, Lima, Peru

† Corresponding author: [cedric.mariac@ird.fr](mailto:cedric.mariac@ird.fr)

‡ These authors contributed equally to this work.

# Running head

Fish diversity and distribution in the Tuichi basin

# ABSTRACT

To date, more than 2400 valid fish species have been recorded in the Amazon basin. However, the census of this biodiversity is not evenly distributed geographically and some regions remain poorly documented. This is the case in the Beni basin and in particular in one of its main sub-basins, the Tuichi, which flows through the Madidi National Park in the Bolivian Amazonia. The knowledge of its ichthyological diversity is, however, essential for the management and protection of aquatic ecosystems, which are threatened by the development of infrastructures (dams, factories and cities), mining and deforestation. Environmental DNA (eDNA) is an efficient, reliable, rapid and non-destructive method for investigating fish biodiversity that has been relatively little used so far in the Amazon basin. We sampled eDNA from water in 34 sites in lakes and rivers in the Beni basin including 22 sites in the Tuichi sub-basin, during the dry season. We then implemented a metabarcoding approach to assess the species richness of the ichthyofauna in the Tuichi River basin and to better understand the biogeographical distribution patterns of fishes in relation to different ecological descriptors. We detected 252 fish taxa (207 at species level) among which 57 are newly identified for the Beni watershed. Species compositions are signiﬁcantly different between lakes and rivers but also between rivers according to their hydrographic rank and altitude. Furthermore, we show that diversity patterns in this basin are related to the different hydro-ecoregions through which the Tuichi flows and also to geological, pedological and geomorphological environmental variables. The eDNA approach makes it possible to identify and complete the inventory of the ichthyofauna in this still poorly documented Amazon basin . The dispersion of the eDNA, which we estimated to be a few kilometres, reveals marked and structured patterns of species composition. However, taxonomic identification remains constrained by the lack of reference barcodes in public databases and does not allow the assignment of all OTUs. Our results are discussed in the light of these distribution patterns and of the environmental variables that shape them and that can be taken into account in conservation and management strategies within the Madidi reserve and the Andean foothills rivers. These results on a relatively pristine Andean basin could serve as a baseline for future studies, including on other Andean tributaries facing diverses anthropogenic gradients.

KEYWORDS: Fish, metabarcoding, environmental DNA, Amazon, biogeography

# INTRODUCTION

The Amazon basin hosts the largest freshwater fish diversity on earth with 2,406 native species [1,2] as well as a high genetic diversity [3], but is facing increasing threats from anthropogenic activities such as deforestation, habitat degradation, overexploitation, hydropower dam building, invasive species and pollution, and from climate change [4–6]. Yet, the high number of new fish species described every year suggest that, as impressive as it already is, the species list of the Amazon basin is far from complete [7]. The Madeira is one of the most species-rich basins of the Amazon. It includes the Beni watershed, already considered vulnerable to anthropic pressure [1] and where knowledge gaps remain, such as in the Tuichi River. The Tuichi basin is included in the Madidi National Park and Natural Area of Integrated Management (hereafter abbreviated Madidi NP), a recognised biodiversity hotspot [8–10] and also likely hosts one of the few identified spawning area for many fish species [11]. It therefore appears urgent to implement conservation and management measures to protect this biodiversity and to fill known gaps in terms of collection and knowledge of fish species [1].

Recently, environmental DNA (eDNA) sampling has been developed as an efficient and reliable method for understanding the biodiversity of all taxonomic groups in an ecosystem [12–15]. Environmental DNA sampling is a rapid and non-destructive method. It can target small taxonomic groups and even detect rare or cryptic species found at low abundances. For example, in the Brazilian Atlantic forest in a single eDNA sampling the nine known species of Anuran, from the most abundant to the rarest, were all detected, demonstrating the sensitivity of the approach [16]. In herpetofauna, eDNA sampling detected different species of turtles and amphibians in lakes and rivers in North America including the rarest species [17]. In the streams of central Idaho rivers, the giant salamander (*Dicamptodon aterrimus*) and the Rocky Mountain tailed frog (*Ascaphus montanus)* two rare species of amphibian were detected thanks to eDNA sampling [18]. The eradication of an invasive species often requires early detection, and eDNA has proved more effective than traditional methods for detecting introduced buffalo toads in France [19], and the python, *Python bivittatus*, in Florida [20]. Similarly, species that were believed to be extinct or residual in a marine ecosystem, including large shark species, were detected using the eDNA approach, thereby facilitating the development of conservation strategies [21]. Aquatic mammals can also be detected, such as manatees, of which three endangered species of the genus *Trichechus* have been successfully detected in African, Amazonian and Indian rivers, helping to refine known distributions and establish conservation management strategies [22]. eDNA complements acoustic conservation monitoring for marine cetaceans in vast oceans [23]. So far, however, the eDNA method has been more rarely used to assess general biodiversity, especially in extremely biodiverse areas [24]. In the Neotropics, eDNA sampling provided information at a large scale on the biodiversity of the ichthyofauna, identifying 148 taxa including 132 species, although this study was limited by the size of the reference database [25]. Nevertheless, with suitable database development, eDNA will revolutionize research on rare or very discrete species, but also our understanding of biodiversity, both crucial aspects for the sustainable management of aquatic ecosystems.

Here, we present the first comprehensive eDNA assessment to the specific ichthyodiversity of the hydrological basin of the Tuichi River, a sub-basin of the Beni watershed located in the Bolivian Andean foothills, within the Madidi NP, carried out with the aim of complementing more traditional ichthyological sampling efforts for the park [8–10] and investigating the spatial distribution of its ichthyofauna. The information provided will be used for the management of the Madidi NP and serve as a reference for the management of other hydrographic basins in the tropical Andean foothills.

# METHODS

### SAMPLING

#### Study area and Collecting sites

The sampling area is located mainly in the Tuichi sub-basin in the Beni watershed, sub-basin of the Madeira river, the largest tributary of the Amazon (Figure 1). It is part of the Madidi NP, one of the largest protected areas in Bolivia covering 1,895,750 ha [26]. The Tuichi sub-basin constitutes a hydrological network of more than 7,100 km (including all tributaries) that extends into the Andes at an altitude of more than 6,000 m a.s.l. and flows 270 km to its confluence with the Beni River, 25 km upstream of the town of Rurrenabaque. Environmental DNA samples were collected from lakes and rivers between September and October in 2017, 2018 and 2019 (see Table S1 for details). This period of the dry season with low water levels allows for easier navigation. But more important, during this period the connectivity between lakes and rivers is interrupted, which ensures that eDNA collected in the lakes provide information on their own ichthyofauna without eDNA input from the main stream. In the Tuichi basin 22 sites were collected: 12 on the Tuichi River, eight on its main tributaries and two on tectonic lakes (Chalalán and Santa Rosa). Additionally, in order to assess a possible peculiarity of fish compositions in the Tuichi basin, we also sampled two sites on the Beni River, one upstream and one downstream of the confluence with the Tuichi, three tributaries of the Beni (Hondo, Quendéqué and Quiquibé rivers) upstream of the confluence with the Tuichi, one oxbow lake (Ruta) on the Hondo River, as well as 6 other lakes located downstream in the Beni basin, outside of the Madidi NP. A total of 34 sites were collected, 25 in rivers and 9 in oxbow lakes (Figure 1A-B) covering an altitudinal range from 200 to 4,505 m a.s.l. Overall, the time required to complete eDNA collections was approximately four weeks. Following an ordering system of drainage patterns [27], the Beni River, the main river in the watershed, receives the rank 1; the Tuichi, Quiquibé, Quendéqué and Hondo rivers were put in rank 2 and all the Tuichi’s tributaries in rank 3.

According to Wasson et al. (2002) geomorphological and climatic structures delineate nine geoclimatic domains sub-structured into 40 hydro-ecoregions (HER2) in the Bolivian Amazonian basin. HER are congruent with geology, relief, climate (temperature and precipitation), vegetation, soils and hydrography. Our sampling sites are distributed across 7 HERs corresponding to Glacial mountain (Cg), High peri-Amazonian Yungas (Ya-a), Amazonian gallery forest (Ab-a), Beni Gallery Forest (Ab-b), Dry valleys of the Yungas (Ay), High humid sub-Andean (Sa-a) and Low humid sub-Andean (Sa-b).

#### Ethic statement

The Wildlife Conservation Society (WCS) is authorized by the Bolivian government to study and sample the Bolivian Amazonian biodiversity. The Ministerio de Medio Ambiente y Agua (MMAyA) of Bolivia authorized collection, export and analysis of the filter cartridges to the French National Institute of Research for Sustainable Development (IRD) laboratories in France (FCPV-IE-0196/2017; FCPV-IE-0030/2018; FCPV-IE-0064/2019).

#### eDNA field sampling

Due to variations in water turbidity in these areas, particularly in streams, and in order to ensure sufficient filtered volume before clogging, we used sterile Envirocheck HV filter cartridge with a porosity of 1 µm (Pall Corporation, Ann Arbor, MI, USA). One cartridge per river sampling site was used, and two to five cartridges for lakes. For river sites where the width of the river was greater than 20 m, sampling was carried out by boat at three points, one in the middle and the other two a few metres from each bank. Otherwise a single point was sampled in the high-flow part of the river. The sampling points on the tributaries of the Beni and Tuichi rivers were carried out at least 200 m from their confluence. In lakes, sampling was carried out by boat and designed to integrate as much as possible spatial heterogeneity of eDNA distribution, as previously suggested in lentic ecosystems [29,30]: collecting points were distributed over the entire area of the lake (see Table S1). The total number of sampling points per lake ranged from seven for the smallest lake, Ruta, to 90 for lake Tumichuca (Table S1).

The sampling kit is composed of a drill driver (AEG GmbH -BSB12cli202c), a peristaltic pump (Watson-Marlow 313-DW) in which the marpene suction hose was connected upstream to 5-6 m of a polyethylene suction hose and downstream to a polypropylene hose connection ensuring connection to the filter cartridge. Lead sinkers and a suction strainer (2 mm mesh) were added to the end of the suction pipe to ensure ballasting and prevent the entry of large particles into the cartridge.

All materials were handled with sterile gloves and between each site, tubings, hose fittings and suction strainer were decontaminated in a 9% sodium hypochlorite solution for 10 min, then rinsed three times with commercial water. The driver unit was decontaminated using DNA away solution (Thermo Scientific™). In order to control possible contamination of equipment and cross contamination between sites, seven negative control cartridges interspersed throughout the sampling campaign were made by filtering 20 l of commercial water in the field.

Each eDNA sample was taken by filtration of water pumped directly into the medium, applying an initial flow of approximately 1 l/min. Filtration was carried out until the filter was clogged and during the whole sampling phase the cartridges were protected from light by aluminium foil. The final volume filtered for each cartridge varied between 6.5 and 75 l, depending on the clogging speed as a function of the turbidity level at each site. Total volume per site filtered ranged from 11 to 180 l.

At sampling completion, cartridges were ﬁlled with 150 ml of conservation buffer (Tris-HCl 0.1 M, EDTA 0.1 M, NaCl 0.01 M and N-lauroyl sarcosine 1%, pH 7.5–8) and stored at 4°C until DNA extraction between 1-2 months after sampling. A total of 58 cartridges were processed, including seven empty field controls and one alien control consisting of four species (*Alburnus alburnus, Carassius gibelio, Cyprinus carpio, Gambusia holbrooki*) never recorded in our study area.

### LABORATORY ANALYSIS

#### DNA extraction

The DNA extractions and the PCR amplification steps were carried out in dedicated and physically separated rooms. To minimize the risk of cross contamination, all PCR steps were prepared under a workstation (GuardOne® StarLab) allowing UV decontamination.

Three independent extractions were performed for each cartridge as follows: 5.4 ml of a 2.5 M NaCl solution were added to each cartridge to obtain a concentration of 0.1 M and 3 g of SDS to a final concentration of 2%. After a 2 h 30 lysis at 56 °C with stirring, the cartridge was emptied into 10 tubes of 50 ml. An additional volume of 7.5 ml of 3M K acetate is added to precipitate the proteins. After centrifugation for 30 min at 4°C at 6,200 g the supernatant was collected by pipetting to which 10 ml of isoamyl chloroform (24:1) was added, mixed by reverse (50 times), then centrifuged for 15 min at 15 °C and 6,000 g. The aqueous phase is recovered by pipetting and then 1.7 ml of Na acetate 3M pH 5.5 and 11 ml of cold isopropanol were added and mixed by inversion to precipitate DNA, then stored at -20 °C for 48h, centrifuged 30 min at 4 °C and 6,200 g. After elimination of the supernatant, the DNA pellets are washed with 1.5 ml of 70% ethanol, centrifuged for 15 mins and allowed to dry after elimination of the supernatant. The pellets are finally resuspended in 200 μl of TE. A 25 µl fraction is purified with XP ampure beads (1.8X) before amplification by PCR.

#### Primers design, PCR amplification of barcodes and library preparation

We designed two COI metabarcoding primers targeting Amazonian fishes: one of 185 bp (MK1) and another of 285 bp (MK2) which share the same forward primer and are positioned 5’ from the standard COI-5P barcode. The R Primerminer package [31] was used to extract the COI sequences available of the 10 main freshwater fish Orders present in the Amazon basin. A manual design of the primers was carried out on the 10 consensus alignments carried out for each Order. These two barcodes were amplified separately with the following primers: F1-TCHACHAAYCAYAAAGAYATYGGYACYCT with R1-ACYATRAARAARATYATYACRAADGC and F1 with R2-CARAARCTYATRTTRTTYATTCGNGG. Given the fact that eDNA samples are known to be generally quite fragmented (Harrison et al., 2019 for a review and Deiner et al., 2017b for contrasting results), we chose to use these two barcodes and compare their performance. The second barcode which completely overlaps the first is supposed to be more resolutive because it is longer but less sensitive given its length. The libraries were prepared according to a 2-step PCR [34]. Each library was indexed using unique combinations of double indexing to more effectively exclude chimeric amplicons and index hopping during downstream analysis (unexpected combinations were assigned as indeterminate). For each filter cartridge three libraries from three independent DNA extractions were amplified for both barcodes.

Two mock samples for which we know the exact taxonomic composition were included to assess the sensitivity and specificity of the barcodes used in this study. As controls, they also make it possible to validate the bioinformatic treatments implemented, because we expected that the estimation of their specific composition would be close to the known composition.

An additional control called "alien" was included to verify cross-contamination during the preparation and sequencing of libraries. This control was collected in a basin at IRD Montpellier (France), which contains only species not recorded in the Beni basin, from Cypriniformes and Cyprinodontiformes Order, then none should be detected in our samples if cross-contamination and hopping index are controlled. In addition to the 22 negative field controls (3 DNA per negative cartridge +1), 14 extraction negative controls and 6 PCR negative controls were also amplified and sequenced in the same run with the samples. Overall including negatives controls, positives controls and samples, 200 libraries for each barcode were pooled, then puriﬁed and sequenced (paired end, 150 bp) using Novaseq illumina platform at the Novogene Co., Ltd. facilities.

### DATA ANALYSIS

#### Data processing and taxonomic assignment

Amplicon sequences were analyzed using the FROGS pipeline [35] with standard operating procedures. Amplicons (MK1 and MK2) were split according to their primer sequences and size (120-280 nucleotides and 200-300) and clustered into operational taxonomic units (OTUs) using Swarm (aggregation distance: d=1). After chimera removal, OTUs were kept if present in at least one library and with a minimum abundance of three sequences.

OTUs alignment against a custom COI fish database was performed by blastn (version 2.8.1). This local database was built with fish COI sequences mined from GenBank and Bold and also implemented with published sequences [36], but not yet available under GenBank (Table S2). This final database has 160,944 entries representing 3,480 genera and 15,429 fish species distributed worldwide. For Peru and Bolivia among the 1,605 species currently known [37,38] only 684 species are represented in the COI database. Although this demonstrates the incompleteness of the COI database for Amazonian fishes, this barcode is currently the one with the best coverage in the number of species.

Blastn results were parsed using MEGAN software [39] in order to perform affiliation with a minimum score value of 150, a minimum percent identity of 97% and the naive LCA algorithm at minimum percent coverage of 50. The three types of negative controls (field, extraction and PCR) were used to identify and remove contaminating reads in sample libraries with the R package Microdecon [40]. The Maximum Sensitivity plus Specificity (MaxSSS) threshold value [41] was computed using the two positive controls (species composition known) and the R package ROCR 1.0–7 [42]. This minimum frequency at which a taxon is retained was applied in order to maximize the sum of True Positive Rate and True Negative Rate. To assess whether the sequencing effort was sufficient, the rarefaction curves for each library were performed with the R Vegan 2.5.6 package [43].

#### Taxonomic diversity and geographic distribution

Taxa richness (alpha diversity) and community composition (beta diversity) were computed with R packages phyloseq 1.28 [44] and heatmap3 1.1.6 [45].

To detect possible biogeographic entities, a hierarchical cluster analysis (HCA) was performed using the hclust function of the vegan 2.5.6 R package [43] to build a dendrogram clustering the sites according to their level of faunistic identity using Jaccard's binary distance and a Ward2 aggregation method. A bootstrap test with a number of 1000 replications was performed to evaluate the robustness of each node of the dendrogram using the package pvclust 2.2.0 [46]. A precise identification of a biogeographic structure with eDNA samples collected in the aquatic environment is only possible if its diffusion is spatially limited. We have therefore estimated, among our samples, the minimum distance at which species present at one site are not detected at the downstream site.

In order to assess the relevance of our sampling effort we computed the second order jacknife (Jack2) estimator [47] using the package Vegan with the functions specnumber and specpool and calculated the percentage of coverage as : observed number of species / Jack2 estimator) calculated for the entire study area and for each biogeographic entities defined by hierarchical cluster analysis. It should be noted that the Jack2 estimator (derived from the number of singletons and doubletons observed) only partially represents the actual species richness of the study area due to the incompleteness of the current COI database which contains only 42.6% of the known species in this Amazon watershed. Moreover, the species richness revealed in this study is also partial because our sampling was only carried out during the flooding period and therefore lacks species whose presence would be exclusively seasonal and outside this period.

#### Environmental factors correlated to the biogeographic structure

The link between environmental factors and the biogeographic structure of fish species was measured for four categories of variables noted at each site: 1) the geomorphological environment of the habitat, including the lake or river ecotype, the size of the lakes and their age, as well as the area of their hydrographic basin and the length of the hydrographic network that drains into them, 2) the geographical location, including latitude (LAT), longitude (LONG) and altitude (ALT), 3) the substrate, corresponding to the nature of the sub-soil and soil at the sampling point, according to five geological types (GEOL) and six pedological types (PEDO) and, 4) hydro-ecoregions (HER), which integrate some of the above variables and define 7 different categories for the sites sampled in this study (Figure 1).

Lake sizes, hydrological networks length and basin area values were extracted for each site using Hydro Atlas [48] under QGIS version 3.4.12 [49] from GIS layers obtained at the World Data Bank (<https://datacatalog.worldbank.org/dataset/bolivia-soil-classification>; <https://catalog.data.gov/dataset/south-america-geologic-map-geo6ag>) and from Schneider et al., 2017 (Table S3).

Non-metric multidimensional scaling (NMDS) using Jaccard's binary distance was performed and correlations between environmental variables and NMDS ordination were measured.

Considering that some variables are not independent, such as longitude and altitude, the objective of this analysis is therefore to identify environmental variables which are not necessarily explanatory but that can be correlated with the structuring of the ichthyofauna. Correlations between environmental variables and NMDS ordination were computed with the function “envit” in the R package Vegan. A similarity analysis (ANOSIM, package Vegan 2.5.6) was performed to test whether the biogeographic entities identified by the HAC were significantly different in the NMDS projection. Data analyses were completed in R version 3.6.2 [51].

All command lines and scripts used for bioinformatic treatment, statistical analyzes and carrying out graphs are available in S1 Text.

# RESULTS

### SEQUENCING, ASSIGNATION and CONTROLS RESULTS

#### NGS sequencing and taxonomic assignment results

Overall for the 200 libraries, which included 154 samples, two controls, one alien sample, 42 negatives (6 PCR-blank, 22 negatives field controls, 14 negatives DNA extraction), sequencing produced 551.8 million raw reads (82 Gb). The mean number of reads per site was 14.7 million (SE = 12.6), see Table S4 for details.

Five libraries with abundance below 50 sequences assigned to fish have been excluded from the analyses: they included all libraries (3) from one site on the Tuichi (not reported), and one library each for the Chalalán lake and the Tuichi site 14. Rarefaction curves done at the library and site level (Figure S1, A-B) show that sequencing effort was enough for the remaining libraries.

After cleaning reads, 157.6 million could be joined and were clustered in 210,035 OTUs. Of these, 5,246 OTUs made up from 12.59 million sequences were assigned to a fish COI reference (median value per site = 170,742), 93.6% at the species level. OTUs (n=8,502) with a match with a COI reference in the database, but with scores or percentages of identity values below the thresholds were discarded. Finally, 196,287 OTUs had no hit.

Four taxa were discarded because they matched to marine and African species (*Epinephelus marginatus* JX124778.1, *Marcusenius monteiri* AMNHI782-12, *Sebastiscus marmoratus* HM180872 and *Zoarces gillii* HM180941). These reference sequences mined in GenBank were parsed under Boldsystem taxonomic identification [52], showing that taxa names associated with their relative sequence are incorrect.

One hundred-two taxa were shared by the two primer sets (MK1 and MK2) and 90 taxa were unique to a single primer set (73 for MK1 and 17 for MK2). Although barcode MK1 is smaller in size than MK2 (185 and 285 bp, respectively) their taxonomic resolutions were similar, with more than 90.6% and 92.7% of the OTUs assigned at the species level. The percentage of identity between the OTU and the reference sequences of the database to which they are assigned was on average 98.71% (SE = 1.12). Taxa abundance per library are reported in Table S5.

#### Controls by negative, positive and alien samples

Of the 42 negative control libraries only 213 reads were assigned to fish taxa indicating a low level of contamination. Of 40 species in the mock control samples 39 were identified. These mock samples were used to set a minimum frequency at which a present taxon was declared. This threshold was calculated to maximize the rate of true positive and to minimize the rate of false positive and set a minimum value of 0.01% (MaxSSS) from which a taxon identified in a library is kept. In the "alien" sample, the four expected taxa (which are absent in the Amazon) were identified. A maximum of three sequences belonging to a species from this alien control were found in an Amazonian sample, which shows a low level of leakage or contamination occurred during library preparation or the sequencing process. On the basis of this estimated leak rate, only the taxa represented by at least four sequences per site were kept.

### BIODIVERSITY

#### Taxonomic diversity in the study area

On all the sites, 252 taxa were identified covering 79.8% of the estimated number of identifiable taxa (Jack2=315) given the limitations of our database (Figure 2). Among these taxa, 22 are identified to the genus level, 207 at the species level and 23 at the level of undescribed species (noted sp.) and all are described in South America (FishBase, [53]. Among the 207 species identified 150 have already been identified in Bolivia [2,38]. The 57 species never identified to date in Bolivia were verified on the basis of systematics and biogeography. Of these, 16 were named *affinis* (although they had a hit of more than 97% identity) because they were never identified in the Amazon basin. In addition, for each identified taxon we reported their percentages of identity with the COI reference to which they are assigned, as well as the total read number (Table S6). This table also reports the records of these taxa in the Amazon and the Beni region.

These 252 taxa are distributed among 149 different genera in 11 orders: 45.2% of the taxa are Siluriformes, 34.5% Characiformes, 9.1% Gymnotiformes, 6.0% Cichliformes. The orders Clupeiformes, Myliobatiformes, Perciformes, Synbranchiformes, Salmoniformes, Osteoglossiformes, Cyprinodontiformes together account for the remaining 5.2% of the identified taxa.

The number of taxa per site averaged 51.03 (SD = 23.1) and ranged between 2 to 105. Overall, 53.5% of taxa were identified at four sites or less (Figure S2). A single taxon, *Prochilodus nigricans*, was observed at all sites except Pelechuco which is located at 4,508 m a.s.l. At this site only *Orestias aff. ascotanensis* (agassizii group) and *Oncorhynchus mykiss* were identified.

A significant correlation was observed between taxa richness observed and altitude (r = 0.85, *p*-value = 0.011), with a regular pattern of increasing richness towards downstream sites (Figure 3). In rivers, taxa richness ranged from 2 at Pelechuco (4,508 m a.s.l.) to 95 in the Beni river (210 m a.s.l.). In lakes, the two most upstream have the lowest richness values (21 and 22 taxa identified in Chalalán and Santa Rosa, respectively) and the highest was observed in the Colorada lake with 105 taxa identified (see Figure S3 and Table S3 for details).

#### Geographic distribution patterns of species detected in the Tuichi basin and the Beni lakes

The hierarchical clustering of the sampled sites shows four clusters, representing four biogeographic entities that are supported by bootstrap values greater than 81% (Figure 4). The nine lakes constitute a first exclusive biogeographic entity, separating the three most-upstream lakes (Ruta, Chalalán and Santa Rosa) from the downstream lakes. The second (Alto Tuichi) brings together the four upstream sites collected on the Tuichi river, the third includes all the sites collected on the rank 3 rivers, and finally the fourth entity groups the tributaries of rank 1 and 2. The calculated coverage rates (number of observed taxa / estimated number of taxa using Jack2 estimator) for each of the four biogeographic entities (lakes, Alto Tuichi, rank 1 and 2 rivers and rank 3 rivers) range from 67.9% to 79.7% and are not significantly different from each other (*p*-value = 0.09) showing that our sampling effort is similar among these biogeographic entities and therefore allows for comparison.

In rivers, 172 taxas were identified, of which 52.3% and 32.6% belonged to Siluriformes and Characiformes, respectively. In lakes, 153 taxa were identified, including 32% of Siluriformes and 39.9% of Characiformes. As illustrated in Figure 4, Siluriformes (brown lines) had a significantly stronger occurrence (*p*-value < 0.001) in rivers than lakes whereas Characiformes (red lines) had a stronger occurrence in lakes, although this trend was not significant (*p*-value > 0.1). Taxa belonging to the Clupeiformes and Osteoglossiformes orders were detected only in lakes, whereas Cyprinodontiformes and Salmoniformes were only detected in rivers.

In the Madidi NP alone, of the 186 taxa identified, 17 taxa are common to lakes and rivers, 14 taxa identified only in lakes and 140 taxa identified only in rivers (Figure 5). Considering the four biogeographic entities established by the hierarchical analysis, their difference in terms of species composition remains strongly marked. Only 7 taxa are common to these groups. Six taxa were identified only in the Alto Tuichi group, six in the rank 3 rivers, and 54 in the rank 1 and 2 rivers group (see Table S7 for taxa lists in each area of the Venn diagram).

Between biogeographic entities the number of taxa shows a clear increase between the Alto Tuichi (46 taxa) above 700 m a.s.l and those at lower altitude (Figure 3). Below 700 m a.s.l., species diversity follows the increase in rank and therefore in size of the sub-basins, going from 98 taxa (rank 3 tributaries) to 155 taxa (rank 1 or 2 tributaries).

#### Estimation of eDNA detection distances in streams and lakes

We assessed the minimum distance from which detectability is no longer possible in the environmental context (dry season) of our sampling. For this, we used the situation where taxa were present at a site, but not detected at the site sampled immediately downstream. Of the species identified at the Arana river site (point c on Figure 1-B), 23 species are not identified at the Tuichi8 site (point 9 on Figure 1-B) located only 3.7 km downstream. These 23 species have an average detection rate at the Arana site of 71% and for 9 of these species it is 100% (i.e. identified in all Arana libraries). Given that sampling in lakes was carried out in the dry season without water entering from the main river, DNA detected was emitted only by species present in the lake and in its tributaries. Therefore, in lakes, as well as in rivers, the eDNA approach provides a localized view of species distribution by site which is estimated at a few km in our case study, as evidenced by the strong structure of the species distribution observed among 4 clusters totally independent of the geographical distance, hence of the stream flow (Figure 3-B).

#### Environmental factors correlated to the biogeographic structure

The NMDS based on Jaccard distances between sites in fish composition had a stress value of 0.09 showing that the representation was highly reliable. Fish populations are differentiated between ecotypes, rivers and lakes (r2 = 0.52, *p*-value < 0.0001) (Figure 6-A and Figures S4-S5 for details) and the analysis of variance using distance matrix confirmed a significant separation between the four biogeographic entities defined by the hierarchical clustering (*p*-value *=* 0.0009).

Hydro-ecoregions, longitude, altitude and pedology variables are correlated with biogeographic entities NMDS ordinations of the ichthyofauna both between rivers (Figure 6-B, Table 1) and between lakes (Figure 6-C, Table 1) with *p*-value < 0.01. Geology variation is correlated between river ichthyofauna compositions (Figure 6-B, Table 1). The area of the hydrological basins and the length of the network flooding the lakes are also correlated (*p*-value < 0.05) with the biogeographic structure of the ichthyofauna between lakes, but the age and area of the lakes has no observed influence on their faunistic composition (Figure 6-C). The Quendeque river of rank 2, which is positioned by the hierarchical classification with all other rivers of the same rank in the yellow group (Figure 4), has a position approaching the rivers of rank 3 (green group) according to the NMDS analysis (Figure 6-B). This faunistic peculiarity of the Quendeque River could be explained by the small size of its hydrographic basin and network compared to other rivers of rank 2, as well as by its ecological characteristics (only site located in high humid sub-andean HER).

### **Table 1:**

Correlations and significance levels between environmental variables and NMDS ordinations. These statistics are reported for the 3 NMDS ordinations carried out on i) rivers only, ii) lakes only and iii) all sites.

# DISCUSSION

#### Relevance of the eDNA approach to inventory

Since the beginning of the 19th century until recently, 444 species of fish have been identified in the Beni basin [1]. Our eDNA approach, although carried out in a small portion of the same basin (10% of the area of the Beni basin) and during a single hydrological period, has resulted in a list of 207 species. Among these species, 57 are newly identified for the Beni watershed as compared to the current most exhaustive census compiled by [1].

In accordance with recent recommendations [54], which identified 87% of expected fish fauna in small streams with a single 34 l sample, we filtered an average of 38 l of water per site which allowed us to collect sufficient eDNA and achieve an mean coverage of 79.8% of the estimated number of species under the limits of the available databases. Although increasing the sequencing effort could significantly increase the observed species diversity as shown by [55], sequences rarefaction tests performed on our samples show that this effort was sufficient (SFigure1-A).

It is worth noting that the results presented here were achieved in a 4-week sampling timeframe, for an average analysis and sequencing cost of approximately $450 per site, further emphasizing the effectiveness of the eDNA approach to inventory species. This is an extremely low cost compared to that of sampling specimens over decades, and which has enabled us to increase the species inventory by 11.4% in a watershed that has been inventoried for over a century. Some of these newly detected species could be cryptic or very scarce, or new species for science but not yet described because they are morphologically similar to already described species: for example, the species of the genus *Prochilodus* noted affinis in this paper, and probably already identified by metabarcoding of bulks of larvae further downstream from our study area [11].

#### A limited detectability of eDNA in the Amazon region

Our study showed that some species that were detected at a sampling site were no longer detected a few kilometres downstream (around 3 km) at the next sampling site. However, it should be noted that the sites where these species were identified with their DNA do not necessarily correspond to their locations, so this distance may be underestimated. Although it is difficult to compare diffusion distances between different studies, which depends on detectability and therefore on the experimental set-up (volume of water filtered, fraction of DNA analysed, etc.), Civade et al. ( 2016) estimated diffusion distances in French Guiana at between 2 and 3 km, similar to those observed here. However, in the Rhône in France, eDNA was found between a few km to 130 km from its emitting source, but with a detection rate below 0.4% for the latter [57].

A hypothetical explanation could be that the detectability of fish species in the Amazon basin could be lower than in other regions. As the specific diversity in the Amazon is much greater than that observed at temperate latitudes, if the biomasses are similar, the eDNA concentration for each Amazonian species would therefore be lower, reducing their detectability.

In our case, *Orestias ascotanensis*, present at the extreme upstream Pelechuco site (a creek of less than 1 m width), was not detected at the Virjen site located 61.7 km downstream (a river over 100 m wide) even though eDNA could drift down this distance in less than 24 hours given that the speed of current at Virjen was around 0.7 m/s.

It is therefore difficult to attribute this lack of detection to a total degradation of DNA, given that the half-life of DNA molecules in the aquatic environment is generally considered to be several days, even in tropical conditions [58,59].

An additional explanation could be the increase in flow rate which, by increasing eDNA dilution, would decrease detectability as highlighted by [60] who showed the influence of both temperature and stream flow level on *Corbicula fluminea* (clam) detectability. In our case study, both the high water temperature (mean over sites =25°C, SE=4.9) and the large and rapid increase in river discharge, are likely to limit the detectability of eDNA a few kilometres downstream of its emission. We can also assume that the length of our barcodes (185-285 bp), which is greater than those sometimes used, such as 12S (65 bp), would allow us to target less degraded DNA, which has been recently released into the environment and has not diffused very much. The fact that 73 taxa were only detected by the MK marker (185 bp) compared to the 17 detected only by MK2 (285 bp), tend to lend support to this hypothesis. The use of long barcodes might thus provide a more local image of the diversity detected.

The only exception to this trend is the rainbow trout, *Onchorhynchus mykiss*, a freshwater Salmoniformes, that was unexpectedly detected in several sites of the Tuichi and Beni basins (Table S7), usually with high identity (>98%). Given what was just explained about the limited diffusion of eDNA, this species, which lives in cold waters at high altitude should not be encountered in the lower portion of the Tuichi River, nor in the Beni River. A likely explanation, however, is the presence of many aquaculture facilities in the Andean portion of the Tuichi and in several of its tributaries. The presence of trout in very large, unnatural, concentration, as is the case in intensive aquaculture, could release large amounts of DNA in the waters that could disperse downstream in spite of the above mentioned factors limiting eDNA diffusion.

#### Biogeographic structure

The fish species composition revealed among the sites of this study through environmental DNA provides a sufficiently localised signature for a biogeographic pattern to emerge. The sampling sites were close to the confluences of the Quiquibey, Quendeque and Hondo rank 2 rivers and the rank 3 tributaries of the Tuichi, allowing the identification of the fauna located at only few km upstream of the sampling points. In oxbow lakes and tectonic lakes, the absence of inflows from streams allows the identification of the local ichthyofauna present at the sampled site. Four biogeographic entities are highlighted according to the specific composition of their ichthyofauna in relation to the structure of the hydrographic network (rank 1-2, rank 3, Alto-Tuichi) with an even stronger distinction of the ichthyofauna between rivers or lakes biotopes constituting the fourth entity. The ability of eDNA to more accurately report on the structuring of species than more traditional inventories (net, electric fishing, rotenone) is recognized [25,61]. The entire ecosystem is better apprehended with a sharp reduction in the biases inherent to sampling a complexity of habitats in which rare and cryptic species would be much more difficult to access and register by traditional means. Similar richness coverage between the 4 biogeographic entities (from 70 to 80%) reflecting similar sampling efforts allows comparing their ichthyodiversity with limited bias.

The distribution patterns of the ichthyofauna according to the ranks of the tributaries and therefore according to their sizes, have been demonstrated many times in all latitudes (review in Hugueny, Oberdorff, & Tedescco, 2010). In the Beni basin, the patterns of differentiation between lake and river ichthyofauna are particularly contrasting with all the lakes from upstream to downstream of the Beni clustered in the same biogeographic entity. Furthermore, two sub-units can be distinguished among lakes: the lakes of the Rio Beni plain and those located in the upstream portion of the basin in the Madidi reserve (lakes Ruta, Santa Rosa and Chalalán). For Santa Rosa and Chalalán lakes, which are tectonic and older, species diversity is lower than in the oxbow lakes, however, for the latter neither their age of appearance, nor their surface area is correlated with their species richness. We also note that lake Ruta, although young (2005) and small (4 ha), has the same number of species as the older (<1950) and much larger (180 times) lake Mentiroso located in the Madre de Dios plain (Figure 3). In contrast with the Ruta lake, Mentirosso is under strong human pressure (fishing, mining activities).

The distribution of fish populations’ biogeographic entities in the Tuichi basin is strongly correlated with hydro-ecoregions patterns [28] that include several local environmental variables, sometimes correlated with each other, highlighting the relationship between fish distribution and waters quality in the Amazon [63]. The physico-chemical parameters involved in water quality have not been directly measured in this study, but are nevertheless integrated in the four main categories of variables considered. The biogeography of the lake ichthyofauna is correlated to altitude, itself correlated to the longitude and the nature of soils. The composition of the ichthyofauna of the different rivers is correlated with the same environmental parameters as lakes, to which is added geology, itself in relation to altitude. Usually, the nature of the substrate on which the continental waters flow (river and lake) partly explains the distribution of the Amazonian ichthyofauna [64], since the variation of the soils and subsoils drives the variation of the physicochemical parameters of the water, the living environment of the fish, as well as the type of riparian vegetation, which is itself a food resource and nursery for numerous fish species [65].

In the present study the observed species richness drops considerably with altitude in lotic biotopes, with less than a dozen species detected only above 700 m elevation. The decline in species richness with increasing altitude is described as being related to decreased temperature, an increase in dissolved oxygen and pH, also accompanied by a turnover of species, as observed in the Colombian Andes [66]. However, this drop in diversity is also accompanied by an increase in the proportion of endemic species in the Andean foothills: around 40% of the 400 to 600 species inventoried, including species that are threatened or already extinct [67]. Moreover, this increase in endemicity with altitude would correspond to a diversification of the functional groups distributed in the basin headwaters, generating an increase in beta diversity [68].

#### Human threats and conservation

Many watersheds originating in the Andean valleys are under increasing anthropogenic pressure due to the construction of dams, gold mining [69] and pollution by wastewater from cities and industries (e.g. construction of sugar mills close to sites such as the Fuerte lake). In order to set up conservation strategies, the biodiversity of the aquatic fauna distributed in particular in the Andean foothills should be generalised and investigated rapidly, as we have done for the Tuichi basin, using eDNA approaches. Owing to its location directly downstream of the capital city, La Paz, and its anthropogenic pressure (mining, pollution, landscape degradation, exploitation;[11]), but also to the presence of the Madidi NP and knowledge gaps [1], the Beni basin should be considered a priority area for carrying out further eDNA surveys.

The results and methodology of the present study could serve as a reference point for future comparisons of river basins under different anthropogenic pressures with the lightly anthropized Andean basin of the Tuichi River. On a larger scale, anthropogenic effects in the Beni watershed, and in particular fishing pressures that remain poorly documented [70], should be estimated and related to the specific assemblages and richness observed. Another growing anthropogenic threat is the rapid expansion of hydroelectric dams in the Amazon basin [71–75]. Three dams are currently planned in our study area (Figure 1), between the protected areas of the Madidi NP: one at the Bala strait in the middle of our study area, and two slightly upstream of it at the Chepete and Beu straits [11]. Their construction would lead to > 50% losses of network connectivity in the Beni basin [71]. It would also result in profound alterations in the sedimentation patterns of the river (volume and flow of sediments), homogenizing downstream habitats in the river and in the lakes [76]. Additionally, these dams would also result in the formation of extensive upstream reservoirs and the Bala dam would likely permanently flood a large portion of our study area. Other foreseeable impacts of these dams are similar to that observed on riverine fish communities elsewhere in the world and range from local extinction of species that cannot adapt to reservoirs and dam-tailwater conditions, to basin-wide extirpations of migratory species [77,78]. The Amazon basin hosts a rich migratory fish fauna that accounts for the bulk of fisheries landing and dam construction is expected to severely impacts migratory species (review in Duponchelle et al., 2021). Several of these migratory species holding commercial and cultural importance were identified in our study area. A spawning area for several of these species, threatened by anthropogenic activities such as arid extraction or pollution, was recently identified in our study area [11]. The alteration of local environmental conditions by damming (sediment and flow patterns modifications, permanent flooding of large area) will likely further compromise the usefulness of the identified spawning area. It will profoundly modify the structure of fish communities and the fisheries that depend on them, as observed in other dammed rivers of the Amazon basin (review in Duponchelle et al., 2021).

#### Contributions and limitations of the eDNA approach in the biogeographic study of the region

The eDNA approach permitted a study of fish biodiversity in the Tuichi sub-basin and broader Beni basin within and immediately adjacent to the Madidi NP with a high species diversity thanks to a high taxonomic resolution, since 93.3% of the assigned OTUs (5,246) are at the species level. A majority of OTUs (8,205) although aligned with at least one sequence in the COI fish database could not be assigned taxonomically due to a too low percentage of identity with the reference (<97%). This is closely related to the fact that of 2,406 known Amazonian fish species [2], only a third (694) have a COI barcode available. The effectiveness of the method would therefore be considerably enhanced if the reference databases used for taxonomic attribution (Bold, GenBank) were improved over time, thereby improving our species identification capabilities.

Markers other than COI have been used to record fish biodiversity, such as a 12S marker in French Guiana [25]. But the smaller size of the latter (65 bp versus 185-285 bp for the COI barcodes used here) and an even more limited database (181 available 12S barcode in Cilleros et al., 2018) than that of the COI, makes this marker less efficient than the COI, with more difficulty in assigning OTUs below family or gender level. The combination of several markers [80] and the availability of more exhaustive reference databases would allow a more accurate appreciation of species diversity. The eDNA approach, already widely used for species inventories, also seems to be a promising tool for localised studies to assess the impacts of anthropogenic disturbance on biodiversity, such as dams, factories or mines, but also for carrying out a rapid census of biodiversity on more extensive regional scales. Ecological and conservation studies will require the complementarity of traditional sampling approaches to provide information on recruitment, age, size and precise abundance, as well as to identify the specimens needed to complete the DNA reference databases. Nevertheless, eDNA approaches to access large-scale species inventories in a rapid and non-destructive manner can complement these studies. Quantitative approaches based on eDNA sampling are being developed using qPCR or digital PCR techniques, in order to gain access to relative biomasses [81,82]. Although many technical or biological biases still need to be overcome, access to biomass would provide information on the demographic patterns of species required to put in place conservation strategies essential for sustainable ecosystem management. Fishes are among the freshwater biota most threatened by global changes [83,84]. The evolution of fish biodiversity in the Tuichi study area could be monitored over several years in relation to climate change and dynamic anthropogenic threats in the region, and serve as a reference for the study of other river basins in the Amazon.

# Acknowledgments

This study was financed by IRD and the Wildlife Conservation Society. The LMI EDIA and UMR DIADE financed the metabarcoding analyses. The authors acknowledge the IRD itrop HPC (South Green Platform) at IRD Montpellier for providing HPC resources that contributed to the research results reported within this paper (URL: <https://bioinfo.ird.fr/>- <http://www.southgreen.fr>).

# Conflict of interest

The authors declare they have no conflict of interest.

# Author Contributions

CM, FD and JFR conceived this study. CM, GM, CR, GT, RW and JFR participated in sampling design, collected and processed the samples. CM, CGD and JFR carried out laboratory analyses. CM, FD , JFR analysed the data and wrote the manuscript and GM, CGD, CR, GT, HO, JP, RW worked on the interpretation of the data and critically reviewed the manuscript.

# Data Availability Statement

Sequencing reads were deposited in the National Center for Biotechnology Information Sequence Read Archive (BioProject ID: PRJNA661268, BioSample accessions: SAMN16049263- SAMN16049300).

# References

1.	Jézéquel C, Tedesco PA, Bigorne R, Maldonado-Ocampo JA, Ortega H, Hidalgo M, et al. A database of freshwater fish species of the Amazon Basin. Sci Data. 2020 Dec;7(1):96.

2.	Jézéquel C, Tedesco PA, Darwall W, Dias MS, Frederico RG, Hidalgo M, et al. Freshwater fish diversity hotspots for conservation priorities in the Amazon Basin. Conservation Biology. 2020 Jan 28;cobi.13466.

3.	Manel S, Guerin P-E, Mouillot D, Blanchet S, Velez L, Albouy C, et al. Global determinants of freshwater and marine fish genetic diversity. Nat Commun. 2020 Dec;11(1):692.

4.	Castello L, McGrath DG, Hess LL, Coe MT, Lefebvre PA, Petry P, et al. The vulnerability of Amazon freshwater ecosystems: Vulnerability of Amazon freshwater ecosystems. Conservation Letters. 2013 Jul;6(4):217–29.

5.	Castello L, Macedo MN. Large-scale degradation of Amazonian freshwater ecosystems. Global change biology. 2016 Mar;22(3):990–1007.

6.	Hurd LE, Sousa RGC, Siqueira-Souza FK, Cooper GJ, Kahn JR, Freitas CEC. Amazon floodplain fish communities: Habitat connectivity and conservation in a rapidly deteriorating environment. Biological Conservation. 2016 Mar;195:118–27.

7.	Reis RE, Albert JS, Dario FD, Mincarone MM, Petry P, Rocha LA. Fish biodiversity and conservation in South America. Journal of Fish Biology. 2016;89(1):12–47.

8.	Wallace R, Ayala G, Negroes N, O’Brien T, Viscarra M, Reinaga A, et al. Identifying Wildlife Corridors Using Local Knowledge and Occupancy Methods along the San Buenaventura-Ixiamas Road, La Paz, Bolivia. Tropical Conservation Science. 2020 Jan 1;13:1940082920966470.

9.	Wallace R, Ramírez A, Salinas E, Painter L. Inspirando a la población urbana mediante información científica e imágenes de biodiversidad - Identidad Madidi. Ecología en Bolivia. 2017 May;52(1):1–6.

10.	Wallace R, Ramírez Yaksic A, Salinas E, Zentero Ruiz F, Ayala G, Hoverud NB, et al. Informe científico 2016 identidad Madidi: relevamientos de biodiversidad en el Parque Nacional y Área Natural de Manejo Integrado Madidi. La Paz, Bolivia; 2019. 200 p. (Identidad Madidi & SERNAP).

11.	Miranda-Chumacero G, Mariac C, Duponchelle F, Painter L, Wallace R, Cochonneau G, et al. Threatened fish spawning area revealed by specific metabarcoding identification of eggs and larvae in the Beni River, upper Amazon. Global Ecology and Conservation. 2020 Dec;24:e01309.

12.	Deiner K, Bik HM, Mächler E, Seymour M, Lacoursière-Roussel A, Altermatt F, et al. Environmental DNA metabarcoding: Transforming how we survey animal and plant communities. Mol Ecol. 2017 Nov 1;26(21):5872–95.

13.	Ficetola GF, Miaud C, Pompanon F, Taberlet P. Species detection using environmental DNA from water samples. Biology Letters. 2008 Aug 23;4(4):423–5.

14.	Yoccoz NG. The future of environmental DNA in ecology. Molecular Ecology. 2012;21(8):2031–8.

15.	Bohmann K, Evans A, Gilbert MTP, Carvalho GR, Creer S, Knapp M, et al. Environmental DNA for wildlife biology and biodiversity monitoring. Trends in Ecology & Evolution. 2014 Jun 1;29(6):358–67.

16.	Lopes CMA-O http orcid org, Sasso T, Valentini A, Dejean T, Martins M, Zamudio KRA-O http orcid org, et al. eDNA metabarcoding: a promising method for anuran surveys in highly diverse tropical forests. 2017;(1755-0998 (Electronic)).

17.	Lacoursière-Roussel A, Dubois Y, Normandeau E, Bernatchez L. Improving herpetological surveys in eastern North America using the environmental DNA method. Genome. 2016 Aug 30;59(11):991–1007.

18.	Goldberg CS, Pilliod DS, Arkle RS, Waits LP. Molecular Detection of Vertebrates in Stream Water: A Demonstration Using Rocky Mountain Tailed Frogs and Idaho Giant Salamanders. PLOS ONE. 2011;6(7):e22746.

19.	Dejean T, Valentini A, Miquel C, Taberlet P, Bellemain E, Miaud C. Improved detection of an alien invasive species through environmental DNA barcoding: the example of the American bullfrog Lithobates catesbeianus. Journal of Applied Ecology. 2012;49(4):953–9.

20.	Piaggio AJ, Engeman RM, Hopken MW, Humphrey JS, Keacher KL, Bruce WE, et al. Detecting an elusive invasive species: a diagnostic PCR to detect Burmese python in Florida waters and an assessment of persistence of environmental DNA. Mol Ecol Resour. 2014 Mar 1;14(2):374–80.

21.	Boussarie G, Bakker J, Wangensteen OS, Mariani S, Bonnin L, Juhel J-B, et al. Environmental DNA illuminates the dark diversity of sharks. Science Advances. 2018 May 1;4(5):eaap9661.

22.	Hunter M, Meigs-Friend G, Ferrante J, Takoukam Kamla A, Dorazio R, Keith-Diagne L, et al. Surveys of environmental DNA (eDNA): a new approach to estimate occurrence in Vulnerable manatee populations. Endangered Species Research. 2018 Mar 13;35:101–11.

23.	Foote AD, Thomsen PF, Sveegaard S, Wahlberg M, Kielgast J, Kyhn LA, et al. Investigating the potential use of environmental DNA (eDNA) for genetic monitoring of marine mammals. PLoS ONE. 2012;7(8):e41781.

24.	Jerde CL, Wilson EA, Dressler TL. Measuring global fish species richness with eDNA metabarcoding. Molecular Ecology Resources. 2019;19(1):19–22.

25.	Cilleros K, Valentini A, Allard L, Dejean T, Etienne R, Grenouillet G, et al. Unlocking biodiversity and conservation studies in high-diversity environments using environmental DNA (eDNA): A test with Guianese freshwater fishes. Molecular Ecology Resources [Internet]. 2018 [cited 2018 Jul 6];0(0). Available from: https://onlinelibrary.wiley.com/doi/abs/10.1111/1755-0998.12900

26.	Toomey A. The Making of a Conservation Landscape: Towards a Practice of Interdependence. Conservat Soc. 2020;18(1):25.

27.	Strahler AN. Quantitative analysis of watershed geomorphology. Trans AGU. 1957;38(6):913.

28.	Wasson J-G, Barrera S, Barrère B, Binet D, Collomb D, Gonzales I, et al. Hydro-ecoregions of the Bolivian Amazonian Basin: the geographical frame of running water ecosystems functioning. In: The Ecohydrology of South American Rivers and Wetlands. International Association of Hydrological Sciences (IAHS) Press. Wallingford: M. E. McClain; 2002. p. 69–91. (IAHS; vol. 6).

29.	Lawson Handley L, Read DS, Winfield IJ, Kimbell H, Johnson H, Li J, et al. Temporal and spatial variation in distribution of fish environmental DNA in England’s largest lake. Environmental DNA. 2019 May;1(1):26–39.

30.	Li J, Lawson Handley LJ, Harper LR, Brys R, Watson HV, Di Muri C, et al. Limited dispersion and quick degradation of environmental DNA in fish ponds inferred by metabarcoding. Environmental DNA. 2019 Sep;1(3):238–50.

31.	Elbrecht V, Leese F. PrimerMiner: an r package for development and in silico validation of DNA metabarcoding primers. Methods in Ecology and Evolution. 2017;8(5):622–6.

32.	Harrison JB, Sunday JM, Rogers SM. Predicting the fate of eDNA in the environment and implications for studying biodiversity. Proc R Soc B. 2019 Nov 20;286(1915):20191409.

33.	Deiner K, Renshaw MA, Li Y, Olds BP, Lodge DM, Pfrender ME. Long-range PCR allows sequencing of mitochondrial genomes from environmental DNA. Methods in Ecology and Evolution. 2017;8(12):1888–98.

34.	Galan M, Pons J-B, Tournayre O, Pierre É, Leuchtmann M, Pontier D, et al. Metabarcoding for the parallel identification of several hundred predators and their prey: Application to bat species diet analysis. Molecular Ecology Resources. 2018 May;18(3):474–89.

35.	Escudié F, Auer L, Bernard M, Mariadassou M, Cauquil L, Vidal K, et al. FROGS: Find, Rapidly, OTUs with Galaxy Solution. Berger B, editor. Bioinformatics. 2018 Apr 15;34(8):1287–94.

36.	Guimarães KLA, de Sousa MPA, Ribeiro FRV, Porto JIR, Rodrigues LRR. DNA barcoding of fish fauna from low order streams of Tapajós River basin. Doi H, editor. PLoS ONE. 2018 Dec 21;13(12):e0209430.

37.	Ortega H, Hidalgo M, Giannina T, Correa E, Cortijo AM, Meza V, et al. Lista anotada de los peces de aguas continentales del Perú:  Estado actual del conocimiento, distribución, usos y aspectos de conservación. Ministerio del Ambiente DG de DB-M de HN UNMSM, editor. 2012.

38.	Sarmiento J, Bigorne R, Carvajal-Vallejos FM, Maldonado M, Leciak E, Oberdorff T. Peces de Bolivia, Bolivian fishes [Internet]. Primera edición = First edition. La Paz : Plural Editores, 2014. ©2014; 2014. Available from: https://search.library.wisc.edu/catalog/9910217107002121

39.	Huson DH, Beier S, Flade I, Górska A, El-Hadidi M, Mitra S, et al. MEGAN Community Edition - Interactive Exploration and Analysis of Large-Scale Microbiome Sequencing Data. Poisot T, editor. PLOS Computational Biology. 2016 Jun 21;12(6):e1004957.

40.	McKnight DT, Huerlimann R, Bower DS, Schwarzkopf L, Alford RA, Zenger KR. microDecon: A highly accurate read-subtraction tool for the post-sequencing removal of contamination in metabarcoding studies. Environmental DNA. 2019;1(1):14–25.

41.	Liu C, White M, Newell G. Selecting thresholds for the prediction of species occurrence with presence-only data. Journal of Biogeography. 2013;40(4):778–89.

42.	Sing T, Sander O, Beerenwinkel N, Lengauer T. ROCR: visualizing classifier performance in R. Bioinformatics (Oxford, England). 2005 Oct 15;21(20):3940–1.

43.	Oksanen J, Blanchet FG, Friendly M, Kindt R, Legendre P, McGlinn D, et al. vegan: Community Ecology Package. Ordination methods, diversity analysis and other functions for community and vegetation ecologists. California, Berkeley, USA: The Comprehensive R Archive Network; 2016.

44.	McMurdie PJ, Holmes S. phyloseq: An R Package for Reproducible Interactive Analysis and Graphics of Microbiome Census Data. PLOS ONE. 2013 Apr 22;8(4):e61217.

45.	Zhao S, Guo Y, Sheng Q, Shyr Y. Heatmap3: an improved heatmap package with more powerful and convenient features. BMC Bioinformatics. 2014;15(Suppl 10):P16.

46.	Suzuki R, Shimodaira H. Pvclust: an R package for assessing the uncertainty in hierarchical clustering. Bioinformatics. 2006 Jun 15;22(12):1540–2.

47.	Burnham KP, Overton WS. Robust Estimation of Population Size When Capture Probabilities Vary Among Animals. Ecology. 1979 Oct;60(5):927–36.

48.	Linke S, Lehner B, Ouellet Dallaire C, Ariwi J, Grill G, Anand M, et al. Global hydro-environmental sub-basin and river reach characteristics at high spatial resolution. Sci Data. 2019 Dec;6(1):283.

49.	QGIS Development Team. QGIS Geographic Information System [Internet]. Open Source Geospatial Foundation; 2009. Available from: http://qgis.org

50.	Schneider A, Jost A, Coulon C, Silvestre M, Théry S, Ducharne A. Global-scale river network extraction based on high-resolution topography and constrained by lithology, climate, slope, and observed drainage density. Geophysical Research Letters [Internet]. 2017 [cited 2020 Nov 25]; Available from: https://hal.sorbonne-universite.fr/hal-01496152

51.	R Core Team. R: A Language and Environment for Statistical Computing [Internet]. Vienna, Austria: R Foundation for Statistical Computing; 2019. Available from: https://www.R-project.org/

52.	Ratnasingham S, Hebert PDN. bold: The Barcode of Life Data System (http://www.barcodinglife.org). Mol Ecol Notes. 2007 May 1;7(3):355–64.

53.	Froese R, Pauly D. FishBase. World Wide Web electronic publication [Internet]. 2010; Available from: www.fishbase.org

54.	Cantera I, Cilleros K, Valentini A, Cerdan A, Dejean T, Iribar A, et al. Optimizing environmental DNA sampling effort for fish inventories in tropical streams and rivers. Sci Rep. 2019 Dec;9(1):3085.

55.	Doble CJ, Hipperson H, Salzburger W, Horsburgh GJ, Mwita C, Murrell DJ, et al. Testing the performance of environmental DNA metabarcoding for surveying highly diverse tropical fish communities: A case study from Lake Tanganyika. Environmental DNA. 2020 Jan;2(1):24–41.

56.	Civade R, Dejean T, Valentini A, Roset N, Raymond J-C, Bonin A, et al. Spatial Representativeness of Environmental DNA Metabarcoding Signal for Fish Biodiversity Assessment in a Natural Freshwater System. PLOS ONE. 2016;11(6):e0157366.

57.	Pont D, Rocle M, Valentini A, Civade R, Jean P, Maire A, et al. Environmental DNA reveals quantitative patterns of fish biodiversity in large rivers despite its downstream transportation. Scientific Reports [Internet]. 2018 Dec [cited 2018 Jul 17];8(1). Available from: http://www.nature.com/articles/s41598-018-28424-8

58.	Dejean T, Valentini A, Duparc A, Pellier-Cuit S, Pompanon F, Taberlet P, et al. Persistence of Environmental DNA in Freshwater Ecosystems. Gilbert JA, editor. PLoS ONE. 2011 Aug 8;6(8):e23398.

59.	Strickler KM, Fremier AK, Goldberg CS. Quantifying effects of UV-B, temperature, and pH on eDNA degradation in aquatic microcosms. Biological Conservation. 2015 Mar;183:85–92.

60.	Curtis AN, Tiemann JS, Douglass SA, Davis MA, Larson ER. High stream flows dilute environmental DNA (eDNA) concentrations and reduce detectability. Yang J, editor. Divers Distrib. 2020 Nov 14;ddi.13196.

61.	McDevitt AD, Sales NG, Browett SS, Sparnenn AO, Mariani S, Wangensteen OS, et al. Environmental DNA metabarcoding as an effective and rapid tool for fish monitoring in canals. J Fish Biol. 2019 Aug;95(2):679–82.

62.	Hugueny B, Oberdorff T, Tedescco PA. Community Ecology of River Fishes: A Large-Scale Perspective. In: American Fisheries Society. K. B. Gido & D. A. Jackson; 2010. p. 29–62. (Community Ecology of Stream Fishes: Concepts, Approaches, and Techniques).

63.	Prance GT, Goulding M. The Fishes and the Forest: Explorations in Amazonian Natural History. Brittonia. 1981 Apr;33(2):257.

64.	Hubert N, Renno J-F. Historical biogeography of South American freshwater fishes. Journal of Biogeography. 2006;23.

65.	Goulding M. The Fishes and the Forest: Explorations in Amazonian Natural History. University of California Press. Berkeley, California; 1980. 280 p.

66.	Jaramillo-Villa U, Maldonado-Ocampo JA, Escobar F. Altitudinal variation in fish assemblage diversity in streams of the central Andes of Colombia. Journal of Fish Biology. 2010 May 18;76(10):2401–17.

67.	Anderson EP, Maldonado-Ocampo JA. A Regional Perspective on the Diversity and Conservation of Tropical Andean Fishes: Fishes of the Tropical Andes. Conservation Biology. 2011 Feb;25(1):30–9.

68.	Carvajal-Quintero JD, Escobar F, Alvarado F, Villa-Navarro FA. Variation in freshwater fish assemblages along a regional elevation gradient in the northern Andes, Colombia. 2015;13.

69.	Maurice-Bourgoin L, Alanoca L, Fraizy P, Vauchel P. Sources of mercury in surface waters of the upper Madeira erosive basins, Bolivia. J Phys IV France. 2003 May;107:855–8.

70.	Doria CRC, Duponchelle F, Lima MAL, Garcia A, Carvajal-Vallejos FM, Méndez CC, et al. Review of Fisheries Resource Use and Status in the Madeira River Basin (Brazil, Bolivia, and Peru) Before Hydroelectric Dam Completion. Reviews in Fisheries Science & Aquaculture. 2018 Oct 2;26(4):494–514.

71.	Anderson EP, Jenkins CN, Heilpern S, Maldonado-Ocampo JA, Carvajal-Vallejos FM, Encalada AC, et al. Fragmentation of Andes-to-Amazon connectivity by hydropower dams. SCIENCE ADVANCES. 2018;8.

72.	Finer M, Jenkins CN. Proliferation of hydroelectric dams in the Andean Amazon and implications for Andes-Amazon connectivity. PloS one. 2012;7(4):e35126.

73.	Lees AC, Peres CA, Fearnside PM, Schneider M, Zuanon JAS. Hydropower and the future of Amazonian biodiversity. Biodiversity and Conservation. 2016 Mar 1;25(3):451–66.

74.	Winemiller KO, McIntyre PB, Castello L, Fluet-Chouinard E, Giarrizzo T, Nam S, et al. DEVELOPMENT AND ENVIRONMENT. Balancing hydropower and biodiversity in the Amazon, Congo, and Mekong. Science (New York, NY). 2016 Jan;351(6269):128–9.

75.	Latrubesse EM, Arima EY, Dunne T, Park E, Baker VR, d’Horta FM, et al. Damming the rivers of the Amazon basin. Nature. 2017 Jun;546(7658):363–9.

76.	Forsberg BR, Melack JM, Dunne T, Barthem RB, Goulding M, Paiva RCD, et al. The potential impact of new Andean dams on Amazon fluvial ecosystems. PLOS ONE. 2017 Aug 23;12(8):e0182254.

77.	Freeman MC, Pringle CM, Greathouse EA, Freeman BJ. Ecosystem-Level Consequences of Migratory Faunal Depletion Caused by Dams. Biodiversity and Conservation of Shads Worldwide. 2003;35:255–66.

78.	Pringle CM, Freeman MC, Freeman BJ. Regional Effects of Hydrologic Alterations on Riverine Macrobiota in the New World: Tropical-Temperate Comparisons. BioScience. 2000 Sep 1;50(9):807–23.

79.	Duponchelle F, Isaac V, Van Damme PA, Herrera-R GA, Anderson EP, Cruz Rivetla EA, et al. Conservation of migratory fishes in the Amazon basin. Aquatic Conservation: Marine and Freshwater Ecosystems. in press.

80.	Shaw JLA, Clarke LJ, Wedderburn SD, Barnes TC, Weyrich LS, Cooper A. Comparison of environmental DNA metabarcoding and conventional fish survey methods in a river system. Biological Conservation. 2016 May 1;197(Supplement C):131–8.

81.	Doi H, Uchii K, Takahara T, Matsuhashi S, Yamanaka H, Minamoto T. Use of Droplet Digital PCR for Estimation of Fish Abundance and Biomass in Environmental DNA Surveys. Mahon AR, editor. PLoS ONE. 2015 Mar 23;10(3):e0122763.

82.	Pochardt M, Allen JM, Hart T, Miller SDL, Yu DW, Levi T. Environmental DNA facilitates accurate, inexpensive, and multiyear population estimates of millions of anadromous fish. Mol Ecol Resour. 2020 Mar;20(2):457–67.

83.	Dudgeon D, Arthington AH, Gessner MO, Kawabata Z-I, Knowler DJ, Lévêque C, et al. Freshwater biodiversity: importance, threats, status and conservation challenges. Biol Rev. 2006 May;81(02):163.

84.	Leroy B, Dias MS, Giraud E, Hugueny B, Jézéquel C, Leprieur F, et al. Global biogeographical regions of freshwater fish species. J Biogeogr. 2019 Nov;46(11):2407–19.

# Figure captions

### **Figure 1:** Map of the sampling locations

Map A shows the location of the 34 sites sampled and Map B focuses on sites south of the 13th parallel. The lake sites are represented by yellow disks (labelled with their full name), river sites are represented according to their hydrographic rank 1, 2 and 3 by red diamonds (samples 1-2), red squares (samples 3-17) and red triangles (samples a-g), respectively. Samples 6 and 7, as well as 12 and 13, were carried out at two sites on the Tuichi river in 2018 and 2019, respectively. Maps were created with QGIS v3.4 using the base map Stamen™ Terrain Background. The points were shifted for better viewing, but the actual positions are reported in the STable1. The hydro-ecoregions as defined by Wasson et al. (2002) are also indicated (see legend for details).

### **Figure 2:**

Number of taxa observed (green bars) and second order Jackknife estimate of taxa richness (blue bars) computed per biogeographical entities, lakes, rivers and overall sites (given the COI database). The orange bars represent the percentage coverage (ratio between the number of taxa observed and the Jack2 estimator) with black vertical error bars indicating the 95% confidence interval.

### **Figure 3:**

Maps showing taxon richness in the study area. For lakes (blue discs) the intensity of the colour is proportional to the observed richness (Mentiroso and Tumichucua lakes in the smaller box A). River sites are represented according to their hydrographic rank 1, 2 and 3 by diamonds, squares and triangles, respectively and a colour code associated with the biogeographic entities (BE) defined by the hierarchical analysis. The observed richness in rivers was projected and extrapolated on the hydrological network and represented by the colour code provided in the legend (network in red is not extrapolated).

### **Figure 4:**

Occurrence map representing the presence (blue) or absence (white) of taxa (rows) for each site (columns). Sites and species dendrograms are constructed using Jaccard's binary distance and a Ward clustering method. The colours associated with the site dendrogram correspond to the biogeographic entities defined by the hierarchical analysis. The colour associated with each leaf in the species dendrogram corresponds to its taxonomic Order. A detailed dendrogram of the taxa with their names is available in Figure S6. The names of the sites are those shown in Figure 1 (details in Table S3).

### **Figure 5 :**

Venn diagram showing the number of shared taxa between the four different biogeographic entities of the Tuichi. Note that only lakes in Madidi NP (Chalalán, Santa Rosa, and Ruta lakes) were included in this figure. Of the 186 taxa detected in the Madidi NP alone, only 7 occur in all biogeographical entities, while 14, 6, 6 and 54 were detected only in lakes, Alto-Tuichi, rank 3 rivers and rank 1-2 rivers, respectively. The values in brackets give the percentage of the total number of taxa detected. The drawings are not to scale.

### **Figure 6:**

NMDS ordination of the sampling sites based on the specific composition (Jaccard distance). Ordination was performed over all sampled sites including lakes and rivers (A), then using only rivers (B) or lakes (C). The convex hulls are colour coded according to the four biogeographic entities defined by the HAC. The sizes of the dot and triangle symbols for lakes and rivers respectively, are proportional to the number of taxa observed per site. Significant (p<0.01) continuous environmental variables are represented by grey arrows. The seven hydro-ecoregions are plotted as navy blue diamond points and corresponds to Glacial mountain (Cg), High peri-Amazonian Yungas (Ya-a), Amazonian gallery forest (Ab-a), Beni Gallery Forest (Ab-b), Dry valleys of the Yungas (Ay), High humid sub-Andean (Sa-a) and Low humid sub-Andean (Sa-b).

# Supplementary Data

Text S1: Scripts and command lines used for data analysis and plotting figures.

Table S1: Informations on sites and points of collect (date, collected volume, depth, coordinates ...).

Table S2: COI fish database used (fasta file).

Table S3: Environmental variables (soils, altitude, coordinate, geology, richness, Jack2 ...) .

Table S4: Sequencing results per library.

Table S5: Taxa abundance per library.

Table S6: Quality of assignation (%ID) + affinis.

Table S7: List of taxa present in each area of the Venn Diagram constructed at the cluster level.

Figure S1: Rarefaction curves at library and site level.

Figure S2: Frequency spectrum of taxa.

Figure S3: Taxonomic diversity at Order level per site.

Figure S4: NMDS ordination (site names plotted).

Figure S5: NMDS ordination including all sites. Orders are split into different facets.

Figure S6: Species dendrogram (Binary Jaccard distance and Ward2 aggregation).
