# Supplementary material for: Unveiling biogeographical patterns of the ichthyofauna in the Tuichi basin, a biodiversity hotspot in the Bolivian Amazon, using environmental DNA
Source: PLoS One. 2022 Jan 4;17(1):e0262357. doi: 10.1371/journal.pone.0262357 (PMC8726463; doi:10.1371/journal.pone.0262357)
Supplement: Attachment — Submitted filename: Responses to reviewers 09122021.docx [file pone.0262357.s019.docx]

**Reviewer #1: Review**

Comments to the Author

Environmental DNA (eDNA) approaches have been revolutionizing the assessment and monitoring of biodiversity in both aquatic and terrestrial ecosystems, yet their applications to rich biodiversity sites, especially in the Neotropical region, have largely been limited despite their potential. In this study, the authors assessed the potential of eDNA approaches for monitoring Neotropical fishes by analyzing eDNA from water samples collected both in rivers and lakes in the Bolivian Amazon region. Authors amplified DNA using a newly designed two COI metabarcoding primers targeting Amazon fishes and sequenced the amplicons on Novaseq illumina platform. Thus, they detected a high fish species richness and described their biogeographical distribution patterns related it to a set of ecological descriptors.

The topic of this manuscript fits to the scope of PlosOne, and it is timely and may potentially attract readers in both the field of Ichthyology and of biodiversity assessment using eDNA techniques. However, I found several points (detailed below), especially in the Method section that the authors should address to improve the clarity of the manuscript.

1/ **Abstract**

Abstract is over the 300 words limit.

Although authors should explain how the study was done without methodological detail, it would greatly strengthen the manuscript to inform in the Abstract the use of designed primers targeting Amazon fishes.

Answer : Done, the abstract has been reduced to 290 words and the mention to the primers included, as requested.

2/ **Introduction**

L60-61: “The Tuichi basin is included in the Madidi National Park and Natural Area of Integrated Management (hereafter abbreviated Madidi NP), a recognised biodiversity hotspot [8–10] and also likely hosts one of the few identified spawning area for many fish species [11]”

It is not recognized among the world’s 36 hotspots biodiversity. If it is only locally/nationally recognized I would suggest replacing the term “biodiversity hotspot” to a “rich biodiversity site”.

Answer : After verification in <https://www.cepf.net/our-work/biodiversity-hotspots/tropical-andes> we rephrased as follow for clarity:

“The Tuichi basin is part of the Madidi National Park and Natural Area of Integrated Management (hereafter abbreviated Madidi NP), which is included in the Tropical Andes hotspot of biodiversity”

3/ L68-86: Here authors include a brief review of the use of eDNA techniques including examples on amphibians, aquatic mammals, and fishes. However, they failed to current state of eDNA research in the Amazon region. I strongly suggest that authors strengthen this part. A key recent literature on the Amazon fish (Jackman et al. 2021) and mammals (Sales et al. 2019) might help.

Answer: done. The paragraph was modify to better take into account the most recent literature, especially on fish (Jackman et al. 2021, Sales et al., 2021, de Santana et al. 2021)

4/ L92-94: I would state specifically what your objectives were. - Why did you do this using eDNA? What were your objectives? The aim of the study as it is, seems to be a report case. Make clear the main aims of the study and how significant they are for the field.

Answer : done. The objectives of the study have been reformulated more clearly.

**Methods**

What is the average distance between the 34 collection sites?

Answer : Additional information have been add as follows:
“The average river network distance between sites collected in the Madidi NP is 14.4 km (SD=21.8).”

Did authors collect eDNA samples across years (2017, 2018, 2019) in the same 34 sites?

Answer : Each site has been collected only once. A clarification in this sense has been made:
“Environmental DNA samples were collected from lakes and rivers between September and October in 2017 (2 sites), 2018 (10 sites) and 2019 (22 sites); each site being sampled only once (see Table S1 for details).”

5/ L129-130: Include the License/authorization Number. Permits and approvals obtained for the work, including the full name of the authority that approved the study; if none were obtained, authors should explain why.
*eDNA field sampling*

Answer : rephrased as follows :
“The Ministerio de Medio Ambiente y Agua (MMAyA) of Bolivia approved the study and provided permits (authorization numbers: FCPV-IE-0196/2017; FCPV-IE-0030/2018; FCPV-IE-0064/2019) for collection[A1] , export and analysis of the filter cartridges to the French National Institute of Research for Sustainable Development (IRD) laboratories in France.”

Were temperature and pH recorded at each site?

Answer : No. In Table S3 column Temperature, data did not correspond to our sampling dates and were supposed to have been removed before submission (a single measure cannot be representative of potential seasonal variability). Sorry. We have now removed them.

Inform depth of sample water collection.

Answer : This information was added as follows :
“The average sampling depth is 1.15 m SD=0.6 (see Table S1 for details).”

Detailed information on the field and laboratory negative controls are missing. Make clear what is empty control. Please describe what was the field, “alien” and laboratory negative controls (commercial water, distilled water, or ultrapure water?). Additionally, please describe at which site and at which timing the field negative controls were collected and filtered. Also, detailed information on the sequencing platform is also required. I would guess that you used Illumina NovaSeq 6000, but please make it clear.

Answer : Corrected. The use of term “empty” was an error, it has now been replaced by ‘negative’ in order to refer to the negative field controls. We have also now specified that the negative laboratory controls (PCR and libraries) were carried out with ultra pure water.

The sentence explaining the origin of the controlled alien has been replaced in the eDNA field sampling method part.

The information concerning the dates and sites of collection of field negatives controls was available in Table S1 but not specified in the text. A mention is now made in the manuscript.

We have now specified the Illumina platform used (Novaseq 6000).

Did authors validate the designed primers and laboratory protocols before eDNA extraction?

In parallel to the analysis of the eDNA samples we used mock samples made up of 40 known taxa (DNA extracted from tissues) to assess the effectiveness of the primers used. We have now added this information in the methods.

Among the 40 taxa present in mock samples, 39 have been recovered by our primers, which validates their effectiveness. This information was already presented in the results, in “Controls by negative, positive and alien samples”

Furthermore, results obtained from the eDNA samples revealed an ichthyological diversity distributed over 11 Orders, 149 genera and 207 species, also highlighting the efficiency of these primers in detecting very distant taxas (from Myliobatiformes to Osteoglossiformes). These informations were already presented in the results in “Taxonomic diversity in the study area”

How did authors verify taxonomic resolution of COI fragments targeted by the designed primers? Did they use a database of Neotropical fishes to improve taxonomic assignments? With how many species?

Answer : (Added in S2 Text) :

In order to estimate the taxonomic resolution of MK1-185 bp and MK2-285 bp COI barcodes used in this study, an in silico analysis was first performed. We extracted all sequences of Amazonian species available in our COI database (Supp data), if they covered at least 90% of our MK1 and MK2 barcodes.

The MK1 dataset (503 species) is made up of 14 different orders, 46 families and 234 genera and MK2 dataset (543 species) is made up of 14 different orders, 46 families and 246 genera. These sequences were trimmed to remove the region corresponding to the primers thus generating fragments of 130 and 229 bp for MK1 and MK2, respectively.

These MK1 and MK2 sequence datasets were blasted against our COI database which contain 160,944 entries representing 3,480 genera and 15,429 fish species distributed worldwide.

Blastn results were parsed with MEGAN using a minimum identity parameter of 97% and a minimum score value of 100. Under these conditions, if a sequence is assigned to more than one reference in the database, the LCA (lowest common ancestor) is retained.

Among the 503 references extracted on the MK1 barcode, 428 were correctly assigned at the species level, 56 have been assigned to the correct genus and 19 to a higher taxonomic level than the genus.

Among the 543 references extracted on the MK2 barcode, 484 were correctly assigned at the species level, 39 have been assigned to the correct genus and 20 to a higher taxonomic level than the genus.

Then, taxonomic resolution at the species level was 85% and 89% for MK1 and MK2, respectively.

Moreover, as presented in the results, the proportion of OTUs assigned to the species level, which reached 90.5 and 92.5% for MK1 and MK2, respectively, confirm their high taxonomic resolution.

6/ **Results**

303-307: It is not clear how authors discarded the four taxa.

The accession number AMNHI782-12 (Marcusenius monteiri) was not found in GenBank. Make sure it is correct. And the other three taxa have their information matched in both GenBank and Boldsystem (but Epinephelus marginatus JX124778.1 was found in Bolsystem by its voucher LBPV53018).

How many reads do those four discarded taxa have? How is the author sure they were not putative contamination?

Answer: Thank you for this observation, indeed the accession number AMNHI782-12 is taken from Bold. A correction has been made.

We have rewritten this part for clarity as follows:
“After taxonomic assignment, 120599, 1418, 54 and 7 reads were aligned to *Marcusenius monteiri* (Bold : AMNHI782-12), *Epinephelus marginatus* (GenBank :JX124778.1), *Zoarces gillii* (GenBank : HM180941) and *Sebastiscus marmoratus* (GenBank : HM180872), respectively. As they correspond to marine or non-Amazonian taxa, we verified these 4 sequences by blasting them under BOLD / NCBI. The results clearly show that the names of these four accession numbers are erroneous as their sequences show high blast score and % identity with bacterial or human origin. They were, therefore, discarded.”

7/ L315-316: Did authors remove the maximum number of reads found in the controls from all samples before downstream the analysis to avoid putative contamination?

Answer : Yes, this was done with the microDecon R package (McKnight DT et al. 2019).

The following sentence has been added in the Material and method section :

“The three types of negative controls (field, extraction and PCR) were used to identify and remove contaminating reads in sample libraries with the R package Microdecon (McKnight DT et al. 2019), which uses the data in the 42 negative controls performed throughout the entire project to identify and exclude the reads in each library that correspond to contamination.”

8/ L323-324: It is a very low threshold to ensure non-contamination. Most eDNA studies remove all MTOUS <10 reads from the final dataset. Do authors have a citation for why 4 reads were adopted or just type a further clear justification.

Answer :

This 4 reads threshold was only applied as additionnal filter. We previoulsy applied two filters including :

- The package microDecon (McKnight DT et al. 2019, see point 7 for détails) in order to control contamination and false positives,
- A minimum number of reads by species and by library set on a threshold defined on mock controls (known composition in species) and corresponding to the optimal value maximizing specificity and sensitivity as mentioned in section “Data processing and taxonomic assignment”. This low-frequency threshold was 0.01%.

Then a 4 reads threshold based on the level of contamination (index breaks control) estimated using the Alien library was applied to remove all taxa with less than 4 reads. This is because in all the Amazonian libraries, the maximum number of reads of a species present in the alien samples was 3.

This last point above has been clarified in the manuscript in section “Controls by negative, positive and alien samples”.

Note that among the 252 taxa identified in the study area only 11 taxas (2 at the genera level and 9 at the species level) have less than 10 reads.

9/ L332-333: One of the cost-efficiency of using eDNA is to detect rare or cryptic species that are difficult to detect by using traditional methods. However, it is highly prone to putative contamination. Since key information on the Methods section is missing, I am not truly convinced that these 16 named affinis are not putative contamination. How many reads do those taxa have?

As mentioned by the reviewer, we agree that eDNA metabarcoding studies is prone to putative contamination. More information is now available in the Material and methods to explain the different procedures applied to control for contamination, such as the three threshold filters detailed in the previous comment (point 8).

We have added the following information in the result section “*Taxonomic diversity in the study area*”: “Among the 16 species named “affinis”, 11 have an abundance of more than 193 reads after treatment with microDecon (deletion of reads on the basis of negative controls), while the maximum number of reads observed in the 42 negative controls is 3 for these 16 species (see table below). Contamination can therefore be excluded during the collection and / or laboratory steps.”

9/ L332-333: Is it possible they have been introduced in the Amazon basin?

Although such introductions have not been documented in the Beni watershed, except for *Arapaima gigas* (which is not an “affinis” species), they cannot be ruled out.

The discussion (“*Human threats and conservation*”) was completed to take into account the existence of introduced species.

**Discussion**

10/ Authors should put their results in the context with respect to other eDNA studies on fish surveys in the Amazon and Neotropical region. It would greatly strengthen the discussion to briefly detail the survey success obtained in this study to the success of other studies.

Answer: ok, done

11/ Authors should discuss in more detail the conservation issues regarding the species found in the study site, especially regarding the newly detected species.

The discussion was completed about the newly detected species

Are there any threatened species?

None, few information is available about the 252 identified taxas among the IUCN red list of threatened species. Status information is available for only 38 of them. 8 are DD, 29 are LC, 1 is NT (*Imparfinis cochabambae*).

This lack of information is so extensive that it seems irrelevant to consider a discussion on the conservation status for the taxas identified in our study.

Is it possible for any of those affinis species to be an exotic species introduced in the area? Prochilodus argenteus, for example, is an invasive species in several Brazilian river basins. Is it possible for the species to be present in the study site?

Although introduction cannot be ruled out, it is likely for only one species: Prochilodus argenteus. A small paragraph was added in the discussion to take that into account.

English

12/ The level of English is very high throughout the proposal, but there are some minor errors throughout. It would therefore be worthwhile having the final version carefully reviewed before final submission.

The manuscript has been checked for language mistakes.

13/ Figure 3: see comments in the pdf file.

Corrected : a short section of a river had a color code associated with the extrapolated number of species inconsistent with the sampled sites

14/ Figure S3: Taxonomic diversity at Order level per site: Axis is very difficult to read. I suggest increasing the text size, or include this figure in an entire page.

The size of the figure S3 has been increased for easier reading

15/ Minor comments are in the main text pdf file. Good luck revising the manuscript and I look forward to seeing the revised version!

[NOTE: If reviewer comments were submitted as an attachment file, they will be attached to this email and accessible via the submission site. Please log into your account, locate the manuscript record, and check for the action link "View Attachments". If this link does not appear, there are no attachment files.]

While revising your submission, please upload your figure files to the Preflight Analysis and Conversion Engine (PACE) digital diagnostic tool, <https://pacev2.apexcovantage.com/>. PACE helps ensure that figures meet PLOS requirements. To use PACE, you must first register as a user. Registration is free. Then, login and navigate to the UPLOAD tab, where you will find detailed instructions on how to use the tool. If you encounter any issues or have any questions when using PACE, please email PLOS at figures@plos.org. Please note that Supporting Information files do not need this step.

[A1]Include the License / authorization Number. Permits and approvals obtained for the work, including the full name of the authority that approved the study; if none were obtained, authors should explain why

Done : the sentence has been rephrased for clarity. The requested information relating to the authorities and permit numbers are now more readable.
